# Development of an Administration Guideline of Oral Medicines to Patients with Dysphagia

**DOI:** 10.3390/medicina59111913

**Published:** 2023-10-29

**Authors:** Kersti Teder, Juri Karjagin, Kairi Marlen Antoniak, Marika Saar, Daisy Volmer

**Affiliations:** 1 Institute of Pharmacy, Faculty of Medicine, University of Tartu, Nooruse 1, 50411 Tartu, Estonia; 2 Pharmacy Department, Tartu University Hospital, L. Puusepa 8, 50406 Tartu, Estonia; 3 Institute of Clinical Medicine, University of Tartu, L. Puusepa 8, 50406 Tartu, Estonia; 4 Department of Anaesthesiology and Intensive Care, Tartu University Hospital, L. Puusepa 8, 50406 Tartu, Estonia; 5 Pharmacy Department, East-Tallinn Central Hospital, Ravi 18, 10138 Tallinn, Estonia

**Keywords:** medication therapy management, oral administration, deglutition disorders, pharmaceutical preparations, delivery of healthcare

## Abstract

*Background and Objectives*: There is increasing evidence that patients with dysphagia often have limited access to suitable oral dosage forms, especially when administered via an enteral feeding tube (FT). In addition, there is a lack of clear and readily available information from drug manufacturers on how to administer medications to patients with dysphagia. This study aimed to develop a practical guide for healthcare professionals to increase the safe and effective administration of oral medications to patients with dysphagia. *Materials and Methods*: The data were collected from existing English databases and handbooks available to develop an easy-to-use tabular guideline presenting all relevant information using keywords and short expressions. The working group differentiated 514 formulation types, and the information was collected and added to the guideline separately. In addition, the instructions for the patients taking the medicines orally or via FT were described separately. *Results*: The guideline consisted of 24 keywords or short expressions developed by the working group and described the instructions to use them. The guideline contained 343 active pharmaceutical ingredients and 19 fixed-dose combinations. *Conclusions*: Knowledge about proper medication preparation and administration for patients with swallowing difficulties is limited but essential. It is crucial to encourage drug manufacturers to provide this information as a standard to ensure the safe and effective use of medications for all patient groups.

## 1. Introduction

Oral administration is the most preferred way of medication administration by patients [1]. However, increasing evidence indicates that for many patients, it is complicated due to swallowing problems [2]. Especially people with dysphagia have limited access to suitable oral dosage forms, and the available ones often need alterations like cutting, crushing, and dispersing, especially when the medicines are administered via an enteral feeding tube (FT) [3].

There is a lack of clear and readily available information from drug manufacturers on ways to administrate medications to patients with dysphagia [4,5]. Although some handbooks and databases provide guidelines about medication management for these patients, much such information is unavailable [5,6,7,8,9]. Besides legal issues, medication alterations can lead to several problems, like changes in absorption and effects, recurrent adverse drug reactions, FT blockages and drug loss, or an unpleasant taste that can cause reluctance to take the medicines [10,11,12]. To prevent these complications, healthcare professionals (HCP) need specific guidelines and training to prescribe, prepare, and administer appropriate drug formulations to patients with dysphagia, including patients with FTs [4,13,14,15,16,17,18,19].

This study aimed to analyse the information availability and consistency on ways to administrate oral medications to patients with dysphagia. Furthermore, the final goal was to develop a practical guide to improve the safe an effective administration of oral medications to patients with dysphagia, either by mouth or via FTs.

## 2. Methods

The information search and guideline development were initiated and performed by the Estonian Society of Hospital Pharmacists (ESHP). The working group consisted of hospital and clinical pharmacists from three major hospitals in Estonia (East-Tallinn Central Hospital (ETCH), North-Estonian Regional Hospital (NERH) and Tartu University Hospital (TUH)). 

The final goal was to develop an easy-to-use tabular guideline, presenting the relevant information in the Estonian language using keywords and short expressions [20,21], similar to the guidelines used in two Estonian hospitals (NERH and ETCH). However, the guidelines could not be directly transposed since these covered only some of the information and active pharmaceutical ingredients (APIs) suggested by the working group. 

Promotion of the available international databases, handbooks, and guidelines was not considered due to the language barrier and insufficient selection for the required APIs or formulations [6,7,8,9,10,11,12,13,14,15,16,17,18,22,23,24]. However, these were used as references.

**Figure 1:**
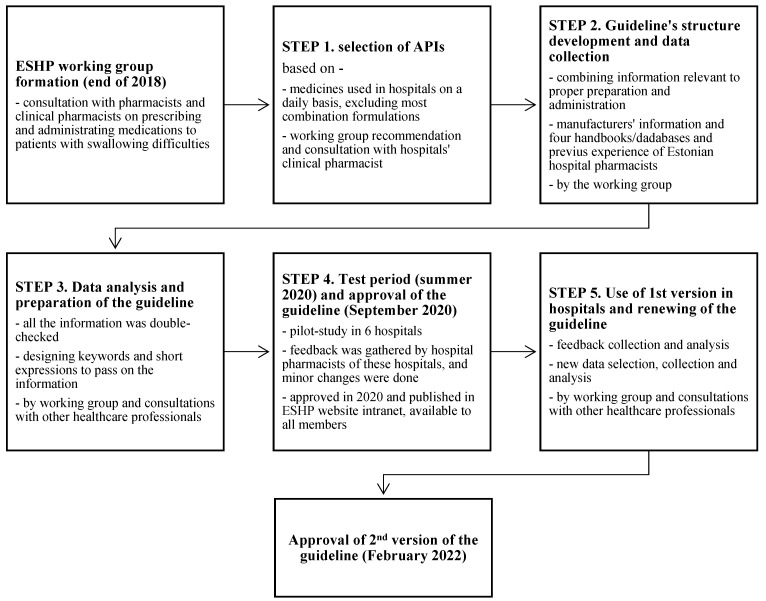
Schematic presentation of the guideline development and renewal process (API—active pharmaceutical ingredient; ESHP—Estonian Society of Hospital Pharmacists).

The complete guideline development and renewal process is described briefly in Figure 1.

### 2.1. Step 1. Selection of APIs

API selection was based on oral medicines used daily in hospitals (collected from ITCH, NERH and TUH), excluding the combination formulations except if the APIs were not available in single formulations and were relevant for in-hospital use. 

### 2.2. Step 2. Selection and Collection of the Data

**Figure 2:**
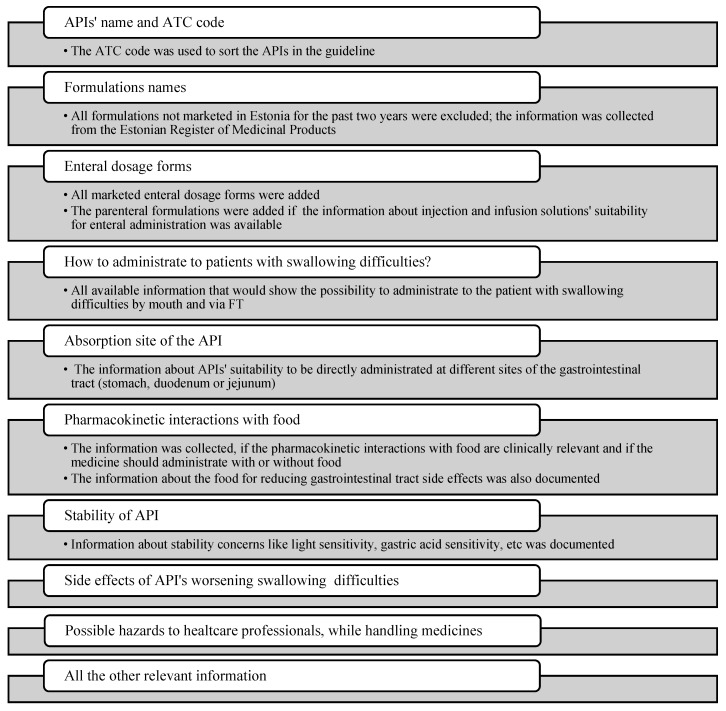
Information details gathered during the first round of data collection (API—active pharmaceutical ingredient; ATC—Anatomical Therapeutic Chemical).

The collected information with short comments is presented in Figure 2. The aim was to cover all specific information needed to prescribe, prepare and administer the medicines for patients with dysphagia, either by mouth or via FTs.

The first information source was the Estonian Registry of Medicinal Products (ERMP), where information on all the registered formulations is available [25]. The information from summaries of product characteristics (SmPCs) and patient information leaflets (PILs) was collected. However, sources such as official medicine registries from other countries or manufacturer product web pages were searched to find information for unlicenced formulations.

In addition, the information from 4 publishers (as handbooks or databases) available to the working group was collected [6,7,8,9,22,23,24]. And the data from local guidelines, including unpublished results from tests performed by Estonian hospital pharmacists, were added to the dataset. 

All the collected information was added to the dataset created using Microsoft Excel.

### 2.3. Step 3. Data Analysis

At least two pharmacists double-checked the data, which were added to the guideline if there were no uncertainties. All uncertainties were discussed among the working group members, and a joint decision was formed by mutual agreement. If needed, other HCP were involved in the discussions. 

Using keywords and short expressions, the guideline was formed as a Microsoft Word table. In addition, all records were cited. Finally, instructions for the use of the guideline were written, and the used keywords and expressions were defined. 

### 2.4. Step 4. Test Period and Approval of the First Version of the Guideline

Before publishing, six Estonian hospitals agreed to pilot the guideline. The guideline draft was sent to hospital wards that often have patients with dysphagia. The local hospital pharmacists collected feedback from nurses in two months and sent it to the working group. The feedback and questions were analysed. After minor adjustments, the guideline was published as a PDF document in September 2020 on the ESHP website, available to all ESHP members who could distribute it in their hospitals.

### 2.5. Step 5. Planning and Preparation of the Second Version of the Guideline

**Figure 3:**
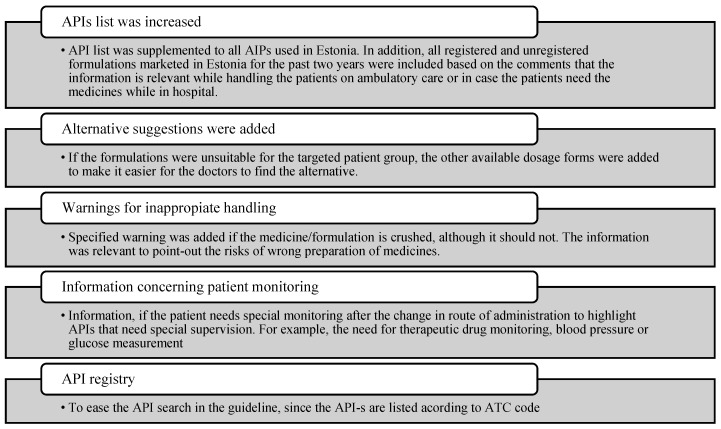
The information added into the second version of the guideline (API—active pharmaceutical ingredient).

After the release of the first version, the users were encouraged to provide feedback to the working group. All questions and comments received in one year were documented in an unstructured way by the guideline contact person. Questions and comments were sorted by qualitative content analysis, analysed and discussed by the working group. Finally, it was decided that the information presented in Figure 3 should be added to the guideline, and the rest was classified as “should be solved by training”.

The ESHP working group collected missing data. Procedures and references were like those of the first time. 

## 3. Results

### 3.1. Structure of the Guideline

The guideline was developed as a table containing 11 columns: ATC-code, active substance, medicinal product, dosage form, oral administration guide and comment, administration by FT guide and comment, site of absorption/administration, administration with food, remarks; the entire guideline is presented as Online Resource 1.

The keywords (e.g., crush, disperse) or short expressions (e.g., open capsule, regardless of food) were used. Twenty-four keywords or expressions were developed, and for clarity, these were defined in the guideline instructions for use. The instructions for use also contained column descriptions, some statements on the responsibility of the HCPs, things to consider when prescribing, preparing or administering medicines and some comments about occupational hazards. 

### 3.2. Data in the Guideline

The guideline data statistics are presented in Table 1.

#### 3.2.1. Anatomical Therapeutic Chemical (ATC) Code

The APIs were listed according to the World Health Organisation’s ATC code system, which enables the grouping of similarly acting APIs and makes it easier for HCPs to find alternatives for unsuitable or unpreferable formulations. 

The alphabetic registry of APIs was added to the second version guideline to ease the search.

#### 3.2.2. Names of APIs and Formulations

The second version of the guideline consisted of 343 APIs and 19 fixed-dose combinations (an overall 31% increase compared to the first version). The low number of fixed-dose combinations was due to their limited use in hospitals, primarily due to cost effectiveness. 

The formulation names were presented in the guideline as a request from nurses, and in addition, these enabled the demonstration of the known differences between formulations. Since each API might have more than one dosage form, 514 different formulation types (different APIs in various dosage forms) were identified.

#### 3.2.3. Dosage Forms

Altogether, 38 different dosage forms were presented in the guideline. On average, one API had 1.4 dosage forms (median was 1, interquartile range 1–2; maximum 7). As expected, the conventional release tablets were the most common dosage form. Seven oro-mucosal formulations were added to the guideline since these could serve as an alternative to oral administration [26].

Data about injection or infusion solution suitability for enteral use was also collected. The information was added to the second version with guiding comments in the instructions section. Although the information was available for 30 APIs, the injection formulation was added for 19 APIs; the others were left out since there were no parenteral formulations in Estonia, or it was found irrelevant. In the case of sodium chloride, injection form was the only option for oral administration. And theophylline could be substituted with an aminophylline injection solution.

#### 3.2.4. Formulation Preparation for Administration

As mentioned previously, the working group differentiated 514 formulation types. The data about these formulation types were collected and added to the guideline separately (on a separate data line). In addition, the patients’ instructions for taking the medicines orally or via FTs were brought out separately. As we compared the oral and FT administration data, we observed that 163 times, the possibility of administering or preparing instructions were different (Table 1).

In addition to the specific guidelines (how to crush, disperse, open, etc.), relevant comments were added. For example, in case of oral administration, information about the bitter taste of the medicine, the local anaesthetic effect in the oral cavity, and the possibility of mixing the medication with food were included. For FT administration, if known, the size of the suitable FT or a warning of the higher risk of FT obstruction was added.

#### 3.2.5. Administration Site

Since FTs can be placed in different locations, the information about absorption sites of APIs was also searched. In case of 16 APIs, the absorption in the gastrointestinal tract was not possible or was very low, and the therapeutic effect was local. From others, the absorption site was known for 28% of the APIs. The overall suitability of APIs for FT administration was also investigated, especially for duodenal and jejunal administration (Table 1). However, information about it was often unavailable. 

#### 3.2.6. Food Interactions

The relevance of food interactions with the selected APIs was also analysed (Table 1). Unfortunately, very little information about the influence of food on medicine pharmacokinetics was available, and more was available about when the medication should be taken, considering the feeding time. Moreover, this information was sometimes controversial when different references were considered. 

#### 3.2.7. Additional Remarks

To ensure the drug and patient safety, the information about the stability of the API (e.g., light sensitivity or degradation due to gastric acid), consequences of the wrong alteration of the medication and possible side effects concerning swallowing difficulties (e.g., dry mouth, taste disturbance, mouth ulcers) were collected and brought out (Table 1). 

Occupational hazards due to tablets crushing and capsule opening were also analysed. For 23% of APIs, possible hazards to HCPs were mentioned in references, but not consistently. Since evidence on the occupational hazard was unclear, the information from the NIOSH List of Hazardous Drugs in Healthcare Settings (NIOSH list) was also collected and analysed [27]. Finally, 7% of APIs in the guideline were classified as hazardous (13 APIs known to be potentially cytotoxic and mutagenic and could be teratogenic) or hazardous to pregnant people (12 APIs known to be teratogenic). Special safety procedures were described to lower the risk of exposure. 

### 3.3. Overall Information Availability and Interpretation

The manufacturer information about drug administration to patients with dysphagia (including administration via FTs) was lacking for 183 (36%) formulation types. Additionally, in the case of 81 (15% of all) formulation types, all or at least some manufacturers stated that the formulation should be taken as a whole. In more than half of cases, the reason for that was unclear, especially for conventional release tablets and capsules (57 formulation types).

In the case of other references, The NEWT Guidelines: for Administration of Medication to Patients with Enteral Feeding Tubes or Swallowing Difficulties did not have any information for 87 APIs; this number was 114 for the Handbook of drug administration via enteral feeding tubes (PPH), 62 for the Australian “don’t rush to crush” handbook (DRtC) and 33 for the UpToDate database. There were 15 APIs that did not have information in any selected references. However, the presence of APIs in the handbooks and databases did not ensure information availability in all dosage forms. The reference might have described its capsule’s handling, and the information was needed for the tablet or vice versa. 

In addition, the working group identified 151 (29%) formulation types for oral and 141 (27%) for FT administration, where only one of the four references had relevant information. However, if the information was available in more than one reference, the information could be controversial: 71 times (14%) for oral and 84 (16%) for FT administration. 

The second version of the guideline (Online Resource 1) was published as a PDF document on the ESHP website (https://ehas.ee/juhendid, accessed on 1 March 2022) and is freely available.

## 4. Discussion

The prevalence of patients with dysphagia in Estonia has not been widely studied, and the system to diagnose and treat these patients is still under development [28]. This project aimed to develop an easy-to-use tabular guideline for HCPs presenting all relevant information for oral medications used by patients with dysphagia. A small local study in TUH [5] and an additional literature review have demonstrated the need for the guideline described above. 

Studies have shown that the guidelines are not consistently implemented in practice due to their complexity [20,21]. Based on the previous practical experience of the working group, the Estonian nurses preferred the tabular guidelines with short and precise instructions. In the guideline, all information was stated as concisely as possible, without losing clarity of the content (Online Resource 1). A similar system was used in some handbooks and databases if the medication preparation technique was referred to [7,8].

### 4.1. Data Collection and Availability

During data collection, it became evident that the decisions on whether and how the medications can be administered to patients with dysphagia should be based on APIs and dosage forms. The same system was often used in handbooks and databases, and manufacturer formulation-specific information was lacking. Though in PPH, some information was also based on medicinal products, and in DRtC, the medicinal product names were stated at the beginning of the chapter (though generics were often mentioned without formulation names) [6,7]. 

With the instructions based on APIs and dosage forms, the construction of the guideline was relatively easy. However, since API same dosage forms may act differently in different medicinal products (can be differently manufactured and consist of various excipients), the confidence of the working group in the suitability of the instructions for all medicines was decreased. For example, metoprolol prolonged-release tablets from different manufacturers do not have the same instructions for use in the guideline. Some formulations can be dispersed, and others cannot (Online Resource 1, page 16), probably due to the different designs. In addition, information in different references was controversial in about 15% of the cases. In these cases, one reference stated that tablets could be dispersed, and the other suggested that the tablets should be crushed. However, no information was available if the difference in recommendations was based on the differences in formulations or tests or lack of relevant information. In such cases, the working group often recommended “dispersion of the tablet” as a safer way of preparing the medicines [6,7,8]. 

All the concerns described above increased a perception that the details about medicines administration should come from manufacturers. Moreover, providing such information should not be optional. Regulatory affairs should ask for this data from all drug manufacturers, which should be presented in PILs and SmPCs as a standard section. Stewart et al. (2016) and Shariff et al. (2020) even further recommended that the needs of patients with swallowing difficulties should be considered when the medicines are in the developing stage [1,29].

One of the most challenging parts of developing the guideline was the evaluation of occupational hazards while altering the medications. Although safety concerns are often discussed in the literature, the instructions for safety measures or medication lists of what should be considered harmful for handlers vary [6,7,8,9,14,18,27]. Considering all this, the working group suggested using protective gloves in all medicine manipulations and facial masks in case of antibiotics and hormones. In addition, the guideline highlights APIs that are hazardous to all (e.g., cytotoxic, mutagenic) or pregnant (teratogenic) handlers. Since there are no national lists of hazardous medications to handlers in Estonia, these 25 APIs were highlighted after analysing the NIOSH list and manufacturer information through the ERMP [25,27]. However, the working group also highlighted a need for more apparent evidence and a national strategy for these medicines. Therefore, if available, national or hospital guidelines should be considered. 

### 4.2. Strengths and Weaknesses of the Study

The guideline is freely available for all Estonian HCPs to help in prescribing, preparing and administering medications for patients with swallowing difficulties, either by mouth or via FTs

Although it is on the website of the ESHP, there is a need for an implementation plan [4]. Similar studies have shown the benefits of such written instructions [14,18], but further research is needed to evaluate whether the ESHP’s guideline improves the treatment outcomes of the targeted patients. Compliance with the guideline should be assessed, and boundaries leading to mishaps should be analysed. Since the guideline has just a short theoretical introduction, overall prescription and treatment planning recommendations are needed. 

The guideline is based on a limited amount of manufacturer information and instructions available in the stated handbooks and databases and practical experiences of hospital pharmacists in Estonia; a more comprehensive range of references should be used in the future. For example, information from the original research should be collected and analysed. In addition, further communications with drug manufacturers to collect the data available to them that currently are not included in PILs and SmPCs might increase the certainty of the recommendations. Also, well-planned in vitro tests, similar to Bessera et al. (2017), should be performed to see whether tablets can be dispersed reasonably [30]. Dissolution characteristics of altered modified-release formulations should be studied to predict possible pharmacokinetic changes.

Although the entire guideline is available as Supplementary Materials of this article (Online Resource 1), it should be noted that the APIs and formulations presented in the ESHP guideline are relevant in Estonia and may not be used or available in other countries. Some APIs and formulations might be missing since the availability of different formulations in Estonia are somewhat limited, even if they are registered in Estonia [5]. This also limits the suggestions for alternatives—for example, the possibility of using extemporaneous formulations was often suggested by other references [6,7,8,22,23] 

In addition, the guideline contains mainly medicines with one API, but the fixed-dose combination formulations are included only as an exception (19 formulations). Since the prevalence of fixed-dose combination formulations is increasing, it is already planned to collect and publish the information in the next version of the guideline. Although the present suggestion, due to the limited data, is to use separate formulations, the working group members accept that it might be safer and more convenient to use one fixed-dose combination formulation instead of two different medicines.

## 5. Conclusions

The ESHP guideline “Administration of Medicines to Patients with Swallowing difficulties: Orally or by Enteral Feeding Tube” (Online Resource 1) is the first freely available guideline for all Estonian HCP to help in prescribing, preparing and administering medications for patients with swallowing difficulties, either by mouth or via FTs. 

Knowledge about proper medication preparation and administration for patients with swallowing difficulties is limited but essential. It is crucial to encourage drug manufacturers to provide this information as a standard to ensure the safe and effective use of medications for all patient groups.

## Figures and Tables

**Table 1:** The guideline data statistics.

Data Fields	Statistical Information	
ATC code	The guideline contained the active substance from 13 ATC code groups. - Most (>30) APIs were from four groups: N-group—90 APIs; C-group—60 APIs; A-group—53 APIs; J-group—49 APIs.	
API	The guideline included 343 single APIs and 19 API combinations. - A 31% increase compared to the first version; 81 APIs and 5 API combinations were added; 1 API was removed.	
Dosage form	The guideline contained 38 different dosage forms. - One API usually had 1 dosage form (median was 1, interquartile range 1–2; maximum 7). - Tablets (including coated and uncoated) were the most common dosage form—262 (51%); APIs had tablets as a dosage form. - 69 (25%) APIs had at least one dosage form as a liquid or that could be turned into liquid. - Liquid form (42 (15%); e.g., a solution, suspension. - Required liquification before administration (27, e.g., a powder for oral suspension or effervescent tablet) - 94 modified formulation types were identified: most prolonged-release tablets. - 7 oro-mucosal dosage forms were added to the guideline. - For 30 APIs, the injection/infusion dosage form was a possibility for enteral use; for 19 APIs, it was added to the guideline.	
Medicinal products	The guideline consisted of 514 formulation types (different APIs in different dosage forms).	
Guide	Oral administration - 82 formulation types had no information. - 151 (29%) had only one information source. - 71 formulation types had controversial information. - 107 formulation types should not be used. - 407 formulation types can be used. - 120 formulation types should be crushed before use, and 119 should be dispersed. - 52 capsules can open. - 29 modified formulation types can be used. - 121 formulation types should not or cannot be crushed or chewed.	Administration by enteral FT - 88 formulation types had no information. - 141 (27%) had only one information source. - 82 formulation types had controversial information. - 153 formulation types should not be used. - 361 formulation types can be used. - 118 formulation types should be crushed before use, and 128 should be dispersed. - 37 capsules can open and pass properly through FT size. - 14 modified formulation types can be used. - 143 formulation types should not or cannot be crushed.
Comment	- 254 formulations had information about the possibility of thickening or mixing with food. - 49 formulations had information about their bad or bitter taste, and 8 (2%) formulations had information about their numbing effect on the mouth.	- 35 formulations had information about a higher risk of FT obstruction. - 13 formulations should be prepared by shaking before administration. - 93 dispersible formulation types had their dispersing time stated. - 107 of the formulations had information about suitable tube size.
The place for absorption/administration	16 (4%) APIs could not absorb, or the absorption was very low, and the effect was local. 98 (28% if relevant) APIs had information about their absorption site. - 30 (1/3 of known) APIs are absorbed in the small intestine or its parts. - for 6 APIs, gastric acid and for 4 APIs, the bile salts were stated as relevant for dissolution or absorption. 77 APIs had information about duodenal administration. 133 APIs had information about jejunal administration.	
Administration with food	11 APIs had no information about interactions with food or if the drug administration is influenced by food intake. 180 (52%) APIs can be taken regarding food. 64 (18%) APIs should or can be taken with food to reduce GIT side effects.	
Remarks	9 (2%) APIs had physical interaction with other medicine and should be taken separately. 31 (9%) APIs had stability issues (like sensitivity to light, acid, water, etc.). 111 APIs might have swallowing difficulty, worsening side effects. 85 APIs had a possible hazard mentioned to healthcare professionals; 25 APIs were brought out as hazardous or potentially hazardous to healthcare professionals.	

## Data Availability

The datasets generated and analysed during the current study are available from the corresponding author on reasonable request.

## Supplementary Materials

The following supporting information can be downloaded at: https://www.mdpi.com/article/10.3390/medicina59111913/s1, The ESHP guideline: Administration of Medicines to Patients with Swallowing Difficulties: Orally or by Enteral Feeding Tube.
