# Supplementary material for: Development of an Administration Guideline of Oral Medicines to Patients with Dysphagia
Source: Medicina (Kaunas). 2023 Oct 29;59(11):1913. doi: 10.3390/medicina59111913 (PMC10673523; doi:10.3390/medicina59111913)
Supplement: Supplementary file 1 [file medicina-59-01913-s001.zip › medicina-2657861-supplementary.pdf]

Administration of Medicines to Patients with Swallowing Difficulties: Orally or by Enteral Feeding Tube

Foreword

This guideline was compiled for situations where there is a need to administer oral medications to patients with swallowing difficulties. In such cases, medications' alteration (dissolving, dispersing and crushing) is often needed, although at the same time it is often off-label use and is not described in the medication's Summary of Product Characteristics (SmPC). However, there are situations in clinical practice where it is necessary to considered alterations.

Hospital pharmacists from the Estonian Society of Hospital Pharmacists (ESHP) have developed this guideline as a practical tool for all healthcare professionals dealing with pharmacotherapy for patients with swallowing difficulties, including the ones using enteral feeding tubes (FT).

Various international written sources and the practical experience of Estonian hospital pharmacists have been used in compiling this guideline.

Naturally, this guideline does not cover all situations that occur in clinical practice, so the advice of a hospital pharmacist should be sought in such situations. All questions, suggestions and comments to the authors that arise during the use of the guideline are very welcome.

We hope this guideline proves useful!

The first version was composed by:

Liisa Eesmaa, East Tallinn Central Hospital Pharmacy, pharmacist

Krista Meresmaa, North Estonia Medical Centre Pharmacy, clinical pharmacist

Kersti Teder, Tartu University Hospital Pharmacy, pharmacist

The second version of the guideline was supplemented by:

Kairi Marlen Antoniak, East Tallinn Central Hospital Pharmacy and Tartu University Hospital clinical pharmacist

Krista Meresmaa, North Estonia Medical Centre Pharmacy, clinical pharmacist

Kersti Teder, Tartu University Hospital Pharmacy, pharmacist

General principles

Responsibility

The users of this guideline need to read the information in the table together with the explanatory part of the guideline. Please note that the manipulation of the formulation may be against the manufacturers' information (off-label use) and alter both the pharmacokinetics and the effect of the medicine.

Take this into account when prescribing the medicine

- Can the administration of the medicine be stopped for the duration that the patient has swallowing difficulties?
- Does the active pharmaceutical ingredient (API) have a formulation suitable for administration via a FT?
  - Crushing the tablet's/capsule's contents should always be the last resort.
  - In case of swallowing difficulties, the liquid formulation may not always be suitable. Therefore, the selected consistency of the medicine to be administered should be similar to the consistency of the food.
- Can the formulation, route of administration or API be changed?
- Can the number of administrations be reduced?
- In case of any changes, the dose and frequency of administration should always be reviewed.
- Changes in the formulation may affect bioavailability and thus the medicine's effect. Oral formulations (e.g. long-acting and short-acting, solid and liquid) may not be equivalent. If possible and appropriate, monitor the plasma concentration of the API or evaluate the effect by other means (measure INR, blood glucose etc.).
- When administering medications via FTs, consider the location of the FT's tip and the site of medicine's absorption.

Take this into account when preparing and administering the medicine

- Prepare the medicine immediately before administration.
- Use syringes with an ENFit tip (syringes for enteral administration that are not compatible with intravenous systems).
- Prefer dispersing the medicine to crushing it.
- When administering the medicine via a FT, prefer to disperse the medicine directly in the syringe unless instructed otherwise.
- When crushing, prefer a closed and easy-to-clean crusher.
- The water (e.g. tap water, bottled water, water for injection) used for administering the medications and rinsing the FT should be selected according to the hospital's infection control guideline and be at room temperature.
- All medicines must be prepared and administered separately.
- The FT should always be rinsed before and after administering the medication and between each administration. The amount of liquid used for rinsing the FT is usually equal to twice the volume of the FT (10-30ml).
- If the patient has a fluid restriction, try using less fluid than instructed.
- Medicines should not be mixed into the enteral tube feed.
- Medicines for patients with swallowing difficulties may be mixed into a small amount of food at room temperature or a liquid with suitable viscosity.
- The total amount of fluid involved in administering the medications should be accounted for in the fluid balance.
- Medications with an anaesthetics/numbing effect in the mouth should be taken at least 30 minutes before or after a meal to prevent the deterioration of the swallowing function. If possible, the mouth should be rinsed after the administration of the medication.

Safety during the preparation of the medicines

The manipulation of the medications may result in increased exposure to the API compared to regular handling. The healthcare professional must therefore be protected from this exposure (incl. potential health risks) by using appropriate personal protective equipment.

Take this into account when preparing for administration.

- Always wear protective gloves.
- If possible, prefer dispersing to crushing to reduce direct contact with the medicine,.
- When possible, prefer closed crushing devices to mortars to reduce the formation of medicine dust.
- It is recommended to use a protective mask (FFP2 type II) whenever crushing medicines (especially anti-infectives, hormones, immunosuppressants).
- The table lists separately medicines that are potentially dangerous for all healthcare professionals and particularly pregnant handlers:

  HAZARDOUS DRUG (the name box of the API is yellow) – the API is potentially mutagenic and carcinogenic and may also be teratogenic or toxic to reproduction; crushing/dispersing the tablet and opening the capsule is permitted only if the specific measures are taken to reduce the risks to the safety of personnel and the contamination of the working environment:

  - use an FFP3 class respirator and nitrile protective gloves;
  - implement measures to reduce the contamination of the work surface and the working environment (e.g. biosafety cabinet, closed system, work surface preparation and cleaning).

  If the above requirements cannot be met, consult the hospital pharmacist.

  HAZARDOUS TO PREGNANT (the name box of the API is light yellow) – the API is potentially dangerous due to teratogenic effects; pregnant healthcare professionals and those planning to become pregnant should wear a Class FFP3 respirator and nitrile gloves when crushing/dispersing the tablet or opening the capsule.
- If possible, regulate the organisation's work so that pregnant healthcare professionals and those planning to become pregnant do not handle these medications.
- The tools (e.g. mortar, tablet crusher) used for preparing these medications must not be used for preparing other medicines.

Table structure and use

Detailed explanations of the keywords and expressions, together with instructions for administration, are provided in the section "Definitions and preparation for administration".

The header of the table:

ATC code	Active substance	Medicinal product	Dosage form	Oral administration		Administration via FT		Place of absorption / administration	Administration with food	Remarks
				Guide	Comment	Guide	Comment			

Active substance – the name of the API; information about one API is provided by dosage forms.

Yellow box – the API is a hazardous drug.

Light yellow box – the API is hazardous to pregnant women.

Medicinal product – the names of medicinal products (medicines) with that API; the medicines registered and marketed in Estonia, and some most commonly used unregistered medicines; the table does not include registered medications that are not available in Estonia.

Dosage form – the table shows the different formulations of the same API on separate lines; if the same preparation method is used for different formulations, the following boxes are combined; if the handling of medicines from different manufacturers is different for the same pharmaceutical form, they are listed on separate lines.

Oral administration – administration of the medicine to a patient with dysphagia who has difficulty swallowing, but the medicine is administered orally or into the mouth (under the tongue or in the cheek).

Guide – a term characterizing the preparation; a more detailed description and the procedure is given in the section "Definitions and preparation for administration".

Green box – can be administered.

Red box – cannot be administered.

Comment – details on taste, mixing with food, FT diameter, risks, etc.

Administration via FT – administering the medicine to a patient with dysphagia when it is not possible to administer the medicine orally, and the medicine is administered via an enteral FT.

Place of absorption/administration – the region of the gastrointestinal tract (stomach, duodenum, small intestine etc.) where the active substance is absorbed (if known) or where the medicine can be administered (if known); unless otherwise indicated, administration to the stomach is permitted.

Administration with food – whether food affects the absorption of the medicine to a clinically relevant extent; if necessary, a specifying comment is added: before or after a meal, on an empty stomach etc.

Remarks – the required safety measures (explanations in the section "Safety during preparation"); the result of the improper handling of the formulation (e.g. accelerated release or degradation of the API), an alternative if the API cannot be administered; some side effects: difficulty swallowing, dry mouth, etc.; stability of the API.

Abbreviations and symbols used in the table:

API – active pharmaceutical ingredient
gastrores. – gastro-resistant coating
ETF – enteral tube feed
FT – feeding tube
GIT – gastrointestinal tract
modif. – modified
NaH-carb – sodium hydrogen carbonate
prolong. – prolonged-release
p/r – per rectum
SmPC – summary of product characteristics
TDM – therapeutic drug monitoring
↑ – increase
↓ – decrease
≥ – is greater than or equal to

Definitions and preparation for administration

DISPERSING – after adding liquid to the medicine, the liquid breaks it down into smaller particles, resulting in a suspension.

DILUTING – after adding liquid to the medicine, the liquid dissolves it, forming a clear, particle-free solution.

CRUSHING – the physical crushing of the medicine in a mortar or tablet crusher.

ALTERNATIVE – an alternative is provided for APIs that cannot be administered, either a specific API or formulation is suggested, or the comment "change the API".

change the API – since the API cannot be administered, another suitable API should be used

ALSO SUITABLE TO THE DUODENUM/JEJUNUM – indicates that the medicine is also suitable for administering to patients with duodenal and jejunal FTs.

AT THE SAME TIME REGARD TO FOOD – it does not matter if the medicine is taken before or after a meal. However, it is essential to ensure that medicine is taken at the same time regarding meals, e.g. always before or after a meal.

CAN MIX WITH APPLE PUREE/YOGHURT – suggests that the medicine can be mixed with food similar in consistency to fruit puree or yoghurt; see the specific instructions in the table.

CHEW or CRUSH/DISPERSE – put the tablet in the patient's mouth and ask it to be chewed before swallowing. If the patient cannot chew, the medicine can be crushed or dispersed before administration (as instructed in the table). After crushing, the medicine can be mixed with a small amount (1-2 teaspoons) of food or a liquid with suitable viscosity. After dispersing, it may be necessary to bring the resulting suspension to a suitable consistency.

CRUSH

Oral administration – crush the medicine. Add 5ml of water and grind more until a smooth paste is formed. Add 5-15ml of water and stir until smooth. Draw into a syringe or pour into a cup. Rinse the mortar/crusher with water to remove any residue and draw it into a syringe or pour into the cup. Administer the medicine. Rinse the container with another 10ml of water and administer.

Alternatively, the medicine can be mixed with a small amount of food at room temperature or a liquid with suitable viscosity after the crushing.

Administration via FT – stop the ETF and rinse the FT with 10-30ml of water. Crush the medicine. Add 5ml of water and grind until a smooth paste is formed. Add 5-15ml of water and stir until smooth. Pull into a syringe and administer via the FT. Rinse the mortar/crusher with water to make sure that any residue is removed, and draw the rinsing solution into the syringe as well. Administer the medicine via the FT. Rinse the syringe with another 10ml of water and administer it via the FT.

DILUTE

Oral administration – take the required dose; dilute the medicine with at least the same amount of water (or another permitted liquid); if necessary, bring the solution/suspension to a suitable consistency.

Administration via FT – measure the required dose; dilute the medicine with at least the same amount of water.

DISPERSE

Oral administration – place the medicine into a cup/syringe and add 10-20ml of water. Allow to disperse, gentle shaking is allowed. If necessary, bring the resulting suspension to a suitable consistency. If any medicine is left in the cup/syringe after administration, rinse with a small amount of water and administer.

Administration via FT – stop the ETF and rinse the FT with 10-30ml of water. Put the medicine in the syringe. Draw 10-20ml of water into the syringe and allow the medicine to disperse; gentle shaking is allowed. Administer via the FT. Draw another 10ml of water into the syringe and administer via the FT.

NB! If there is no 'DON'T CRUSH' indication, the medicine may be crushed if necessary.

Specification of the dispersion rate:

- below 3 minutes – no specification,
- 4-9 minutes – comment **"takes time"** in the guideline box,
- over 9 minutes – approximate time in the guideline box (e.g. 10min),
- the dispersion time varies depending on the medicine – comment **"may take time"** in the guideline box.

DISPERSE CAPSULE CONTENTS – stop the ETF and rinse the FT with 10-30ml of water. Open the capsule and sprinkle the content into the syringe. Draw 10-20ml of water into the syringe and allow to disperse, gentle shaking is allowed. Administer via the FT. Rinse the syringe with another 10ml of water and administer via the FT.

NB! If there is no indication "DON'T CRUSH", the capsule's contents may be crushed before dispersion, if necessary.

DISPERSE IN MOUTH – put the tablet in the patient's mouth and keep it there until the tablet disintegrates; the undispersed medicine should not be chewed or swallowed.

DISSOLVE

Oral administration – dissolve the medicine in a cup or syringe according to the manufacturer's instructions; if necessary, bring the resulting solution to a suitable consistency.

Administration via FT – dissolve the medicine in a syringe according to the manufacturer's instructions.

DON'T CRUSH! – crushing or chewing the medicine will change the expected effect of the medicine.

MAKE HALF –scored tablets can be split before adding water to speed up the dispersion of the medicine. If it is recommended, a note is added to the comments box.

MONITOR/TDM needed – for APIs where a change in the dosage form may have a clinically significant impact on the effect of the medicine, details on how to monitor the patient's treatment are provided.

NO – the formulation cannot be administered; choose an alternative or consult a pharmacist.

NUMBING – some APIs may have a local anaesthetic/numbing effect on the oral mucosa. This effect is generally concealed by the tablet's or capsule's coating, but this side effect may occur after crushing or dispersing the medicine and the swallowing function of the patient may deteriorate. Therefore, it is recommended to rinse the mouth after administering this medicine or to administer it at least 30 minutes before a meal.

OPEN CAPSULE – open the capsule and sprinkle the contents into the mouth, on solid food or disperse in a liquid.

Mouth – when sprinkling into the mouth, give the patient a drink afterwards.

Solid food – when sprinkling on the food, mix it with a small amount (1-2 tablespoons) of food or a liquid of suitable viscosity; these should be at room temperature.

Liquid – when dispersed in a liquid, sprinkle the capsule's contents into a cup and add 10-20ml of water, shaking gently if necessary. If there is any medicine left in the cup after administration, rinse it with a small amount of water and administer.

REGARDLESS OF FOOD – this comment is used in the comments box "administration with food", if the effect of the food on the pharmacokinetics or effect of the medicine is absent or clinically insignificant.

RINSE – this comment is added in the comments box if the syringe and/or FT used to administer the medicine should be rinsed more thoroughly than usual to prevent any loss of the medicine or the clogging of the FT.

RISK OF FT BLOCKAGE – although the risk of clogging should always be considered when administering medicine via FTs, it is emphasized explicitly for medicines where this is more likely.

SOME TABLETS ARE SCORED – this is indicated next to the tablets when there is no other way to administer the API; this suggests that the tablet may be split to facilitate swallowing, as sometimes the patient may be able to swallow the split tablet.

SPRAY SUBLINGUALLY – spray the medicine under the tongue.

SUBLINGUALLY or BUCCALLY – put the medicine under the tongue or in the buccal cavity, the medicine cannot be chewed or swallowed. Adequate saliva production is required. The risk of aspiration is increased in unconscious patients.

Saliva necessary – to draw attention to the need for saliva to dissolve/disperse and absorb tablets administered under the tongue and buccally, a note is made in the comments box for the respective medicines.

YES

Oral administration – the medicine may be administered according to the manufacturer's instructions; take the required dose; if necessary, adjust the solution/suspension to a suitable consistency.

Administration via FT – the medicines may be administered according to the manufacturer's instructions; take the required dose.

ATC code	Active substance	Medicinal product	Dosage form	Oral administration		Administration by FT		Site of absorption/ administration	Administration with food	Remarks
				Guide	Comment	Guide	Comment			
**ALIMENTARY TRACT AND METABOLISM**										
**DRUGS FOR ACID RELATED DISORDERS**										
A02AD81	**Aluminium hydroxide + magnesium hydroxide**	Maalox	oral suspension	YES [1,2,4]	thicken if necessary [4]	NO [2,4]	physical interactions with ETF; risk of FT blockage [2,3,4]	low absorption; local effect in the stomach [1,3,4]<br>not suitable to the duodenum and jejunum [4]	physical interactions with ETF [2,4]	affects the absorption of other drugs [1,4]<br>**ALTERNATIVE:** change the API [6]
			chewable tablet	CHEW [1] or CRUSH [4]	can mix with apple puree/yoghurt [4]					
A02BC01	**Omeprazole**	Gasec gastrocaps	gastro-resistant capsule (modified micropellets)	OPEN CAPSULE DON'T CRUSH! [1,4]	can mix with acidic drink/apple puree/ yoghurt [1,4,5]	NO [4,6]	risk of FT blockage [6]	the small intestine [1,3]<br>also suitable to the duodenum and jejunum [3,4]	can delay, but does not ↓ absorption [3]<br>preferably 30-60min before breakfast [4,5]	opening the capsule does not alter the release of the API [1,4]; when the capsule's contents are crushed, the API is broken down by stomach acid [1,2,3,4]<br>**ALTERNATIVE:** change the API [6]
		Omep, Omep Uno, Ultop	gastro-resistant capsule (modified pellets)							
		Omeprazole (Olainfarm, Ratiopharm, Stada)	gastro-resistant capsule (modified microgranules)							
A02BC02	**Pantoprazole**	Controloc, Nolpaza, Pantoprazol (Actavis, Mepha, Ratiopharm)	gastro-resistant tablet	NO [1,2,4]	-	NO [1,3,4]	-	the small intestine [3]	-	when the tablet is crushed, the API is broken down by stomach acid [1,2,3,4]<br>**ALTERNATIVE:** change the API [6]
A02BC03	**Lansoprazole**	Lanzostad	gastro-resistant capsule (modified granules)	OPEN CAPSULE DON'T CRUSH! [1,4,5]	can mix with apple puree/yoghurt [1,4,5]	DISPERSE CAPSULE CONTENTS DON'T CRUSH! [3,4,5]	disperse in 10ml of 8.4% NaH-carb [3,4,5]<br>shake until uniform [4]<br>FT ≥ CH8 [4,6]	the small intestine [3]	preferably 30-60min before breakfast [3,4,5]	opening the capsule does not alter the release of the API [6]; when the capsule's contents are crushed, the API is broken down by stomach acid [1,2,3,4]<br>may cause dry and sore mouth [4]
A02BC05	**Esomeprazole**	Nexium, Nexmezol, Esomeprazole Actavis	gastro-resistant tablet	DISPERSE DON'T CRUSH! [1,4,5]	can mix with apple juice/apple puree/ yoghurt [1,4,5]	DISPERSE (25-50ml) (takes time) DON'T CRUSH! [1,2,3,4,5,6]	shake until uniform [4]<br>administer in parts, shake the syringe [1,4]<br>25ml FT ≥ CH14 [5]<br>50ml FT ≥ CH8 [4,5]	the small intestine [3,4]<br>also suitable to the duodenum and jejunum [3,4]	delays and ↓ absorption, but does not ↓ the effect [1,3]<br>preferably 30-60min before breakfast [4]	dispersion of the tablet/opening of the capsule does not alter the release of the API [1,4]; when the tablet's/ capsule's contents are crushed, the API is broken down by stomach acid [1,2,3,4]<br>may cause dry mouth and taste disturbances [1,4]
		Elfimest				NO [1,6]	risk of FT blockage [6]			
		Escadra	gastro-resistant capsule (modified pellets)	OPEN CAPSULE DON'T CRUSH! [1,4,5]						
**DRUGS FOR FUNCTIONAL GASTROINTESTINAL DISORDERS**										
A03AA04	**Mebeverine**	Duspatalin	prolonged-release capsule (granules)	NO [1]	-	NO [1,3]	-	the duodenum [4]<br>not suitable to the jejunum [4]	-	the capsule's shell provides a prolonged release effect; the API is released rapidly when the capsule is opened/broken; risk of toxicity [1]<br>**ALTERNATIVE:** change the API [6]
A03AD02	**Drotaverine**	No-Spa	(film-coated) tablet	DISPERSE (takes time) [6]	-	DISPERSE (takes time) [6]	shake until uniform [6]<br>FT ≥ CH8 [6]	-	-	-
A03AX13	**Simeticone**	Cuplaton, Espumisan (L), Sab Simplex	oral drops	YES [1]	can mix with drink [1,5]	DILUTE [2]	-	not absorbed, local effect in the GIT [1]	regardless of food [1]	-
		Antiflat	chewable tablet	CHEW [1,5] or CRUSH [1]	-	NO [6]	no info, risk of FT blockage [6]			
		Espumisan	soft capsule	NO [1]		NO [1]	-			

ATC code	Active substance	Medicinal product	Dosage form	Oral administration		Administration by FT		Site of absorption/ administration	Administration with food	Remarks
				Guide	Comment	Guide	Comment			
A03BB01	**Butyl-scopolamine**	Buscopan	(coated) tablet	CRUSH [4]	can mix with apple puree/yoghurt [4]	CRUSH [3,4]	-	low absorption, local effect in the GIT [2,3]	regardless of food [4]	may cause dry mouth [4]
		Bysimin	injection solution (pH 3.7-5.5)	DILUTE [4]	-	DILUTE [4]				
A03FA01	**Metoclopramine**	Cerucal, Metoclopramide Accord	tablet	CRUSH [2,4]	can mix with apple puree/yoghurt [4]	CRUSH [2,4]	-	-	regardless of food [1,4]	-
		-	injection solution	DILUTE [2]	-	DILUTE [2,4]				
A03FA03	**Domperidon**	Domperidon Actavis	tablet	DISPERSE (takes time) [2,3,4]	-	DISPERSE (takes time) [2,3,4]	FT ≥ CH8 [3]	-	stop ETF 30min before or after [4]	may cause dry mouth [4]
**ANTIEMETICS AND ANTINAUSEANTS**										
A04AA01	**Ondansetron**	Zofran	film-coated tablet	DISPERSE (takes time) [4]	-	DISPERSE (takes time) [4]	-	the small intestine, colon and rectum [2,4]	regardless of food [1,3,4]	may reduce GIT motility and cause constipation [1,4]
		Ondansetron Accord	injection/infusion solution	DILUTE [2,4]	can mix with orange juice [4]	DILUTE [2,4]	acidic, rinse [2] preferred for jejunal administration [2]			
A04AA02	**Granisetron**	Kytril	film-coated tablet	DISPERSE (takes time) [3,4]	bitter [4] can crush and mix with apple puree/ yoghurt [4]	DISPERSE (takes time) [3,4]	shake [3] FT ≥ CH8 [3]	-	regardless of food [1,3,4]	may reduce GIT motility and cause constipation [4]
			Injection/infusion solution concentrate	DILUTE [6]	bitter [4]	DILUTE [4]	-			
A04AD12	**Aprepitant**	Emend	capsule	OPEN CAPSULE DON'T CRUSH! [2,4]	can mix with apple puree/yoghurt [4]	DISPERSE CAPSULE CONTENTS DON'T CRUSH! [2,4]	-	the small intestine [2,4] also suitable to the jejunum[4]	regardless of food [1,4]	the pharmacokinetics of the drug may be altered if the capsule's contents are crushed [4] may cause dry mouth [4]
**BILE AND LIVER THERAPY**										
A05AA02	**Ursodeoxycholic acid**	Ursosan	capsule (granules)	OPEN CAPSULE [1,3,4]	can mix with apple puree/yoghurt [3,4]	DISPERSE CAPSULE CONTENTS [3,4]	particles settle quickly, shake the syringe; risk of FT blockage [1,3,4]	mostly: the small intestine; partly: the large intestine [3,4] also suitable to the jejunum[4]	regardless of food [3,4]	-
		Ursogrix	capsule (powder)							
A05BA80	**Phospholipids from soybeans**	Essentiale Forte N	capsule (paste)	NO [1]	-	NO [1]	-	to the small intestine [1]	-	produced from soybeans [1]
**DRUGS FOR CONSTIPATION**										
A06AB02	**Bisacodyl**	Bisacodyl Grindex	gastro-resistant tablet	NO [1,2,4,5]	-	NO [1,2,3,4]	-	not absorbed; local effect in the colon [1,3,4,5]	-	when the tablet's coating is crushed, stomach and duodenum irritation may occur; the effect does not change [2,5] **ALTERNATIVE:** p/r bisacodyl [6]
A06AB80	**Sodium picosulfate**	Guttalax, Regulax Picosulfat	oral drops	YES [1,3,4]	can dilute [1,3,4]	DILUTE [3,4]	-	not absorbed; local effect in the colon [1,3,4,5]	regardless of food [3]	may affect the absorption of other drugs; administer 1h apart [4]
A06AD11	**Lactulose**	Duphalac, Lactulose (Ratiopharm)	oral solution	YES [1,3,4]	can mix with water, juice or milk [1,3,4,5] thicken if necessary [4]	DILUTE (2-3x) [3,4]	-	not absorbed; local effect in the colon [1,3,4,5] also suitable to the duodenum and jejunum [4]	regardless of food [1,3,4]	-
		Lactulose MIP	syrup							

ATC code	Active substance	Medicinal product	Dosage form	Oral administration		Administration by FT		Site of absorption/ administration	Administration with food	Remarks
				Guide	Comment	Guide	Comment			
A06AD15	**Macrogol**	Forlax	powder for oral solution	YES<br>(look at SmPC) [1,4]	thicken if necessary [4]	YES<br>(look at SmPC) [4]	-	not absorbed; local effect in the colon [3,4]<br>also suitable to the duodenum and jejunum [4]	regardless of food [1,3,4]	affects the absorption of drugs; administer 1h apart [4]
A06AH03	**Naloxegol**	Moventig	film-coated tablet	CRUSH [1,5]	do not chew [5]<br>mix with 60-120ml water [1,5]	CRUSH [1,5]	first, mix with 60ml of water, then rinse with 60ml water [5]<br>FT ≥ CH8 [1]	-	stop ETF 0.5-1h before and 2h after [1,5]	whole and crushed tablets are bioequivalent [1]
**ANTIDIARRHEALS, INTESTINAL ANTIINFLAMMATORY/ANTIINFECTIVE AGENTS**										
A07AA11	**Rifaximin**	Colidur, Normix, Rifacol	(film-coated/coated) tablet	CRUSH [4]	very bitter [4]<br>can mix with apple puree/yoghurt [4]	CRUSH [4]	-	low absorption, local effect in the GIT [3,4,5]	regardless of food [4,5]	the resulting suspension is orange and may stain tools [4]
A07AA12	**Fidaxomicin**	Dificlir	film-coated tablet	CRUSH [2,4]	thicken if necessary [4]<br>can mix with apple puree/yoghurt [2,4]	CRUSH [2,4]	-	low absorption, local effect in the GIT [4]	regardless of food [4,5]	-
A07DA03	**Loperamide**	Imodium Instant	orodispersible tablet	DISPERSE IN MOUTH [1]	-	DISPERSE [4]	-	absorbed to the intestinal wall and acts there [3]	regardless of food [3,4]	may cause dry mouth and taste disturbances [4]
		Imodium, Loperamid Grindex	capsule (powder)	OPEN CAPSULE [4]	can mix with apple puree/yoghurt [4]	DISPERSE CAPSULE CONTENTS [4]	min 2.5ml [4]			
		Lopacut, Loperamid-Ratiopharm	film-coated tablet	CRUSH [4]		CRUSH [4]	-			
A07EA06	**Budesonide**	Entocort	prolonged-release capsule (modified granules)	OPEN CAPSULE<br>DON'T CRUSH! [4,5]	can mix with apple/ orange juice [4]<br>thicken if necessary [4]<br>with soft food [5], but not with yoghurt [4]	DISPERSE CAPSULE CONTENTS<br>DON'T CRUSH! [4]	risk of FT blockage [4]	the small and large intestine; local effect [3,4]	30min before a meal [2,4]	the API is released in the intestine [4]<br>opening the capsule does not alter the release of the API [1,4]; if the tablet's/ capsule's contents are crushed, the release of the API is not delayed until it reaches the intestine, and the prolonged effect is lost; risk of ↓ effect [6]
		Cortiment	prolonged-release tablet	NO [1,4,5]	-	NO [1,3,4]	-			
A07EC01	**Sulfasalazine**	Salazopyrin EN	gastro-resistant tablet	NO [1,2,4,5]	-	NO [1,2,3,4]	-	not absorbed; local effect in the colon [3,4]	-	in the treatment of arthritis, crushing the tablet is unlikely to alter the effect; in inflammatory bowel disease, the change may be clinically significant [6]<br>**ALTERNATIVE:** in case of inflammatory bowel disease, p/r mesalazine [6]
A07EC02	**Mesalazine**	Pentasa	prolonged-release tablet	DISPERSE (50ml)<br>DON'T CRUSH! [4]	-	DISPERSE (50ml)<br>DON'T CRUSH! [3,4,7]	particles settle quickly, shake the syringe [6]<br>FT ≥ CH14 [6]	the small intestine; but is designed to have a local effect at the end of the small and in the large intestine [3,4]<br>also suitable to the duodenum and jejunum [4]	regardless of food [3,4,5]	the different formulations may not be therapeutically equivalent [4]<br>crushing the drugs may ↓ the effect since the API is absorbed prematurely and does not reach the lower part of the intestine [6]<br>**ALTERNATIVE:** p/r mesalazine [6]
			prolonged-release granules			NO [2,3,4]	the granules remain floating on the water [4]<br>risk of FT blockage [4]			
		Asacol, Yaldigo	gastro-resistant tablet	NO [1,4,5]		NO [1,3,4]	-			
A07XA04	**Racecadotril**	Hidrasec	granules for oral suspension	YES<br>(look at SmPC) [1]	can mix with food [1,6]	NO [6]	no info, risk of FT blockage [6]	-	regardless of food [1]	**-**
			capsule (powder)	OPEN CAPSULE [6]		DISPERSE CAPSULE CONTENTS<br>(in a syringe!) [6]	powder is water repellent, shake when administering; rinse [6]<br>FT ≥ CH10 [6]			

ATC code	Active substance	Medicinal product	Dosage form	Oral administration		Administration by FT		Site of absorption/ administration	Administration with food	Remarks
				Guide	Comment	Guide	Comment			
**DIGESTIVES, INCL. ENZYMES**										
A09AA02	**Lipase + amylase + protease**	Kreon, Panzynorm	gastro-resistant capsule (modif. gran.)	OPEN CAPSULE DON'T CRUSH! [1]	can mix with apple/ orange juice/yoghurt[1,4]	to stomach: DISPERSE DON'T CRUSH! [4]<br>to small intestine: CRUSH [4]	risk of FT blockage [2,4] FT ≥ CH10 [2,4]<br>mix with 20ml of 8.4% NaH-carb [4] consider ↑ the dose [4]	not absorbed; local effect [1] also suitable to the duodenum and jejunum [4]	with or immediately after a meal as it has a direct effect on food [3,4]	the coating of the tablet (including film-coated tablets) and of the granules/ mini-tablets in the capsule do not allow the API to be released in the stomach [1] opening the capsule does not alter the effect of the drug [6]; if the tablet's/capsule's contents are crushed, the enzymes will inactivate in the stomach acid [1,4]
		Pangrol	gastro-resistant capsule (modif. minitab.)		if the patient can swallow mini-tablets [6]	NO [1,2]	-			
		Mezym	gastro-resistant tablet	NO [1,2]	-					
		Panzynorm Forte N	film-coated tablet							
**DRUGS USED IN DIABETES**										
A10BA02	**Metformin**	Metfogamma, Metforal, Metformin (Ratiopharm, Sandoz, Teva)	film-coated tablet	CRUSH [4]	can mix with apple puree/yoghurt [4]	CRUSH [3,4]	FT ≥ CH8 [3]	mostly: the small intestine[2,4] also suitable to the duodenum but not to the jejunum (bioavailability may ↓) [2,4]	absorption may ↓ [3,4] with food GIT side effects ↓ [3,4,5]	**MONITOR** blood sugar [2,3,4]
A10BB07	**Glipiside**	Glibense GITS	prolonged-release tablet	NO [1,5]	-	NO [1]	-	-	-	if the tablet is crushed, the API is released faster; risk of toxicity [6] **ALTERNATIVE:** change the API [6]
A10BB09	**Gliclazide**	Diaprel	tablet	DISPERSE (takes time) [4]	can crush and mix with apple puree/ yoghurt [4]	DISPERSE (takes time) [4]	-	-	regardless of food [3] to prevent hypoglycaemia, administer with food [4,5]	an 80mg tablet is equivalent to a 30mg modified tablet [4]; when switching from a modified tablet, a drug-free period of a few days may be required to avoid additive effects of the two drugs [1] if the modified tablet is crushed, the API is released faster; risk of toxicity [6] **MONITOR** blood sugar [2,3,4]
		Diaprel MR, Gliclada, Gliclazide (Actavis, KRKA)	modified-release tablet	NO [1,2,4,5]	some tablets are scored [1]	NO [1,2,3,4]				
A10BB12	**Glimepiride**	Amaryl, Glimepiride Accord, Oltar	tablet	DISPERSE [4]	can crush and mix with apple puree/ yoghurt [4]	DISPERSE [4]	-	-	regardless of food [3] to prevent hypoglycaemia, administer with food [3,4,5]	**MONITOR** blood sugar [2,3,4] may cause taste disturbances and loss [4]
A10BG03	**Pioglitazone**	Pioglitazone (Accord)	tablet	CRUSH [2,4]	can mix with apple puree/yoghurt [4]	CRUSH [2,4]	-	-	regardless of food [4,5]	**MONITOR** blood sugar [2,4]
A10BH01	**Sitagliptin**	Januvia	film-coated tablet	DISPERSE [2,4]	bad taste [2] can crush and mix with apple puree/ yoghurt [4]	DISPERSE [2,4]	-	-	regardless of food [4,5]	**MONITOR** blood sugar [2,3,4]
A10BH02	**Vildagliptin**	Dalmevin, Galvus, Glypvilo, Saxotin	film-coated tablet	CRUSH [4]	can mix with apple puree/yoghurt [4]	CRUSH [4]	-	-	regardless of food [3,4]	**MONITOR** blood sugar [4]
A10BH03	**Saxagliptin**	Onglyza	film-coated tablet	CRUSH [4]	can mix with apple puree/yoghurt [4]	CRUSH [4]	-	-	regardless of food [4,5]	Onglyza tablets are difficult to break! [4] **MONITOR** blood sugar [4]

ATC code	Active substance	Medicinal product	Dosage form	Oral administration		Administration by FT		Site of absorption/ administration	Administration with food	Remarks
				Guide	Comment	Guide	Comment			
A10BH05	**Linagliptin**	Trajenta	film-coated tablet	CRUSH [4]	can mix with apple puree/yoghurt [4]	CRUSH [4]	check for large particles [4]	-	regardless of food [4,5]	Trajenta tablets are difficult to break! [4]<br>**MONITOR** blood sugar [4]
A10BK01	**Dapagliflozin**	Forxiga	film-coated tablet	CRUSH [4]	can mix with apple puree/yoghurt [4]	CRUSH [4]	-	-	regardless of food [4,5]	**MONITOR** blood sugar [4]
A10BK03	**Empagliflozin**	Jardiance	film-coated tablet	CRUSH [4]	can mix with apple puree/yoghurt [4]	CRUSH [4]	check for large particles [4]	-	regardless of food [4,5]	Jardiance tablets are difficult to break! [4]<br>**MONITOR** blood sugar [4]
A10BK04	**Ertugliflozin**	Steglatro	film-coated tablet	CRUSH [4]	can mix with apple puree/yoghurt [4]	CRUSH [4]	-	-	regardless of food [4,5]	**MONITOR** blood sugar [4]
**VITAMINS AND MINERAL SUPPLEMENTS**										
A11CA01	**Retinol (Vitamin A)**	many, some food supplements	oral drops	YES [1,4]	-	DILUTE [4]	-	the duodenum and jejunum[4]	-	light-sensitive, administer immediately! [4]
A11CC01	**Ergocalciferol (Vitamin D2)**	many, some food supplements	oral drops	YES [1]	-	DILUTE [6]	-	the small intestine; needs bile [5]	regardless of food [5]	-
A11CC04	**Calcitriol (Vitamin D)**	Rocaltrol	soft capsule	NO [1]	-	NO [1]	-	the small intestine [4]	-	if necessary, consult with a pharmacist [6]
A11CC05	**Colecalciferol (Vitamin D3)**	many, some food supplements	oral drops	YES [1,4]	can dilute [4]<br>can mix with apple puree/yoghurt [4]	DILUTE [4]	do not mix with warm water [4]	the small intestine; needs bile [5]	regardless of food [4,5]	-
A11HA03	**Tocopherol (Vitamin E)**	many, some food supplements	oral drops	YES [1]	-	DILUTE [3]	-	needs bile/pancreatic enzymes; through the lymphatic system [3,5]<br>also suitable to the jejunum[3]	regardless of food [3,5]	-
A12AA04	**Calcium carbonate**	Calcigran SINE	chewable tablet	CHEW [1,4,5]	can crush and mix with apple puree/ yoghurt [4]	CRUSH [6]	in case of jejunal FT, risk of FT blockage [6]	mostly: the duodenum [4,5],<br>partly: the jejunum [3,4]<br>needs vitamin D [5]<br>also suitable to the jejunum[3]	food can ↑ absorption [4]<br>binds phosphates [3,4]	-
A12AX80	**Calcium carbonate + colecalciferol**	Calcigran (FORTE)	chewable tablet	CHEW [1]	can crush and mix with apple puree/ yoghurt [6]	CRUSH [6]	in case of jejunal FT, risk of FT blockage [6]	see calcium carbonate and colecalciferol		

ATC code	Active substance	Medicinal product	Dosage form	Oral administration		Administration by FT		Site of absorption/ administration	Administration with food	Remarks
				Guide	Comment	Guide	Comment			
A12BA01	**Potassium chloride (KCl)**	Kaldyum, Kalinor Retard P	prolonged-release capsule	OPEN CAPSULE [5]	can mix with food [5]	NO [1,2,3,4]	the granules in the capsule get stuck in the FT [6]	the upper GIT [5]<br>the risk of GIT side effects ↑ when administered to the jejunum [3]	risk of physical interaction with food [3]<br>with food GIT side effects ↓ [4,5]	risk of GIT irritation in case of too concentrated solutions [3,4,5]<br>opening the prolong. capsule does not alter the effect of the drug [6]; if the prolong. tablet's/capsule's contents are crushed, the API is released faster; risk of GIT irritation [6]<br>**MONITOR** the potassium level in the blood [4]<br>**NB!** do not mistakenly inject the solution into a vein! [6]
		Potassium chloride (K-TAB, USP)	prolonged-release tablet	NO [2,4,5]	-		-			
		Potassium Chloride Braun 7.45%	injection solution	DILUTE (1:2) [6]	bitter [6]<br>can mix with food [6]	DILUTE (1:2) [6]	for administration to the small intestine, dilute at least to 50-100ml [3]			
A12BA80	**KCl + potassium hydrogen carbonate**	Sando K	effervescent tablet	DISPERSE (120ml) [1,2,4]	can disperse with 5ml of water and mix with 120ml of food [4]	DISPERSE (in cup; 120ml) [2,3,4]	-			
A12CA01	**Sodium chloride**		injection solution	DILUTE [6]	-	DILUTE [6]	-	-	-	should be used in patients with hyponatraemia [6]
A12CC04	**Magnesium citrate**	Magnesium Diasporal	powder/granules for oral solution	YES (look at SmPC) [1]	-	YES (look at SmPC) [6]	-	the small intestine; as chloride; needs stomach acid [2]	regardless of food [1]	-
A12CX83	**Phosphate**	Phosphate Sandoz	effervescent tablet	DISPERSE [1,5]	-	DISPERSE (in cup) [3]	min 2ml [3]<br>FT ≥ CH6 [3]	to the upper GIT [5]	regardless of food [3]	-
**BLOOD AND BLOOD FORMING ORGANS**										
**ANTITHROMBOTIC AGENTS**										
B01AA03	**Warfarin**	Marevan (Forte)	tablet	DISPERSE (takes time) [2,4]	can crush and mix with apple puree/ yoghurt [4]	DISPERSE (takes time) [2,3,4]	FT ≥ CH8 [3]	no exact info [2,3]<br>also suitable to the duodenum and jejunum [4]	ETF alters; give at the same time regard to ETF [4]	**HAZARDOUS TO PREGNANT!** [7]<br>whole and crushed tablets may not be bioequivalent [2]<br>**MONITOR** INR [2,4]
B01AC04	**Clopidogrel**	Clopidogrel (Accord, KRKA, Sanoswiss), Plavix, Zyllt, Trombex	film-coated tablet	CRUSH [2,4]	cannot be chewed; risk of mucosal damage [4]<br>can mix with apple puree/yoghurt [4]	CRUSH [2,3,4]	risk of FT blockage [2,3,4]<br>bioavailability can ↑ or ↓ [4]<br>FT ≥ CH10 [3]	-	regardless of food [1,3,4,5]	300mg tablets are hard to break [4]
B01AC06	**Acetylsalicylic acid (ASA)**	HerzAss Ratiopharm	tablet	DISPERSE [1,2,4]	can crush and mix with apple puree/ yoghurt [4,6]	DISPERSE [4]	-	mainly: the stomach and upper small intestine [3,4,5]<br>also suitable to the jejunum[4]	regardless of food [4]<br>with food GIT side effects ↓ [2,4,5]	sensitive to moisture; decomposes to salicylic acid, which is irritating to the GIT; administer immediately! [4]<br>the gastro-resistant coating is designed to protect the GIT [6]
		Alka Seltzer, Asprovit	effervescent tablet			DISPERSE (in cup) [2,3,4]				
		ASA Grindex, Aspirin Bayer, HerzASS	film-coated tablet	CRUSH [4]		CRUSH [4]				
		ASA KRKA, Aspirin Cardio, ThromboAss	gastro-resistant tablet	NO [1,2,4]		NO [1,2,3,4]				
B01AC80	**ASA + magnesium oxide**	Hjertemagnyl	film-coated tablet	CRUSH [6]		CRUSH [6]				
B01AC24	**Ticagrelor**	Brilique	film-coated tablet	CRUSH [1,2,4,5]	mix with 1/2 glass of water [1]<br>can mix with apple puree/yoghurt [4]	CRUSH [1,2,4,5]	mix with 50ml of water [1,4]<br>FT ≥ CH8 [1,2,4,5]	-	regardless of food [4,5]	absorption of the crushed tablet is faster, but the bioavailability is the same [4,5]

ATC code	Active substance	Medicinal product	Dosage form	Oral administration		Administration by FT		Site of absorption/ administration	Administration with food	Remarks
				Guide	Comment	Guide	Comment			
B01AC27	**Selexipag**	Uptravi	film-coated tablet	DISPERSE [4]	can crush and mix with apple puree/ yoghurt [4]	DISPERSE [4]	-	suitable for administration by gastric FT [4]	regardless of food [4,5]<br>with food GIT side effects ↓ [1,4,5]	-
B01AE07	**Dabigatran**	Pradaxa	capsule (pellets)	NO [1,2,4,5]	-	NO [1,2,4,5]	-	-	-	the capsule MUST NOT be opened; capsule opening ↑ bioavailability (75%) and risk of bleeding! [4,5]<br>**ALTERNATIVE:** change the API [6]
B01AF01	**Rivaroxaban**	Xarelto	film-coated tablet	CRUSH [1,2,4,5]	can mix with apple puree [1,4,5]	CRUSH [1,2,4,5]	mix with 50ml of water [1,2,4,5]	the stomach [1,4]<br>only suitable to the stomach [1,4,5]<br>not suitable to the small intestine (bioavailability ↓) [1,4,5]	up to 10mg, food has no effect; higher doses with food [1,4,5]	the bioavailability of the crushed and whole tablet is equivalent [1,4]<br>may cause dry mouth [4]
B01AF02	**Apixaban**	Eliquis	film-coated tablet	CRUSH [1,2,4,5]	can mix with glucose solution/apple juice/ puree [1,4,5]	CRUSH [1,2,4,5]	mix with 60ml of water [1,2,4,5]	the stomach [1,4]<br>only suitable to the stomach [1,2,4]<br>not suitable to the small intestine (bioavailability ↓) [1,4]	regardless of food [4,5]	the bioavailability of the crushed and whole tablet is equivalent [1,4]
B01AF03	**Edoxaban**	Lixiana	film-coated tablet	CRUSH [1,5]	can mix with apple puree [1,5]	CRUSH [1,5]	mix with 60-90ml of water [1,5]	probably the stomach [1,6]<br>only suitable to the stomach [5]	regardless of food [1,5]	the bioavailability of the crushed and whole tablet is equivalent [1]
**ANTIHEMORRHAGICS**										
B02AA02	**Tranexamic acid**	Cyclokapron	film-coated tablet	DISPERSE (takes time) [4]	can crush and mix with apple puree/ yoghurt [4]	DISPERSE (takes time) [4]	-	also suitable to the jejunum[4]	regardless of food [4,5]	-
			injection solution (pH 6.5-8)	YES [4]	≤ 100mg/ml solution undiluted [4]	DILUTE [4]				
B02BA01	**Phytomenadione**	Konakion MM Paediatric	injection and oral solution	YES [1,4,5]	can dilute with drinks [5]	YES [2,4]	-	needs bile [5]<br>to the jejunum absorption ↓[4]	regardless of food [4,5]	-
**ANTIANAEMIC PREPARATIONS**										
B03AA07	**Ferrous sulphate**	Retafer	prolonged-release tablet	NO [1,4,5]	cannot be chewed/sucked [4]	NO [1,4]	-	the duodenum and upper jejunum [3,5]<br>not suitable to the jejunum [3,4]	food ↓ absorption because it ↑ the pH of the GIT [4,5]<br>with food GIT side effects ↓ [3,4,5]	1mg of iron is equivalent with<br>3mg iron fumarate<br>3mg of ferrous sulphate,<br>5mg ferrous sulphate (liquid),<br>3.7mg iron polymaltose [4]<br>risk of mucosal irritation and staining of teeth when crushing the prolong. tablet [6]
B03AB80	**Iron(III)-hydroxide polymaltose complex**	Ferrum Lek	syrup	YES [1]	can mix with juice [1,4]<br>use a straw to prevent staining of teeth [1,4]<br>not suitable if cannot swallow fluids [6]	DILUTE [4]	the syrup may stain the FT [4]			
			chewable tablet	CHEW or CRUSH [1]	-	CRUSH [6]	check for large particles [6]			
B03BB01	**Folic acid**	Folic Acid Vitabalans	tablet	DISPERSE (takes time) [2,4]	can crush and mix with apple puree/ yoghurt [4]	DISPERSE (takes time) [2,3,4]	-	the duodenum [1,3,4]<br>suitable to the duodenum, but not to the jejunum [4]	regardless of food [3,5]	-

ATC code	Active substance	Medicinal product	Dosage form	Oral administration		Administration by FT		Site of absorption/ administration	Administration with food	Remarks
				Guide	Comment	Guide	Comment			
**CARDIOVASCULAR SYSTEM**										
**CARDIAC THERAPY**										
C01AA05	**Digoxin**	Digoxin (Grindex, Nycomed)	tablet	CRUSH [2,4]	can mix with apple puree/yoghurt [4]	CRUSH [2,4]	-	the stomach and duodenum [2,3,4]<br>to the jejunum absorption ↓[2]	high-fibre food ↓ absorption [4,5]<br>administer at the same time regard to food [4,5]	**TDM needed** [4]
C01BB02	**Mexiletine**	Mexiletine hydrochloride capsules USP	capsule (powder)	OPEN CAPSULE [2]	unpleasant taste and numbing [2]	DISPERSE CAPSULE CONTENTS [2]	-	the upper small intestine [3]	regardless of food [1,3]	-
C01BC03	**Propafenone**	Propafenone Accord, Propranorm, Rytmonorm	film-coated tablet	CRUSH [2]	bitter and numbing [2]<br>use glucose solution [2]	CRUSH [2]	-	-	regardless of food [1]	-
C01BC04	**Flecainide**	Tambocor, Flecainide acetate	tablet	DISPERSE [2,4]	numbing [2,4]<br>can crush and mix with apple puree/ yoghurt [4]	DISPERSE [3,4]	FT ≥ CH8 [3]	-	regardless of food [3,4]	may cause dry mouth and taste disturbances [4]
C01BD01	**Amiodarone**	Amiokordin, Cordarone	tablet	CRUSH [2,4]	bitter; can mix with juice [2]<br>can mix with apple puree/yoghurt [3,4]	CRUSH [2,3,4]	bioavailability may ↓ by FT administration [4]	absorption slow [1,5]<br>to the duodenum and jejunum [2]	food ↑ absorption;<br>administer at the same time regard to food [4,5]	due to its long half-life, treatment may be discontinued for 2 weeks [3,4]<br>may cause taste disturbances[4]
C01BD07	**Dronedarone**	Multaq	film-coated tablet	NO [6]	no info [6]	NO [6]	no info [6]	-	-	**ALTERNATIVE:** change the API [6]
C01DA02	**Glyceryl trinitrate**	Nitroglycerin Nycomed	sublingual tablet	SUBLINGUALLY or BUCCALLY [1,4]	saliva necessary [2]	NO [4,6]	can be administered under the tongue/in the cheek [2,4,6]<br>risk of aspiration [4,6]	the oral mucosa [1,3,4]<br>not suitable to the stomach/ intestine (bioavailability ↓) [3,4]	regardless of food [4]	the effect is lost when the sublingual tablet/ spray is given by FT [6]<br>**ALTERNATIVE:** change the API [6]
		Nitromint Spray	sublingual spray	SPRAY SUBLINGUALLY [1,4]	-	NO [4,6]	can be sprayed under the tongue [4,6]			
C01DA08	**Isosorbide dinitrate**	ISDN-Ratiopharm	tablet	SUBLINGUALLY or BUCCALLY [1]	can be chewed but not swallowed [1]<br>saliva necessary [2]	NO [6]	can be administered under the tongue/in the cheek [2,6]<br>risk of aspiration [6]	whole GIT [1,3,4]<br>in the mouth (sublingually) the fastest [1,3,4]	regardless of food [3,4]	the effect is lost when the sublingual tablet/spray is given by FT [6]<br>if the prolong. capsule's contents are crushed, the API is released faster; risk of toxicity [6]<br>**ALTERNATIVE:** change the API [6]
		Isocet spray	sublingual spray	SPRAY SUBLINGUALLY [1,4]	-	NO [6]	can be sprayed under the tongue [4,6]			
		ISDN-Ratiopharm	prolonged-release capsule	NO [1,4,5]		NO [1,3,4]	-			
C01DA14	**Isosorbide mononitrate**	Isosorbide mononitrate Vitabalans, Monosan	tablet	CRUSH [3]	-	CRUSH [3]	FT ≥ CH8 [3]	-	regardless of food [3,4]	bioavailability may ↑ when the (short-acting) tablet is crushed [2]<br>opening the prolong. capsule does not alter the effect of the drug [6]; if the prolong. tablet's/capsule's contents are crushed, the API is released faster; risk of toxicity [6]
		Olicard	prolonged-release capsule (modified spheres)	OPEN CAPSULE DON'T CRUSH! [2,6]		NO [1,2,3,4,6]	risk of FT blockage [6]			
		Imdur, Isomonit	prolonged-release tablet	NO [1,2,4,5]	some tablets are scored [1]		-			
C01EB15	**Trimetazidine**	Preductal MR, Trimetazidine (Rivopharm, Teva)	prolonged-release tablet	NO [1]	-	NO [1]	-	-	-	if the tablet is crushed, the API is released faster; risk of toxicity [6]<br>**ALTERNATIVE:** change the API [6]

ATC code	Active substance	Medicinal product	Dosage form	Oral administration		Administration by FT		Site of absorption/ administration	Administration with food	Remarks
				Guide	Comment	Guide	Comment			
C01EB17	**Ivabradine**	Brediwal, Ivabradine (Accord, Grindex, Mylan), Procorlan	film-coated tablet	CRUSH [2,4]	can mix with apple puree/yoghurt [2,4]	CRUSH [2,4]	-	absorption is not altered by FT administration [4]	with food [1,4]	-
C01EB18	**Ranolazine**	Ranexa	prolonged-release tablet	NO [1,5]	-	NO [1,3]	-	-	-	if the tablet is crushed, the API is released faster, risk of toxicity [6] **ALTERNATIVE:** change the API [6]
**ANTIHYPERTENSIVES**										
C02AC05	**Moxonidine**	Moxogamma, Moxonidin (Actavis, Hexal), Physiotens, Stadapress	film-coated tablet	DISPERSE (takes time) [2,4]	can crush and mix with apple puree/ yoghurt [2,4]	DISPERSE (takes time) [2,3,4]	min 2ml [4] FT ≥ CH8 [3]	-	regardless of food [1,3,4]	may cause dry mouth [4]
C02CA04	**Doxazosin**	Cardura, Kamiren	tablet	DISPERSE [2]	-	DISPERSE [2,3]	FT ≥ CH8 [3]	-	regardless of food [3]	if the prolong. tablet is crushed, the API is released faster; risk of toxicity [6] disperse only in water for injections; precipitates due to chloride ions [2,3]
		Cardura XL, Doxalfa, Kamiren XL	prolonged-release tablet	NO [1,2,5]		NO [1,2,3]				
C02KX01	**Bosentan**	Bosentan (Accord, Norameda)	film-coated tablet	DISPERSE [4]	can crush and mix with apple puree/ yoghurt [4]	DISPERSE [4]	min 5ml [4]	-	regardless of food [4,5]	**HAZARDOUS TO PREGNANT!** [1,7]
C02KX02	**Ambrisentan**	Ambrisentan (Mylan, Normeda), Volibris	film-coated tablet	DISPERSE [4]	can crush and mix with apple puree/ yoghurt [4]	DISPERSE [4]	-	-	regardless of food [1,4,5]	**HAZARDOUS TO PREGNANT!** [1,7]
C02KX05	**Riociguat**	Adempas	film-coated tablet	CRUSH [1,4,5]	can mix with apple puree/yoghurt [1,4,5]	CRUSH [4]	-	-	administer at the same time regard to food [1,4,5]	-
**DIURETICS**										
C03AA03	**Hydrochloro-thiazide**	Hypothiazid (25mg)	tablet	DISPERSE (takes time) [4]	can crush and mix with apple puree/ yoghurt [4]	DISPERSE (takes time) [4]	-	also suitable to the duodenum and jejunum [4]	regardless of food [4,5]	-
C03BA11	**Indapamide**	Tertensif	film-coated tablet	CRUSH [4]	can mix with apple puree/yoghurt [4]	CRUSH [4]	-	not suitable to the jejunum (absorption ↓) [2]	regardless of food [3,4,5] with food GIT side effects ↓ [5]	if the prolong. tablet is crushed, the API is released faster, risk of toxicity [6] may cause dry mouth [4]
		ProIndap	capsule	OPEN CAPSULE [6]		DISPERSE CAPSULE CONTENTS [6]	FT ≥ CH8 [6]			
		Indamax, Rawel SR, Tertensif SR	prolonged-release tablet	NO [1]	-	NO [1]	-			
C03CA01	**Furosemide**	Furosemid (Nycomed, Teva)	tablet	DISPERSE [4]	can mix with apple puree/yoghurt [4]	DISPERSE [4]	-	the small intestine [3] also suitable to the duodenum and jejunum [3,4]	stop ETF 30min before and after a meal [4]	the tablet can be administered sublingually [5] opening the prolong. capsule does not alter the effect of the drug [6]; if the capsule's contents are crushed, the API is released faster; risk of toxicity [6]
				CRUSH (500mg tablet) [4]		CRUSH (500mg tablet) [4]				
		Lasix retard	prolonged-release capsule (modified granules)	OPEN CAPSULE; DON'T CRUSH! [6]		NO [1]	the granules in the capsule stick to the wall of the FT [6]			
C03CA04	**Torasemide**	Torasemide (Hexal, Teva), Trifas	tablet	CRUSH [2]	-	CRUSH [2]	rinse [2]	-	regardless of food [1]	-

ATC code	Active substance	Medicinal product	Dosage form	Oral administration		Administration by FT		Site of absorption/ administration	Administration with food	Remarks
				Guide	Comment	Guide	Comment			
C03DA01	**Spironolactone**	Spirix	tablet	CHEW or CRUSH [1,2,4]	can mix with apple puree/yoghurt [4]	CRUSH [2,3,4]	the suspension may thicken, risk of FT blockage [2]<br>FT ≥ CH8 [3]	no exact info [6]<br>also absorbs in the mouth [3]<br>also suitable to the duodenum and jejunum [4]	regardless of food [4,5], administer at the same time regard to food [5]	-
		Spironolactone Accord	film-coated tablet	CRUSH [2,4]						
**PERIPHERAL VASODILATORS**										
C04AD03	**Pentoxifylline**	Pentilin, Vasonit retard	prolonged-release tablet	NO [1,2,4,5]	some tablets are scored [1]	NO [1,2,4]	-	-	-	if the tablet is crushed, the API is released faster; risk of toxicity [6]<br>**ALTERNATIVE:** change the API [6]
C04AX21	**Naftidrofuryl**	Enelbin retard	prolonged-release tablet	NO [1]	-	NO [1]	-	-	-	if the tablet is crushed, the API is released faster, risk of toxicity [6]<br>the API may damage the mucosa [2,3]<br>**ALTERNATIVE:** change the API [6]
**BETA BLOCKING AGENTS**										
C07AA05	**Propranolol**	Propranolol Accord, Propra-Ratiopharm	film-coated tablet	CRUSH [4]	can mix with apple puree/yoghurt [4]	CRUSH [4]	-	mostly: the large intestine [2]<br>also suitable to the duodenum and jejunum [4]	administer at the same time regard to food [2]	-
C07AA07	**Sotalol**	Sotahexal	tablet	CRUSH [2,4]	can mix with apple puree/yoghurt [4]	CRUSH [2,3,4]	-	the whole GIT [2]<br>also suitable to the jejunum[2]	food ↓ absorption, particularly dairy products [1,4,5]<br>stop ETF 30min before and after [4]	may cause taste disturbances [4]
C07AB02	**Metoprolol**	Metoprolol (Stada, Ratiopharm)	tablet	CRUSH [2,4]	can mix with apple puree/yoghurt [4]	CRUSH [2,3,4]	-	also suitable to the duodenum and jejunum [4]	administer at the same time regard to food [4]	dispersion of the prolong. tablet does not alter the release of the API [6]; if the tablet is crushed, the API is released faster, risk of toxicity [6]<br>may cause dry mouth [4]
		Betaloc Zok, BloxaZoc, MetoZok	prolonged-release tablet	DISPERSE<br>DON'T CRUSH! [6]	some tablets are scored [1]	DISPERSE<br>DON'T CRUSH! [6]				
		Metoprolol (Polpharma, Ratiopharm retard)		NO [1,2,4,5]		NO [1,2,3,4]				
C07AB03	**Atenolol**	Atenolool Ratiopharm	film-coated tablet	DISPERSE<br>(may take time) [2,4]	bitter [4]<br>can crush and mix with yoghurt [4]	DISPERSE<br>(may take time) [2,3,4]	-	the jejunum and ileum [2,3]<br>also suitable to the jejunum[4]<br>absorption may ↓ when administered by FT [4]	regardless of food [4]	may cause dry mouth [4]
C07AB07	**Bisoprolol**	Bisoprolol (Accord, Vitabalans), Sombycor	(film-coated) tablet	DISPERSE [2,4]	can crush and mix with apple puree/ yoghurt [4]	DISPERSE [2,3,4]	-	-	regardless of food [1,3,4,5]	-
C07AB12	**Nebivolol**	Nebicard, Nebilet, Nebiphar, Nebivolol (Actavis, Stada)	tablet	CRUSH [2,4]	can mix with apple puree/yoghurt [4]	CRUSH [2,4]	-	-	regardless of food [1,3,4]	-
C07AG01	**Labetalol**	Trandate	film-coated tablet	DISPERSE<br>(takes time) [2,3,4]	bitter [2,3,4]<br>disperse in juice [2,3,4]<br>can crush and mix with apple puree/ yoghurt [4]	DISPERSE<br>(takes time) [3,4]	FT ≥ CH8 [3]	-	food ↑ absorption [1,4]<br>with food [1,4]	the API is rapidly degraded by oxygen; administer immediately [2]
			injection solution	DILUTE [2,3,4]	bitter taste, hide with juice [2,3,4]	DILUTE [2,3,4]	-			
C07AG02	**Carvedilol**	Carvedilol Hexal	tablet	DISPERSE<br>(may take time) [2,4]	can crush and mix with apple puree/ yoghurt [4]	DISPERSE<br>(may take time) [2,3,4]	precipitates quickly, shake [2,3,4]<br>FT ≥ CH8 [3]	better absorption in the upper intestine [3]	slower absorption [1,2,3,4]<br>with food side effects ↓ [1,2,3,4,5]	may cause dry mouth [4]

ATC code	Active substance	Medicinal product	Dosage form	Oral administration		Administration by FT		Site of absorption/ administration	Administration with food	Remarks
				Guide	Comment	Guide	Comment			
**CALCIUM CHANNEL BLOCKERS**										
C08CA01	**Amlodipine**	Amgen, Amiocard, Amlodigamma, Amlodipine (Actavis, Ratiopharm, Vitabalans), Hipres, Norvasc	tablet	DISPERSE (may take time) [2,4]	bitter [4]<br>can crush and mix with apple puree/ yoghurt [4]	DISPERSE (may take time) [2,3,4]	-	-	regardless of food [1,3,4,5]	light-sensitive, administer immediately! [2]<br>may cause dry mouth and swallowing difficulties [4]
C08CA02	**Felodipine**	Felodipin (Hexal), Plendil, Presid	prolonged-release tablet	NO [1,2,4,5]	-	NO [1,2,3,4]	-	-	-	if the tablet is crushed, the API is released faster, risk of toxicity [6]<br>**ALTERNATIVE:** change the API [6]
C08CA05	**Nifedipine**	Nifedipin-Ratiopharm	oral drops	YES [1]	-	DILUTE [6]	-	mostly: the stomach [3]<br>not suitable to the jejunum [2,3]	regardless of food [1,4]	drops are for short-term use [6]<br>light-sensitive, administer immediately! [2]<br>if the modified or prolong. tablets are crushed, the API is released faster; risk of toxicity [6]<br>may cause dry mouth [4]<br>**ALTERNATIVE:** change the API [6]
		Cordipin XL	modified-release tablet	NO [1,2,4,5]		NO [1,2,3,4]				
		Cordipin retard, Corifar (retard)	prolonged-release tablet							
C08CA06	**Nimodipine**	Nimotop	film-coated tablet	CRUSH [2,4]	can mix with apple puree/yoghurt [4]	CRUSH [2,3,4]	prefer IV administration [4]	absorption varies [3,4]	regardless of food [1,2,3]	light-sensitive, administer immediately! [2,4]
C08CA08	**Nitrendipine**	Lusopress, Nitresan	tablet	DISPERSE (10min) [6]	during preparation hide from light [6]	DISPERSE (10min) [6]	during preparation, hide from light [6]<br>FT ≥ CH8 [6]	-	with food [1]	light-sensitive, administer immediately! [1]<br>avoid if possible [6]
C08CA09	**Lacidipine**	Lacipil	film-coated tablet	CRUSH [2]	-	CRUSH [6]	risk of FT blockage [2]<br>FT ≥ CH8 [6]	-	regardless of food [1,3]	light-sensitive, administer immediately! [1]<br>avoid if possible [6]
C08CA13	**Lercanidipine**	Lercandipine Actavis, Lercapin	film-coated tablet	CRUSH [2,4]	can mix with apple puree/yoghurt [4]	CRUSH [2,4]	-	-	alters [2,3,4]<br>stop ETF 30min before and after [2,4]	-
C08DA01	**Verapamil**	Verapamil-Ratiopharm	film-coated tablet	CRUSH [2,4]	bitter [2,4] and numbing [2]<br>can mix with apple puree/yoghurt [4]	CRUSH [2,4]	-	-	regardless of food [1,3,4]	if the prolong. tablet is crushed, the API is released faster, risk of toxicity [6]<br>may cause dry mouth [4]
		Isoptin retard	prolonged-release tablet	NO [1,2,4,5]	some tablets are scored [1]	NO [1,2,3,4]				
			injection solution (pH 4-6.5)	DILUTE [2,3,4]	bitter [2] and numbing [2]	DILUTE [2,3,4]				
C08DB01	**Diltiazem**	Diltiazem (Lannacher)	prolonged-release tablet	NO [1,2,5]	-	NO [1,2,3]	-	-	-	if the tablet is crushed, the API is released faster, risk of toxicity [6]<br>may cause dry mouth [4]<br>**ALTERNATIVE:** change the API [6]
**AGENTS ACTING ON THE RENIN-ANGIOTENSIN SYSTEM**										
C09AA01	**Captopril**	Kaptopril (KRKA)	tablet	DISPERSE (may take time) [2,4]	can crush and mix with apple puree/ yoghurt [4]	DISPERSE (may take time) [2,3,4]	-	the upper small intestine [2,3]<br>to the jejunum the bioavailability may ↓ [2,3]	decreases [1,2,3,4]<br>stop ETF 0.5-1h before and after [2,4,5]	unstable in water, administer immediately [5]<br>may cause taste disturbances and mouth ulcers [4]
C09AA02	**Enalapril**	Berlipril, Enahexal, Enalapril (Vitabalans), Enap	tablet	CRUSH [2,4]	bitter [2,4]<br>can mix with apple puree/yoghurt [4]	CRUSH [2,3,4]	FT ≥ CH8 [6]	-	regardless of food [1,3,4]	may cause taste disturbances [4]

ATC code	Active substance	Medicinal product	Dosage form	Oral administration		Administration by FT		Site of absorption/ administration	Administration with food	Remarks
				Guide	Comment	Guide	Comment			
C09AA04	**Perindopril**	Prenessa, Prestarium Arginine, Stopress	(film-coated) tablet	CRUSH [2,4]	can mix with apple puree/yoghurt [4]	CRUSH [2,3,4]	-	-	-	may cause dry mouth, taste disturbances and swallowing difficulties[4]<br>avoid if possible [2,3]
C09AA05	**Ramipril**	Amipril, Cardace, Ramicor, Ramipril (Actavis)	tablet	DISPERSE (takes time) [4]	bitter [2,4]<br>can crush and mix with apple puree/ yoghurt [4,5]	DISPERSE (takes time) [3,4]	shake before administration [3,4]<br>FT ≥ CH8 [3]	-	regardless of food [1,3,4]	may cause dry mouth, taste disturbances and swallowing difficulties[4]
C09AA09	**Fosinopril**	Fosinopril Actavis, Monopril	tablet	DISPERSE (takes time) [4]	can crush and mix with apple puree/ yoghurt [4]	DISPERSE (takes time) [2,3,4]	-	the duodenum and jejunum[4]<br>also suitable to the jejunum[4]	regardless of food [1,3,4,5]	may cause dry mouth, taste disturbances, swelling of the tongue, mouth ulcers and swallowing difficulties[4]<br>avoid if possible [3]
C09AA10	**Trandolapril**	Gopten	capsule	OPEN CAPSULE [2,4]	bitter [2,4]<br>can mix with apple puree/yoghurt [4]	DISPERSE CAPSULE CONTENTS [2,4]	-	-	regardless of food [3,4]	may cause dry mouth and taste disturbances [4]<br>avoid if possible [3]
C09CA01	**Losartan**	Lorista, Lorartan (Accord), Lozap	film-coated tablet	DISPERSE [4]	bad taste [2,4]<br>can mix with apple puree/yoghurt [4]	DISPERSE [2,3,4]	FT ≥ CH8 [3]	-	regardless of food [1,3,4]	may cause dry mouth and taste disturbances [4]
C09CA03	**Valsartan**	Diovan, Valsacor, Valsartan (Medochemie), Valzap	film-coated tablet	DISPERSE [4]	can mix with apple puree/yoghurt [4]	DISPERSE [4]	shake before administration [4]	probably: the upper GIT [3]	regardless of food [1,3,4,5]	the bioavailability of the suspension is not equivalent [5] and may be higher than the tablet [4]<br>may cause dry mouth [4]
C09CA06	**Candesartan**	Atacand, Canocord, Cantar, Carzan, Prescanden	tablet	CRUSH [4]	can mix with apple puree/yoghurt [4]	CRUSH [3,4]	FT ≥ CH8 [3]	-	regardless of food [1,4,5]	-
C09CA07	**Telmisartan**	Micardis, Telmisartan (Ratiopharm, Sandoz), Tolura	tablet	CRUSH [2,4]	bitter [4]<br>can mix with apple puree/yoghurt [4]	CRUSH [2,3,4]	FT ≥ CH8 [3]	-	regardless of food [1,4,5]	sensitive to moisture, administer immediately! [1]<br>may cause dry mouth [4]
C09CA08	**Olmesartan**	Mesar, Olimestran medoxomil (Accord), Olmestra	film-coated tablet	CRUSH [4]	can mix with apple puree/yoghurt [4]	CRUSH [4]	-	probably: the stomach [3]	regardless of food [1,3,4,5]	-
**LIPID MODIFYING AGENTS**										
C10AA01	**Simvastatin**	Simvacor, Simvastatin (Ratiopharm), Vasilip	tablet	CRUSH [2,4]	can mix with apple puree/yoghurt [4]	CRUSH [2,4]	FT ≥ CH8 [3]	-	regardless of food [1,3,4,5]	light-sensitive, administer immediately! [2]
C10AA03	**Pravastatin**	Pravahexal	tablet	CRUSH [2,4]	can mix with apple puree/yoghurt [4]	CRUSH [2,4]	FT ≥ CH8 [3]	-	regardless of food [1,3,4,5]	-
C10AA04	**Fluvastatin**	Fluvastatin (Actavis), Lescol XL	prolonged-release tablet	NO [1,2,4,5]	-	NO [1,2,3,4]	-	-	-	if the tablet is crushed, the API is released faster, risk of toxicity [6]<br>**ALTERNATIVE:** change the API [6]
C10AA05	**Atorvastatin**	Atoris, Atorvastatin (Accord, Zentiva, Tad, Teva)	film-coated tablet	DISPERSE (takes time) [4]	can crush and mix with yoghurt [4]	DISPERSE (takes time) [3,4]	FT ≥ CH8 [3]	probably: the upper GIT [3]	regardless of food [1,3,4,5]	light-sensitive, administer immediately! [2,3]

ATC code	Active substance	Medicinal product	Dosage form	Oral administration		Administration by FT		Site of absorption/ administration	Administration with food	Remarks
				Guide	Comment	Guide	Comment			
C10AA07	**Rosuvastatin**	Crestor, Roscard, Rosuvastatin (Tad, Teva Pharma), Roswera	film-coated tablet	DISPERSE [4]	can crush and mix with apple puree/ yoghurt [4]	DISPERSE [4]	FT ≥ CH8 [3]	-	regardless of food [1,3,4,5]	-
C10AB05	**Fenofibrate**	Lipanthyl	capsule	OPEN CAPSULE [2]	can mix with apple puree/yoghurt [6]	DISPERSE CAPSULE CONTENTS [2]	-	-	regardless of food [1,4]	-
C10AC01	**Colestyramine**	Colestyramin (Hexal, Ratiopharm)	powder for oral suspension	YES (look at SmPC) [2,4,5]	can mix with water/ carbonated drinks/ juice/liquid food [4]	DISPERSE (in cup; 100-150ml) [2,3,4,5]	rinse [2,3,4] FT ≥ CH8 [2,3,4]	is not absorbed but binds locally with bile salts [2,3,5]	regardless of food [1,3,4]	↓ absorption of other drugs; administer 1h before or 4-6h later [2,3,4,5] may cause the staining of teeth if kept in the mouth for a long time [4,5]
C10AX09	**Ezetimibe**	Ezoleta	tablet	CRUSH [4]	can mix with apple puree/yoghurt [4]	CRUSH [3,4]	FT ≥ CH8 [3]	-	regardless of food [1,3,4,5]	may cause dry mouth [4]
**GENITOURINARY SYSTEM AND SEX HORMONES**										
G02AD06	**Misoprostol**	Angusta, Cytotec, Topogyne	tablet	DISPERSE [2,4]	can crush and mix with apple puree/ yoghurt [4]	DISPERSE [2,4]	-	-	with food GIT side effects ↓ [3]	**HAZARDOUS TO PREGNANT!** [7]
G02CB01	**Bromocriptine**	Bromocriptin Richter, Parlodel	tablet	DISPERSE [2,4]	-	DISPERSE [2,3,4]	-	-	with food GIT side effects ↓ [4,5]	light-sensitive, administer immediately [2] may cause dry mouth [4]
G02CB03	**Cabergoline**	Dostinex	tablet	CRUSH [2,4]	can mix with apple puree/yoghurt [4]	CRUSH [2,3,4]	FT ≥ CH8 [3]	-	with food GIT side effects ↓ [3,4,5]	may cause dry mouth [4]
G03DB01	**Dydrogesterone**	Duphaston	film-coated tablet	CRUSH [2]	crush in water to prevent dust [2]	CRUSH [2]	crush in water to prevent dust [2]	-	-	-
G03XA01	**Danazol**	Danol	capsule	OPEN CAPSULE [4]	can mix with apple puree/yoghurt [4]	DISPERSE CAPSULE CONTENTS [4]	-	to the jejunum absorption may ↓ [4]	regardless of food [4]	-
G04BD04	**Oxybutynin**	Driptane	tablet	CRUSH [2,4]	can mix with apple puree/yoghurt [4]	CRUSH [2,3,4]	↓ GIT's motility; not recommended for FT [4]	probably: the whole small intestine [3]	regardless of food [3,4,5]	may cause dry mouth [4]
G04BD07	**Tolterodine**	Uroflow	film-coated tablet	DISPERSE [2,4]	can crush and mix with apple puree/ yoghurt [4]	DISPERSE [2,3,4]	↓ GIT's motility; not recommended for FT [4] FT ≥ CH6 [3,4]	probably: the whole small intestine [3]	regardless of food [3,4,5]	opening the prolong. capsule does not alter the effect of the drug [6]; if the capsule's contents are crushed, the API is released faster; risk of toxicity [6] may cause dry mouth [4]
			prolonged-release capsule (minitab.)	OPEN CAPSULE DON'T CRUSH! [2]	-	NO [1,2,3]	-			
G04BD08	**Solifenacin**	Solifenacin PMCS, Tamisten	film-coated tablet	CRUSH [2,4]	can mix with apple puree/yoghurt [4]	CRUSH [2,4]	-	-	regardless of food [1,4,5]	not recommended, since the powder from the tablet irritates eyes; [2,4]

ATC code	Active substance	Medicinal product	Dosage form	Oral administration		Administration by FT		Site of absorption/ administration	Administration with food	Remarks
				Guide	Comment	Guide	Comment			
G04BE03	**Sildenafil**	Granpidam, Olvion, Revatio, Sildenafil (Accord, Actavis, Ratiopharm, Sandoz, Teva pharma), Silungo	(film-coated) tablet	CRUSH [2,4]	bad taste [4]<br>can mix with apple puree/yoghurt [4]	CRUSH [2,4]	-	mostly: the jejunum and ileum [2]	food may delay the onset of effect [3,4]	may cause dry mouth and swallowing difficulties [4]
G04BE08	**Tadalafil**	Cialis, Zenavil, Tadalafil (Actavis), Tadilecto	film-coated tablet	CRUSH [4]	can mix with apple puree/yoghurt [4]	CRUSH [4]	-	-	regardless of food [1,4]	-
G04CA01	**Alfuzosin**	Alfuzosin-Teva, Xatral SR	prolonged-release tablet	NO [1,2,4,5]	-	NO [1,2,3,4]	-	mostly: the duodenum and beginning of the jejunum [3]<br>to the jejunum absorption may ↓ [3]	-	if the tablet is crushed, the API is released faster; risk of toxicity [6]<br>**ALTERNATIVE:** change the API [6]
G04CA02	**Tamsulosin**	Flosin, Tamsulosin (Auxilia, Lannacher, Stada)	modified-release capsule (modified pellets)	OPEN CAPSULE DON'T CRUSH! [2]	-	NO [1,2,3]	may form large clumps, risk of FT blockage [3]	the intestine [3,4]	with food GIT side effects ↓ [1,3]	opening the modified or prolong. capsule does not alter the effect of the drug [6]; if the tablet's/capsule's contents are crushed, the API is released faster; risk of toxicity [6]<br>**ALTERNATIVE:** change the API [6]
		Omsal	prolonged-release capsule (modified pellets)							
		Tanyz ERAS	prolonged-release tablet	NO [1,2,5]			-			
G04CA04	**Silodosin**	Urorec	capsule	OPEN CAPSULE [4,5]	cannot be chewed [1,4]<br>can mix with apple puree [4,5]	DISPERSE CAPSULE CONTENTS [4]	-	-	with food [1,4,5]	-
G04CB01	**Finasteride**	Finasteride (Accord, Teva)	film-coated tablet	DISPERSE (may take time) [2,4]	can crush and mix with apple puree/ yoghurt [4]	DISPERSE (may take time) [2,3,4]	rinse [2]<br>FT ≥ CH8 [3]	-	regardless of food [1,3,4,5]	**HAZARDOUS TO PREGNANT!** [1,7]
G04CB02	**Dutasteride**	Avodart, Dutasteride (Teva), Dutrys, Elvim	soft capsule	NO [1,4,5]	cannot be chewed; risk of mucosal damage [1,4,5]	NO [1,4]	-	-	-	**HAZARDOUS TO PREGNANT!** [1,7]<br>absorbed also through the skin! [4,5]<br>**ALTERNATIVE:** change the API [6]
**SYSTEMIC HORMONAL PREPARATIONS, EXCL. SEX HORMONES AND INSULINS**										
H01BA02	**Desmopressin**	Minirin	oral lyophilisate	SUBLINGUALLY [1,2,4,5]	saliva necessary [2]<br>can disperse in a little water [4]<br>can crush and mix with apple puree/ yoghurt [4]	NO [6]	can be administered under the tongue [2,6]<br>risk of aspiration [4,6]	under the tongue [1,2,3], the stomach, duodenum and jejunum [2,3]<br>to the jejunum absorption may vary [2]	alters in case of lower doses; larger doses regardless of food [1,4]	-
						DISPERSE [3,4]	-			
H02AA02	**Fludrocortisone**	Florinef	tablet	DISPERSE [4]	can crush and mix with apple puree/ yoghurt [4,5]	DISPERSE [4]	FT ≥ CH8 [3]	-	with food GIT side effects ↓ [4,5]	-
H02AB02	**Dexamethasone**	Dexamethason KRKA	tablet	CRUSH [2,4]	can mix with apple puree/yoghurt [4]	CRUSH [2,3,4]	FT ≥ CH8 [3]	also suitable to the jejunum [3,2]	with food GIT side effects ↓ [1,4,5]	-
			injection solution	DILUTE [2,4]	-	DILUTE [2,4]	-			
H02AB04	**Methyl-prednisolone**	Medrol	tablet	DISPERSE [2]	-	DISPERSE [2]	-	-	regardless of food [3]<br>with food GIT side effects ↓ [5]	-

ATC code	Active substance	Medicinal product	Dosage form	Oral administration		Administration by FT		Site of absorption/ administration	Administration with food	Remarks
				Guide	Comment	Guide	Comment			
H02AB06	**Prednisolone**	Prednisolon (Jenapharm, Richter)	tablet	DISPERSE (may take time] [2,4]	make half [4]<br>can crush and mix with apple puree/ yoghurt [4]	DISPERSE (may take time) [2,3,4]	make half [4]	-	with food GIT side effects ↓ [1,4,5]	-
H02AB09	**Hydrocortisone**	Abix, Hydrocortison Orion	tablet	DISPERSE (may take time) [1,2,3,4]	can crush and mix with apple puree/ yoghurt [4]	DISPERSE (may take time) [2,3,4]	FT ≥ CH8 [3]	-	with food GIT side effects ↓ [4,5]	-
H03AA01	**Levothyroxine sodium**	L-Thyroxin	tablet	DISPERSE (may take time) [1,4]	can crush and mix with apple puree/ yoghurt [4,5]	DISPERSE (may take time) [2,3,4]	before administration shake vigorously [2,3,4]<br>FT ≥ CH8 [3]	the small intestine [4]	food ↓ absorption [1,4,5]<br>it is recommended to increase the dose by 25mcg when fed by FT; monitor TSH; adjust when changing to a normal diet[5]	light-sensitive, administer immediately! [4]
H03BA02	**Propylthiouracil**	Propylthiouracil, Tiotil	tablet	DISPERSE [2,4]	bitter [2,4]<br>can crush and mix with apple puree/ yoghurt [4]	DISPERSE [2,4]	-	-	regardless of food [4]	-
H03BB02	**Thiamazole**	Thyrozol	film-coated tablet	DISPERSE (takes time) [6]	-	DISPERSE (takes time) [6]	FT ≥ CH8 [6]	-	regardless of food [1,5]	-
**ANTIINFECTIVES FOR SYSTEMIC USE**										
**ANTIBACTERIALS FOR SYSTEMIC USE**										
J01AA02	**Doxycycline**	Doxycyclin (STADA, Vitabalans), Doxylan, Doxy-M-ratiopharm	tablet	DISPERSE (takes time) [1,4]	can crush and mix with apple puree/ yoghurt [4,5]<br>after administration give water/thickened liquid to drink [1,4,5]	DISPERSE (takes time) [4]	-	mostly: the upper small intestine [1,4]<br>to the jejunum absorption may ↓ [4]	with food GIT side effects ↓ [1,4,5]	after administration must remain upright for 0.5-1h, otherwise the risk of oesophageal damage [1,4,5]<br>antacids and drugs containing Mg, Ca, Al, Fe, Zn ↓ absorption administer 2h apart [1,4]<br>may cause swallowing difficulties [4]
J01AA08	**Minocycline**	Minocyclin, Minocyclin-ratiopharm	capsule	OPEN CAPSULE [2]	can mix with apple puree/yoghurt [4]<br>after administration, give water/thickened liquid to drink [1,4,5]	DISPERSE CAPSULE CONTENTS [2]	risk of FT blockage [2]	adequate absorption after administration by FT [4]	with food GIT side effects ↓ [4,5]	antacids and drugs containing Mg, Ca, Al, Fe, Zn ↓ absorption administer 2h apart [1,5]<br>may cause swallowing difficulties [4]
		Minocyclin Hexal	tablet	CRUSH [2]		CRUSH [2]	-			
J01CE02	**Phenoxymethyl-penicillin**	Ospen	oral suspension	YES [1,2,4]	not suitable if cannot swallow fluids [4]	DILUTE [2,3,4]	-	to the upper GIT [1]<br>needs stomach acid [3]	regardless of food [1,4]<br>with ETF bioavailability ↓; stop ETF 1-2h before and 2h after [2,3]	-
			film-coated tablet	CRUSH [4]	can mix with apple puree/yoghurt [4]	CRUSH [4]				

ATC code	Active substance	Medicinal product	Dosage form	Oral administration		Administration by FT		Site of absorption/ administration	Administration with food	Remarks
				Guide	Comment	Guide	Comment			
J01CA04	**Amoxicillin**	Amoxicillin (Sandoz, Ratiopharm TS), Ospamox	powder for oral suspension	YES (look at SmPC) [1,2,4]	not suitable if cannot swallow fluids [4]	DILUTE [2,3,4]	-	the duodenum and upper jejunum [2,3,4] conflicting data when administered to the jejunum [2,3]	regardless of food [1,3,5]	-
		Amoxicillin (Sandoz, Ratiopharm), Ospamox	film-coated tablet	CRUSH [2]	can mix with apple puree/yoghurt [4]	CRUSH [2,3]				
			powder for injection /infusion solution	DISSOLVE [2]	-	DISSOLVE [2]	recommended for administration to the jejunum [2]			
J01CR02	**Amoxicillin + clavulanic acid**	Amoksiklav, Augmentin Fruit, Medoclav	powder for oral suspension	YES (look at SmPC) [1,4]	not suitable if cannot swallow fluids [4]	DILUTE [4]	without diluting, high risk of FT blockage [3,4]	amoxicillin: the duodenum and upper jejunum [4] clavulanic acid: varies [4]	with food GIT side effects ↓ [1,4,5]	-
		Amoksiklav	dispersible/ orodispersible tablet	DISPERSE IN MOUTH [1]	can disperse [1,3] bad taste [6]	DISPERSE [3]	-			
		Amoksiklav, Amoxicillin /Clavulanic acid (Actavis), Augmentin, Betaklav, Medoclav	film-coated tablet	CRUSH [4]	can mix with apple puree/yoghurt [4]	CRUSH [4]				
J01CR04	**Sultamicillin**	Unasyn	film-coated tablet	DISPERSE (takes time) [6]	-	DISPERSE (takes time) [6]	FT ≥ CH10 [6]	-	regardless of food [1]	-
J01DB05	**Cefadroxil**	Duracef	soluble tablet	YES [1]	not suitable if cannot swallow fluids [4]	NO [6]	suspension; risk of FT blockage [6]	-	regardless of food [1,5]	to prevent any loss of the medicine, open the capsule above the cup [6]
			capsule	OPEN CAPSULE [6]	-	DISPERSE CAPSULE CONTENTS (in cup) [6]	FT ≥ CH10 [6]			
			powder for oral suspension	YES (look at SmPC) [1,2]	not suitable if cannot swallow fluids [6]	DILUTE [2]	-			
J01DC02	**Cefuroxime**	Axetine, Furocef, Zinnat	film-coated/coated tablet	DISPERSE [3,4]	bitter [4,5] disperse in juice [4] can crush and mix with apple puree/ yoghurt [4]	DISPERSE [2,3,4]	Zinnat tablet disperses well in water, prefer to suspension [6]	becomes the active form in the intestinal wall [1,3] contradictory info about jejunal administration [2,3]	for optimal absorption after a meal [1,3,4,5]	in jejunal administration, prefer tablets because the osmolarity is lower [3] the tablet and suspension are not bioequivalent [1,4] may cause dry mouth [4]
		Zinnat	granules for oral suspension	YES (look at SmPC) [1,2]	can dilute in juice/milk[4]	DILUTE [2,3]	risk of FT blockage [2,3] FT ≥ CH10 [3]			
J01DC10	**Cefprozil**	Cefzil	powder for oral suspension	YES (look at SmPC) [1]	not suitable if cannot swallow fluids [6]	NO [6]	no info, risk of FT blockage [6]	-	regardless of food [1,5]	**ALTERNATIVE:** change the API [6]
			film-coated tablet	CRUSH [6]	-					
J01EA01	**Trimethoprim**	Trimetop	tablet	DISPERSE [4]	can crush and mix with apple puree/ yoghurt [4]	DISPERSE [4]	-	-	food ↓ absorption [2,3] with food GIT side effects ↓ [1,4,5]	-
J01EE01	**Sulfa-methoxazole + trimethoprim**	Bactrim (DS, Forte)	syrup	YES [1]	not suitable if cannot swallow fluids [6]	DILUTE [6]	-	-	with food GIT side effects ↓ [4]	-
			tablet	DISPERSE [4]	can crush and mix with apple puree/ yoghurt [4]	DISPERSE [4]				

ATC code	Active substance	Medicinal product	Dosage form	Oral administration		Administration by FT		Site of absorption/ administration	Administration with food	Remarks
				Guide	Comment	Guide	Comment			
J01FA09	**Clarithromycin**	Klacid, Lekoklar	granules for oral suspension	YES (look at SmPC) [1,2,4]	bitter aftertaste [1] not suitable if cannot swallow fluids [4]	DILUTE [2,3,4]	without diluting, risk of FT blockage [2,3,4] FT ≥ CH9 [3]	mostly: the jejunum [3] absorption by gastric FT is adequate [4], probably also to the jejunum [3]	regardless of food [1,3,4]	if the modified tablet is crushed, the API is released faster, risk of toxicity [6] may cause taste disturbances [4]
		Fromilid, Klabax, Klacid, Klerimed	film-coated tablet	DISPERSE [4]	can crush and mix with apple puree/ yoghurt [4]	DISPERSE [4]	-			
		Fromilid UNO, Klacid SR	modified-release tablet	NO [1,5]	-	NO [1,3]				
J01FA10	**Azithromycin**	Sumamed	powder for oral suspension	YES (look at SmPC) [2,4]	not suitable if cannot swallow fluids [4]	DILUTE [2,4]	-	-	regardless of food [4,5]	to prevent any loss of the medicine, open the capsule above the cup [6] may cause taste disturbances [4]
		Azitromycin (Actavis, Grindex, KRKA,Sandoz), Sumamed, Zylumit	film-coated tablet	CRUSH [4]	can crush and mix with apple puree/ yoghurt [4]	CRUSH [4]				
		Azitrox		DISPERSE (takes time) [6]		DISPERSE (takes time) [6]	FT ≥ CH10 [6]			
		Sumamed	capsule (powder)	OPEN CAPSULE [6]	can mix with apple puree/yoghurt [6]	DISPERSE CAPSULE CONTENTS (in cup) [6]	FT ≥ CH8 [6]			
J01FF01	**Clindamycin**	Clindamycin MIP	film-coated tablet	CRUSH [6]	bitter [6] can mix with apple puree/yoghurt [6]	CRUSH [6]	FT ≥ CH8 [6]	-	regardless of food [1,4,5]	may cause inflammation of the oesophagus, give extra water to drink after swallowing [1,4]
		Dalacin C	capsule	OPEN CAPSULE [2,4]	bitter, mix with juice [2,4] can mix with apple puree/yoghurt [4]	DISPERSE CAPSULE CONTENTS [2,4]	min 3ml [4] FT ≥ CH8 [2,3]			
J01MA01	**Ofloxacin**	Ofloxin	film-coated tablet	CRUSH [2]	-	CRUSH [2,3]	FT ≥ CH8 [3]	to the jejunum absorption may ↓ [2]	on an empty stomach [1,2] stop ETF 1-2h before and 2h after [2,3]	use water for injections since it may form chelates with tap water [2]
J01MA02	**Ciprofloxacin**	Ciprinol, Ciprofloxacin (Olanfarm, Sandoz)	film-coated tablet	CRUSH [4]	bitter [2] can mix with apple puree [4]	CRUSH [2,3,4]	parenteral administration is preferred [2,5] FT ≥ CH8 [3]	the duodenum (a little the jejunum) [2,4] avoid administration to the jejunum or use higher doses [2,3,4]	food, dairy products, antacids, drugs containing Mg, Ca, Al, Fe, Zn ↓ absorption; administer 2h before or 6h after a meal [1,5] ETF ↓ absorption >30%, stop ETF 1-2h before and 2h after [2,3,4,5]	use water for injections since it may form chelates with tap water [2]
J01MA06	**Norfloxacin**	Nolicin	film-coated tablet	CRUSH [2,4]	bitter [2,4] can mix with apple puree [4]	CRUSH [2,4]	-	-	food and dairy products ↓ absorption, administer 1h before or 2h after a meal [5] antacids, drugs containing Mg, Ca, Al, Fe, Zn and ETF ↓ absorption; stop ETF 2h before and after [5]	the crushed tablet may stick to the bottom of the mortar; rinse [4] light-sensitive, administer immediately! [2]
J01MA12	**Levofloxacin**	Tavanic	film-coated tablet	CRUSH [2,3]	crush in water to prevent dust [2,3]	CRUSH [2,3]	crush in water to prevent dust [2,3] FT ≥ CH8 [3]	to the jejunum absorption may ↓ [2]	antacids, drugs containing Mg, Ca, Al, Fe, Zn and ETF ↓ absorption; administer 1-2h before and after a meal [2,3,5]	-

ATC code	Active substance	Medicinal product	Dosage form	Oral administration		Administration by FT		Site of absorption/ administration	Administration with food	Remarks
				Guide	Comment	Guide	Comment			
J01MA14	**Moxifloxacin**	Avelox, Floxelan	film-coated tablet	DISPERSE (takes time) [4]	bitter [2,4] can crush and mix with apple puree [4]	DISPERSE (takes time) [4]		absorbed well when administered by nasogastric FT [4]	regardless of food [4,5] antacids, drugs containing Mg, Ca, Al, Fe, Zn and ETF ↓ absorption; administer 4h before or 8h after [5]	may cause swallowing difficulties [4]
J01XD01	**Metronidazole**	Metronidazol Nycomed	film-coated tablet	DISPERSE (takes time) [2,4]	can crush and mix with apple puree/ yoghurt [4]	DISPERSE (takes time) [2,3,4]	FT ≥ CH8 [3]	the small intestine; needs stomach acid [2,3] to the duodenum and jejunum absorption may ↓ [4]	for treatment of systemic infections on an empty stomach, for treatment of local infections with food [2]	may cause taste disturbances [4]
J01XE01	**Nitrofurantoin**	Nitrofurantoin Nycomed	tablet	DISPERSE (takes time) [6]	can crush and mix with apple puree/ yoghurt [4]	DISPERSE (takes time) [3,6]	FT ≥ CH8 [3]	-	food ↑ absorption [1,2,4] with food GIT side effects ↓ [4]	-
J01XX01	**Fosfomycin**	Fosfomycin Sandoz	powder for oral solution	YES (look at SmPC) [1,4,5]	dissolve in 90-150ml of water [1,4,5] can dissolve in 5ml of water and mix with apple puree/yoghurt [4]	DISSOLVE (90ml) [4]	may be dissolved in smaller volumes for patients with fluid restriction (10ml) [4,6]	the small intestine [4]	administer 2h before or 2h after a meal [1,4]	preferably should be administered before going to bed and after emptying the bladder [1]
J01XX08	**Linezolid**	Apel, Linezolid (KRKA)	film-coated tablet	CRUSH [2,4]	can mix with apple puree/yoghurt [4]	CRUSH [2,4]	bioavailability can ↓ when administered to the jejunum [2]	the stomach and small intestine [2,3,4] well-absorbed by gastric FT [4]; absorption may ↓ by FT to the small intestine [2,3]	regardless of food [3,4,5]	-
**ANTIMYCOTICS FOR SYSTEMIC USE**										
J02AB01	**Miconazole**	Daktarin	oral gel	YES [1]	-	NO [6]	only topically for the treatment of oral candidiasis; the risk of aspiration must be ruled out [6]	-	-	**ALTERNATIVE:** change the API [6]
J02AB02	**Ketoconazole**	-	tablet	CRUSH [2]	-	CRUSH [2]	-	acidic environment is needed for absorption [1,2,3,5] to the small intestine absorption may ↓ [2,3]	food in general ↑ absorption, but ETF may↓, since ↑ GIT's pH[2]	-
J02AC01	**Fluconazole**	Diflazon, Diflucan, Fluconazole (Actavis, Elvim, Medochemie)	capsule (powder)	OPEN CAPSULE [2,4]	can mix with apple puree/yoghurt [4]	DISPERSE CAPSULE CONTENTS [2,3,4]	-	also suitable to the duodenum and jejunum [3,4]	regardless of food [1,3,4,5]	**-**
J02AC02	**Itraconazole**	Cladosol, Itraconazol (Actavis), Procanazol	capsule (micro granules)	OPEN CAPSULE; DON'T CRUSH! [2,4]	mix with acidic juice/apple puree/ yoghurt [4]	NO [2,3,4]	risk of FT blockage [2,3,4]	acidic environment is needed for absorption [1,3,4,5]	after a meal [1,5]	**ALTERNATIVE:** change the API [6]
J02AC03	**Voriconazole**	Verria, Vfend, Voriconazole (Teva)	film-coated tablet	CRUSH [2,4]	can mix with apple puree/yoghurt [4]	CRUSH [2,3,4]	-	also suitable to the jejunum [2,4] absorption may ↓ if administered by FT [5]	high-fat foods ↓ absorption; administer 1h before or after a meal [1,4,5] no delay is required when ETF is used [4]	**-**

ATC code	Active substance	Medicinal product	Dosage form	Oral administration		Administration by FT		Site of absorption/ administration	Administration with food	Remarks
				Guide	Comment	Guide	Comment			
J02AC04	**Posaconazole**	Noxafil, Posaconazole (AEKL)	oral suspension	YES [1,4]	not suitable if cannot swallow fluids [4]	DILUTE [4]	-	acidic environment is needed for absorption [3,4] to the small intestine absorption may ↓ [3,5]	after a meal (only suspensions) [1,4,5]	suspension and a gastro-resistant tablet are not bioequivalent [4] the release of the API changes when the gastro-resistant tablet is crushed; the effect may ↓ [6] absorption may ↓ when administering with drugs that reduce stomach acid ↓ [4] may cause dry mouth and taste disturbances [4]
		Noxafil, Posaconazole (Accord)	gastro-resistant tablet	NO [1,4,5]	-	NO [1,3,4]				
J02AC05	**Isavuconazole**	Cresemba	capsule	OPEN CAPSULE [5]	-	DISPERSE CAPSULE CONTENTS [4,5]	-	-	regardless of food [1,4,5]	-
**ANTIMYCOBACTERIALS**										
J04AB02	**Rifampicin**	Rifampicyna TZF Rifasynt	capsule	OPEN CAPSULE [4,5]	can mix with apple puree/yoghurt [4,5]	NO [4]	risk of FT blockage [4]	the stomach and duodenum [2,4]	food ↓ absorption [2,4,5] administer 30min before or 2h after a meal [2,3,4,5]	is dark in colour, can stain [4]
		Eremfat	tablet	CRUSH [4]		CRUSH [4]	-			
		Rifadin	oral suspension	YES [4]		DILUTE [3,4]				
			powder for injection /infusion solution	YES (look at SmPC) [4]	prepare according to the SmPC and administer undiluted [4]	YES (look at SmPC) [4]	prepare according to the SmPC and administer undiluted [4]			
J04AC01	**Isoniazid**	Isoniazid	tablet	DISPERSE (takes time) [2,4,5]	can crush and mix with apple puree/ yoghurt [4,5]	DISPERSE (takes time) [2,3,4]	FT ≥ CH8 [3]	absorbed well by nasogastric FT [4]	food ↓ absorption [2,4,5] administer 30min before or 2h after a meal [2,3,4,5]	-
J04AK01	**Pürazinamide**	Pirazinamida, Pyrafat	tablet	DISPERSE (may take time) [2,4]	can crush and mix with jam/chocolate/ nougat cream [4]	DISPERSE (may take time) [2,4]	-	absorbed well by nasogastric FT [4]	regardless of food [3,4]	-
J04AK02	**Ethambutol**	Etambutol	tablet	CRUSH [2,4,5]	bitter [5] can be mixed with apple juice/puree/ yoghurt [4,5]	CRUSH [2,3,4]	-	absorbed well by nasogastric FT [4]	regardless of food [3,4] with food GIT side effects ↓[5]	do not administer Al-containing antacids within 4h of administration [4]
J04BA01	**Clofazimine**	Lamprene	soft capsule (oil-wax content)	NO [4,5]	-	NO [4,5]	-	-	-	**ALTERNATIVE:** change the API [6]
**ANTIVIRALS FOR SYSTEMIC USE**										
J05AB01	**Aciclovir**	ACIC	tablet	DISPERSE (50ml) [3,4]	can crush and mix with apple puree/ yoghurt [4]	DISPERSE [3,4]	-	the duodenum and jejunum[4] to the jejunum absorption may ↓ [3,4]	regardless of food [3,4]	-
J05AB11	**Valaciclovir**	Valaciclovir (Actavis, Elvim), Valtrex	film-coated tablet	CRUSH [3,4]	bitter [4] can mix with apple puree/yoghurt [4]	CRUSH [3,4]	-	-	regardless of food [1,3,4] with food GIT side effects ↓[5]	It is hard to crush the tablet [3,4] the API degrades rapidly, administer immediately [3] may cause dry mouth [4]

ATC code	Active substance	Medicinal product	Dosage form	Oral administration		Administration by FT		Site of absorption/ administration	Administration with food	Remarks
				Guide	Comment	Guide	Comment			
J05AB14	**Valganciclovir**	Valcyte	powder for oral solution	YES (look at SmPC) [1,2,4]	not suitable if cannot swallow fluids [4]	DILUTE [4]	-	-	food ↑ absorption [4,5] with food [1,4,5]	**HAZARDOUS TO PREGNANT!** [1,7]
		Valganciclovir (Sandoz)	film-coated tablet	NO [1,4]	hazardous to handler [1,4]	NO [1,4]	hazardous to handler [1,4]			
J05AE03	**Ritonavir**	Ritonavir (Mylan)	film-coated tablet	NO [2,5]	unpleasant taste [2,4,5]	NO [2,5]	-	absorbed by gastric FT [4]	with food [1,4,5]	bioavailability may change upon crushing [4] **ALTERNATIVE:** change the API [6]
J05AF01	**Zidovudine**	Retrovir	oral solution	YES [1,2,4]	not suitable if cannot swallow fluids [4]	DILUTE [2,3,4]	-	the small intestine [1,4] absorbed by gastric FT [4]	regardless of food [1,3,4,5]	-
J05AF05	**Lamivudine**	Epivir	oral solution	YES [1,2,4]	not suitable if cannot swallow fluids [4]	DILUTE [2,4]	-	the small intestine [4]	regardless of food [1,3,4,5]	the bioavailability of the crushed tablet is equivalent to the whole [1]
			film-coated tablet	CRUSH [1,4]	can mix with apple puree/yoghurt [1,4]	CRUSH [4]				
J05AF06	**Abacavir**	Ziagen	oral solution	YES [1,2,4]	not suitable if cannot swallow fluids [4]	DILUTE [2,4]	-	probably: the duodenum [4] absorbed by gastric FT; to the jejunum absorption may ↓[4]	regardless of food [1,3,4,5]	crushing may change bioavailability, **MONITOR** the viral load [4]
			film-coated tablet	CRUSH [1,4]	can mix with apple puree/yoghurt [1,4]	CRUSH [3,4]				
J05AF07	**Tenofovir disoproxil**	Tenofovir disoproxil (Mylan)	film-coated tablet	DISPERSE (100ml) (takes time) [1,4]	bitter [5] can disperse in juice[1,4] can crush and mix with apple puree/ yoghurt [4,5]	DISPERSE (takes time) [3,4]	settles quickly, shake [4] FT ≥ CH8 [3]	the small intestine [4]	food ↑ absorption [4] with food [1,3,4]	crushing may change bioavailability, **MONITOR** the viral load [4]
J05AF10	**Entecavir**	Baraclude, Entecavir (Accord)	film-coated tablet	DISPERSE [4]	can crush and mix with apple puree/ yoghurt [4]	DISPERSE [4]	shake, the tablet may stick to the syringe [4]	-	regardless of food [1]	tablet and oral solution are bioequivalent [1]
J05AF13	**Tenofovir alafenamide**	Vemlidy	film-coated tablet	CRUSH [4]	-	CRUSH [4]	-	-	food ↑ absorption [4] with food [1,5]	crushing may change bioavailability, **MONITOR** the viral load [4]
J05AG05	**Rilpivirine**	Edurant	film-coated tablet	DISPERSE (10min shaking) [4]	-	DISPERSE (10min shaking) [4]	-	needs stomach acid [4] absorbed by gastric FT [4]; not suitable to the jejunum [4]	food ↑ absorption [1,4,5] with food [1,4,5]	light-sensitive, administer immediately! [4]
J05AH02	**Oseltamivir**	Tamiflu	capsule	OPEN CAPSULE [1,4,5]	can mix with apple puree/yoghurt [2,3,4]	DISPERSE CAPSULE CONTENTS [2,3,4,5]	FT ≥ CH8 [3]	absorbed by gastric FT [4]	regardless of food [1,2,4,5] with food GIT side effects ↓ [2,4,5]	-
J05AJ01	**Raltegravir**	Isentress	granules for oral suspension	YES (look at SmPC) [1]	not suitable if cannot swallow fluids [6]	DILUTE [6]	-	ileum [4] absorbed by gastric FT [4]	regardless of food [1,4,5]	crushing may change bioavailability, **MONITOR** the viral load [4]
			chewable tablet	CHEW [1,4,5] or DISPERSE [4]	can crush and mix with apple puree/ yoghurt [4,5]	DISPERSE [4]				
			film-coated tablet	NO [1,5]	-	NO [1,5]				
J05AJ03	**Dolutegravir**	Tivicay	film-coated tablet	DISPERSE [1]	can crush and mix with apple puree/ yoghurt [4,5]	DISPERSE [1]	-	after crushing is well absorbed orally or by gastric FT [4]	regardless of food [1,4,5]	Ca and Fe ↓ absorption [4] crushing may change bioavailability, **MONITOR** the viral load [4]

ATC code	Active substance	Medicinal product	Dosage form	Oral administration		Administration by FT		Site of absorption/ administration	Administration with food	Remarks
				Guide	Comment	Guide	Comment			
**ANTICANCER DRUGS**										
	**ALL ACTIVE SUBSTANCES**	-	oral solutions/ suspensions	YES [1,4]	-	YES [1,4]	-	depends on the API [6]	depends on the API [6]	**HAZARDOUS DRUG!** carcinogenic and teratogenic [4,6]<br>consult with a pharmacist [6]
			tablets/capsules	NO [1,4]		NO [1,4]				
**IMMUNOSUPPRESSANTS**										
L04AA06	**Mycophenolic acid**	Cellcept	powder for oral suspension	YES (look at SmPC) [1]	not suitable if cannot swallow fluids [6]	YES (look at SmPC) [2,3,4,5]	do not dilute [3,5]<br>FT ≥ CH8 [2,3,4,5]	partly: the stomach, mostly: the duodenum and jejunum[3] suitable to the duodenum, but not to the jejunum [4]	regardless of food [1,3,5]<br>with food GIT side effects ↓ [4]	**HAZARDOUS TO PREGNANT!** [1,7]<br>crushing the gastro-resistant tablet alters the API release [6]<br>may cause mouth ulcers, swallowing difficulties and the loss of taste [4]
			capsule (powder)	NO [1,2,3,4,5]	hazardous to handler [1,4,7]	NO [1,2,3,4,5]	hazardous to handler [1,4,7]			
		Cellcept, Mycophenolate Mofetil (Accord),Myfenax	film-coated tablet							
		Myfortic	gastro-resistant tablet							
		-	powder for infusion solution concentrate	YES (look at SmPC) [6]	prepare according to the SmPC and administer undiluted [2,4]	YES (look at SmPC) [2,4]	prepare according to the SmPC and administer undiluted [2,4]			
L04AA10	**Sirolimus**	Rapamune	oral solution	YES [1,4,5]	mix with 60ml (thickened) water/ orange juice	DILUTE (60ml) [4]	-	bioavailability may ↓ following administration by FT [4]	high-fat food ↑ absorption [4]<br>administer at the same time regard to food [4,5]	**HAZARDOUS DRUG!** [7]<br>tablet and oral solution are not bioequivalent [4]<br>when switching from a tablet to a solution, start with the same dose; adjust according to TDM values [1,4]
			coated tablet	NO [1,4,5]	hazardous to handler [4,5]	NO [1,4,5]	hazardous to handler [4,5]			
L04AA13	**Leflunomide**	Arava	film-coated tablet	DISPERSE (takes time) [4]	prepare by shaking [4]	DISPERSE (takes time) [4]	prepare by shaking [3,4]<br>FT ≥ CH8 [3]	becomes active in the wall of the small intestine [3]	regardless of food [1,3,4,5]	**HAZARDOUS DRUG!** [1,7]<br>may cause dry mouth, mouth ulcers and taste disturbances [4]
L04AA27	**Fingolimod**	Gilenya	capsule	OPEN CAPSULE [4]	-	DISPERSE CAPSULE CONTENTS [4]	-	-	regardless of food [4]	**HAZARDOUS DRUG!** [1,7]<br>due to the long half-life, short-term discontinuation of treatment may be possible [4]
L04AA29	**Tofacitinib**	Xeljanz	film-coated tablet	CRUSH [1]	-	CRUSH [6]	-	-	regardless of food [1,4,5]	**HAZARDOUS DRUG!** [7]<br>if the prolong. tablet is crushed, the API is released faster; risk of toxicity [6]
			prolonged-release tablet	NO [1]		NO [1]				
L04AA31	**Teriflunomide**	Aubagio	film-coated tablet	CRUSH [4]	-	CRUSH [4]	-	-	regardless of food [1,4,5]	**HAZARDOUS DRUG!** [1,7]<br>may cause mouth ulcers [4]
L04AA40	**Cladribine**	Mavenclad	tablet	NO [1,4,5]	hazardous to handler [1,4,5]	NO [1,4,5]	hazardous to handler [1,4,5]	-	-	**HAZARDOUS DRUG!** [1,7]<br>consult with a pharmacist [6]
L03AA42	**Siponimod**	Mayzent	film-coated tablet	NO [1]	hazardous to handler [1,5]	NO [1]	hazardous to handler [1,5]	-	-	consult with a pharmacist [6]

ATC code	Active substance	Medicinal product	Dosage form	Oral administration		Administration by FT		Site of absorption/ administration	Administration with food	Remarks
				Guide	Comment	Guide	Comment			
L04AA44	**Upadacitinib**	Rinvoq	prolonged-release tablet	NO [1,4,5]	-	NO [1,4,5]	-	-	-	if the prolonged-release tablet is crushed, the API is released faster, risk of toxicity [6]<br>consult with a pharmacist [6]
L04AD01	**Ciclosporin**	Sandimmun (Neoral, Optoral)	oral solution	YES [1,2,4]	bad taste; dilute with orange/apple juice/ soft drink [1,2,3,4,5]<br>can mix with apple puree [4]	DILUTE [3,4]	dilute with orange/ apple juice [3,4]<br>the solution is very oily and may stick to the FT [4]	the duodenum and jejunum [3,4]	administer at the same time regard to food [4,5]	**HAZARDOUS DRUG!** [1,7]<br>not compatible with PVC cups and syringes [5]
		Equoral, Sandimmun Neoral	Soft capsule	NO [1,4]	hazardous to handler [4]	NO [1,4]	hazardous to handler [4]			
L04AD02	**Tacrolimus**	Modigraf	granules for oral suspension	YES<br>(look at SmPC) [1,2,5]	prefer! [2]<br>not suitable if cannot swallow fluids [6]	YES [1,2,3,5]	prefer! [2]	the whole GIT [1,3]<br>absorption may ↓ when administered by FT [4]	food ↓ absorption, 1h before or 2-3h after a meal [1,3,4,5]	**HAZARDOUS DRUG!** [7]<br>not compatible with PVC cups and syringes [5]<br>the capsule's contents/granules or a suspension mixed with them are administered sublingually and buccally [4]<br>opening the prolong. capsule does not alter the effect of the drug [6]; if the prolong. tablet's/capsule's contents are crushed, the API is released faster; risk of toxicity [6]
		Advagraf, Dailiport	prolonged-release capsule	OPEN CAPSULE;<br>DON'T CRUSH! [4]	mix with 50ml of water [4]	DISPERSE CAPSULE CONTENTS (50ml)<br>DON'T CRUSH! [4]	-			
		Envarsus	prolonged-release tablet	NO [1,6]	-	NO [1,6]	-			
L04AX01	**Azathioprine**	Atsimutin, Imuran	film-coated tablet	DISPERSE<br>(takes time) [3,4]	-	DISPERSE<br>(takes time) [3,4]	FT ≥ CH8 [3]	suitable to the duodenum and jejunum, but absorption may ↑ when administered by FT [4]	regardless of food [3]<br>with food GIT side effects ↓ [1,3,4,5]	**HAZARDOUS DRUG!** [1,7]<br>may cause mouth ulcers and taste disturbances [4]
L04AX02	**Thalidomide**	Thalidomide (Celgene)	capsule	NO [1,4]	hazardous to handler [1,4,5,7]	NO [1,4]	hazardous to handler [1,4,5,7]	-	slows absorption [1]<br>1h after dinner [4,5]	**HAZARDOUS DRUG!** [1,4,7]<br>may cause dry mouth [4]<br>consult with a pharmacist [6]
L04AX03	**Methotrexate**	Trexan	tablet	DISPERSE [4]	-	DISPERSE [4]	-	-	acidic drinks ↑ toxicity [4]	**HAZARDOUS DRUG!** [1,4,5,7,8]<br>consult with a pharmacist [6]
			injection solution	DILUTE [4]		DILUTE [4]				
L04AX04	**Lenalidomide**	Lenalidomide (Accord, Auxilia, Grindex, Zentiva, Teva) Revlimid	capsule (powder)	NO [1,4,5]	hazardous to handler [1,4,5,7]	NO [1,4,5]	hazardous to handler [1,4,5,7]	-	regardless of food [1,4,5]	**HAZARDOUS DRUG!** [1,4,5,7,8]<br>consult with a pharmacist [6]
L04AX06	**Pomalidomide**	Imnovid	capsule	NO [1,4,5]	hazardous to handler [1,4,7]	NO [1,4,5]	hazardous to handler [1,4,7]	-	regardless of food [1,4,5]	**HAZARDOUS DRUG!** [1,4,7]<br>consult with a pharmacist [6]
L04AX07	**Dimethyl fumarate**	Tecfidera	gastro-resistant capsule (microtablet)	NO [1,4,5]	-	NO [1,4,5]	-	starting from the small intestine [1,4]	-	the release of the API changes when the capsule's contents are crushed, risk of GIT irritation [1]<br>consult with a pharmacist [6]
**MUSCULO-SKELETAL SYSTEM**										
**ANTIINFLAMMATORY AND ANTIRHEUMATIC PRODUCTS**										
M01AB01	**Indometacin**	Indomet Ratiopharm	capsule (granules)	OPEN CAPSULE [4]	can mix with apple puree/yoghurt [4]	DISPERSE CAPSULE CONTENTS [4]	absorption can ↓ and the risk of GIT side effects can ↑ [4]	-	regardless of food;<br>with food GIT side effects ↓ [1,4,5]	administration by FT is not recommended [3,4]<br>**ALTERNATIVE:** change the API [6]

ATC code	Active substance	Medicinal product	Dosage form	Oral administration		Administration by FT		Site of absorption/ administration	Administration with food	Remarks
				Guide	Comment	Guide	Comment			
M01AB05	**Diclofenac**	Dicuno, Voltaren Akti, Volxol	film-coated tablet	CRUSH [1,4]	can mix with apple puree/yoghurt [4]	CRUSH [4]	-	-	regardless of food;<br>with food GIT side effects ↓ [1,4,5]	opening the prolong. capsule does not alter the effect of the drug [6]; if the modified or prolong. tablet's/capsule's contents are crushed, the API is released faster; risk of toxicity [6]<br>may irritate the mucosa [4]<br>**ALTERNATIVE:** rectal or local diclophenac OR change the API [3,4,6]
		Dicloberl retard, Olfen Depocaps	prolonged-release capsule (microgranules)	OPEN CAPSULEI [6]	-	NO [2,3,4,5]				
		Diclac 100, Diclomelan retard, Olfen Depotabs	prolonged-release tablet	NO [2,4,5]						
		Diclac ID	modified-release tablet							
M01AB16	**Aceclofenac**	Betiral	film-coated tablet	CRUSH [2]	-	CRUSH [2]	-	-	regardless of food;<br>with food GIT side effects ↓ [1,3]	crushing the tablet increases the risk of GIT side effects [2]
M01AC01	**Piroxicam**	Brexin	tablet	NO [6]	no info [6]	NO [6]	no info [6]	-	-	**ALTERNATIVE:** change the API [6]
M01AC05	**Lornoxicam**	Xefo rapid	film-coated tablet	NO [6]	no info [6]	NO [6]	no info [6]	-	-	**ALTERNATIVE:** change the API [6]
M01AC06	**Meloxicam**	Melox, Meloxicam Ratiopharm, Mexolan, Movalis, Recoxa	tablet	DISPERSE (takes time) [4]	can crush and mix with apple puree/ yoghurt [4]	DISPERSE (takes time) [4]	-	-	with food GIT side effects ↓ [4,5]	-
M01AE01	**Ibuprofen**	Nurofen Forte, Ibustar (Forte)	oral suspension	YES [1,4]	not suitable if cannot swallow fluids [6]	DILUTE [3,4]	-	starting from the stomach and mostly the small intestine [1]	delays absorption [1,3,4]<br>with food GIT side effects ↓ [1,3,4,5]	If the prolong. tablet is crushed, the API is released faster; risk of toxicity [6]<br>transient burning sensation in the mouth or throat after administration [1,4]
		Brufen, Brumare	effervescent granules	DISPERSE (look at SmPC) [1]	can disperse with 20ml of water [3]	DISPERSE (in cup) [3,6]	rinse the cup and syringe [6]<br>FT ≥ CH8 [6]			
		Ibumax, Ibumetin, Ibuprofen (Grindeks, Lannacher), Ibustar, Nurofen (Forte/Express)	film-coated/coated tablet	CRUSH [4]	can mix with apple puree/yoghurt [4]	CRUSH [4]	-			
		Nurofen Orange	chewable soft capsule	CHEW [1]	-	NO [1,3]				
		Forsium	Soft capsule	NO [1]	-					
		Brufen	prolonged-release tablet							
M01AE02	**Naproxen**	Epromul, Nalgesin Forte, Nalgesin S	film-coated tablet	CRUSH [2,4]	can mix with apple puree [4]	CRUSH [2,3,4]	-	dissolves in the stomach [1]<br>the small intestine [1,3]<br>probably also suitable to the jejunum [4]	with food GIT side effects ↓ [1,3,4,5]	does not disperse well, is not easy to crush [4]
M01AE52	**Naproxen + esomeprazole**	Vimovo	modified-release tablet	NO [1,2,4,5]	-	NO [1,2,3,4,5]	-	-	-	naproxen is under the first tablet coating; esomeprazole is under an internal gastro-resistant coating [1]<br>**ALTERNATIVE:** change the API [6]
M01AE03	**Ketoprofen**	Keto	film-coated tablet	DISPERSE [6]	can crush and mix with apple puree/ yoghurt [4]	DISPERSE [6]	FT ≥ CH10 [6]<br>constantly shaking: FT ≥ CH8 [6]	-	food may ↓ absorption [1,3]<br>with food GIT side effects ↓ [1,5]	-

ATC code	Active substance	Medicinal product	Dosage form	Oral administration		Administration by FT		Site of absorption/ administration	Administration with food	Remarks
				Guide	Comment	Guide	Comment			
M01AE17	**Dexketoprofen**	Dolmen	oral solution (in bag)	YES [1]	can mix with apple puree/yoghurt [6]	DILUTE [6]	-	-	slows absorption [1]<br>with food GIT side effects ↓ [1]	-
		Dolmen, Ketesse	granules for oral solution	YES (look at SmPC) [1,6]		DISSOLVE [6]	check that the granules are completely dissolved [6]			
		Dolmen, Dekenor, Ketesse	film-coated tablet	CRUSH [6]		NO [6]	no info, risk of FT blockage [6]			
M01AH01	**Celecoxib**	Aclexa, Celebrex	capsule (granules)	OPEN CAPSULE [1,2,4,5]	can mix with apple puree/yoghurt [4,5]	DISPERSE CAPSULE CONTENTS [3,4]	FT ≥ CH8 [3]	-	slows absorption [1]<br>with food GIT side effects ↓ [1,4,5]	may cause dry mouth and swallowing difficulties [4]
M01AH05	**Etoricocib**	Arcoxia, Etoricoxib (Zentiva, Teva), Etoxib	film-coated tablet	DISPERSE [4]	can crush and mix with apple puree/ yoghurt [4]	DISPERSE [3,4]	the coating may not dissolve; risk of FT blockage [3]	-	slows absorption [1,3]<br>with food GIT side effects ↓ [1,4]	may cause dry mouth [4]
M01AX01	**Nabumetone**	Relifex	dispersible tablet	DISPERSE [1,2]	-	DISPERSE [1,2]	-	the small intestine [3]	usual food does not alter [1,3], but milk and probably ETF ↑ absorption, administer separately [2,3]	-
			film-coated tablet	NO [2]	irritating!	NO [2]	irritating!			
**MUSCLE RELAXANTS, ANTIGOUT PREPARATIONS AND DRUGS FOR TREATMENT OF BONE DISEASES**										
M03BX01	**Baclofen**	Baclosal	tablet	DISPERSE (takes time) [4]	can crush and mix with apple puree/ yoghurt [4]	DISPERSE (takes time) [3,4]	FT ≥ CH8 [3]	-	regardless of food;<br>with food GIT side effects ↓ [3,4,5]	-
M03BX02	**Tizanidine**	Sirdalud	tablet	CRUSH [2,3]	can mix with apple puree [5]	CRUSH [2,3]	FT ≥ CH8 [3]	-	regardless of food [3]	-
M04AA01	**Allopurinol**	Allopurinol (Accord, Nycomed, Sandoz)	tablet 100mg	DISPERSE (takes time) [2,4]	make half [4]<br>can crush and mix with apple puree/ yoghurt [4]	DISPERSE (takes time) [2,4]	make half [4]	the upper GIT [1]<br>suitable to the duodenum, but not to the jejunum [4]	with food GIT side effects ↓ [2,4]	100mg tablets disperse more rapidly [2]<br>Allopurinol Sandoz does not disperse well, if necessary, pre-crush [3]<br>may cause taste disturbances [4]
			tablet 300mg	CRUSH [2,4]	can mix with apple puree/yoghurt [4]	CRUSH [2,4]	-			
M04AA03	**Febuxostat**	Adenuric, Druniler, Febuxostat (KRKA, Mylan, Teva, Zentiva)	film-coated tablet	CRUSH [4]	can mix with apple puree/yoghurt [4]	CRUSH [4]	-	-	regardless of food [4,5]	may cause taste disturbances [4]
M04AC01	**Colchicine**	Colchicina SEID, Colchicum-Dispert	(coated) tablet	DISPERSE (may take time) [2,4]	can crush and mix with apple puree/ yoghurt [4]	DISPERSE (may take time) [2,3,4]	-	-	regardless of food [3,4,5]	-
M05BA04	**Alendronic acid**	Alendronic acid (Accord)	tablet	NO [1,2,4,5]	cannot be chewed/ sucked; high risk of oesophageal damage [1,2,4,5]	DISPERSE [2,3,4]	rinse with at least 50ml of water [3]<br>with caution, if there is a risk of gastric contents returning to the oesophagus [3]	the stomach and upper small intestine [4]<br>probably suitable to the duodenum and jejunum [4]	food ↓ absorption [1,3,4]<br>30min before breakfast, with water [1,3,4,5]	30min after administration (before the first meal) must remain upright [1,3,4,5]<br>may cause severe oesophageal damage[1,4]
M05BB03	**Alendronic acid + colecalciferol**	Alendronic acid/ colecalciferol (Sandoz, Zentiva); Fosavance	tablet	NO [1,4]	cannot be chewed/ sucked, high risk of oesophageal damage [1,4]	NO [4]	-	-	-	**ALTERNATIVE:** administer the APIs as separate medicines [4]

ATC code	Active substance	Medicinal product	Dosage form	Oral administration		Administration by FT		Site of absorption/ administration	Administration with food	Remarks
				Guide	Comment	Guide	Comment			
M05BA06	**Ibandronic acid**	Ibandronic acid (Teva)	film-coated tablet	NO [1,4,5]	cannot be chewed/ sucked; high risk of oesophageal damage [1,4,5]	NO [4]	-	the upper GIT [1,3]	-	**ALTERNATIVE:** change the API [6]
M05BA07	**Risedronc acid**	Norifaz, Risedronate sodium (Accord), Risedros	film-coated tablet	DISPERSE [4]	can disperse with 180ml of water and then thicken to a suitable consistency [4]	DISPERSE [2,3,4]	rinse with at least 50ml of water [3]<br>with caution, if there is a risk of gastric contents returning to the oesophagus [3]<br>FT ≥ CH8 [3]	the stomach and small intestine [3,4]<br>probably suitable to the duodenum and jejunum [3,4]	food ↓ absorption [1,3,4]<br>30min before breakfast, with water [1,3,4,5]	30min after administration (before the first meal) must remain upright [1,3,4,5]<br>may cause severe oesophageal damage[1,4]
M05BX03	**Strontium ranelate**	Osseor, Protelos	granules for oral suspension	YES (look at SmPC) [1]	not suitable if cannot swallow fluids [6]	YES (look at SmPC) [1]	-	-	food ↓ absorption [1]<br>due to slow absorption, it is recommended to take at bedtime and at least 2h after a meal [1]	-
**NERVOUS SYSTEM**										
**ANALGESICS**										
N02AA01	**Morphine**	Sevredol	film-coated tablet	CRUSH [4]	can mix with apple puree/yoghurt [4,6]	CRUSH [4]	-	mostly: the upper small intestine [1]<br>to the jejunum the effect may ↑[4]	administer at the same time regard to food [4]	prolonged formulations are not bioequivalent [6]<br>opening the prolong. capsule does not alter the effect of the drug [6]; if the prolong. tablet's/capsule's contents are crushed, the API is released faster; risk of toxicity [6]<br>may cause dry mouth and taste disturbances [4]
		MXL	prolonged-release capsule (modified granules)	OPEN CAPSULE DON'T CRUSH! [1,2,4]	mix with 30ml of water or juice [4]<br>can mix with apple puree/yoghurt [4]	NO [1,2,3,4,5,6]	does not disperse evenly, the granules stick to the walls of the syringe and FT [6]			
		Vendal retard	prolonged-release tablet	NO [1,4,5]	-		-			
N02AA05	**Oxycodone**	Oxycodone Vitabalans	film-coated tablet	DISPERSE (takes time) [4]	can crush and mix with apple puree/ yoghurt [4]	DISPERSE (takes time) [4]	shake when administering [4]	to the jejunum absorption may ↑ [4]	administer at the same time regard to food [4]	if the prolong. tablet is crushed, the API is released faster, risk of toxicity [6]<br>may cause dry mouth and swallowing difficulties [1,4]
		Oxycontin	prolonged-release tablet	NO [1,4,5]	-	NO [1,3,4]	-			
N02AA55	**Oxycodone + naloxone**	Targinact	prolonged-release tablet	NO [1,4,5]	-	NO [1,3,4]	-	-	-	if the tablet is crushed, the API is released faster, risk of toxicity [6]<br>**ALTERNATIVE:** change the API [6]
N02AA08	**Dihydrocodeine**	DHC Continus	prolonged-release tablet	NO [1,2]	Some tablets are scored [1]	NO [1,2,3]	-	-	-	if the tablet is crushed, the API is released faster, risk of toxicity [6]<br>**ALTERNATIVE:** change the API [6]
N02AB03	**Fentanyl**	Effentora	buccal tablet	SUBLINGUALLY or BUCCALY [1,4]	must not be sucked, chewed or crushed [1,4]<br>saliva necessary [6]	NO [6]	can be administered under the tongue/ in the cheek [2,4,6]<br>risk of aspiration [4,6]	through the oral mucosa faster and more entirely than from the rest of the GIT[1,3,4]	no food at the same time [1,4]	**ALTERNATIVE**: fentanyl transdermal patch [6]
		Lunaldin	sublingual tablet							
N02AJ06	**Codeine + paracetamol**	Algocalm, Paracetamol/ Codeine Vitabalans	tablet	CRUSH [4]	can mix with apple puree/yoghurt [4]	CRUSH [4]	-	see codeine and paracetamol [6]		

ATC code	Active substance	Medicinal product	Dosage form	Oral administration		Administration by FT		Site of absorption/ administration	Administration with food	Remarks
				Guide	Comment	Guide	Comment			
N02AJ13	**Tramadol + paracetamol**	Zaldiar	film-coated tablet	CRUSH [4]	can mix with apple puree/yoghurt [4]	CRUSH [4]	-	see tramadol and paracetamol [6]		
N02AJ14	**Tramadol + dexketoprofen**	Skudexa	granules for oral solution (in bag)	YES<br>(look at SmPC) [1]	not suitable if cannot swallow fluids [6]	NO [6]	-	see tramadol and dexketoprofen [6]	see tramadol and dexketoprofen [6]	see tramadol and dexketoprofen [6]<br>**ALTERNATIVE:** change the API [6]
			film-coated tablet	NO [6]	-					
N02AX02	**Tramadol**	Tramadol (Lannacher)	oral drops	YES [1,2,4]	-	DILUTE [4]	-	to the jejunum absorption may ↑ [4]	regardless of food [3,4,5]	if the prolong. tablet is crushed, the API is released faster, risk of toxicity [6]<br>may cause dry mouth [1]
		Tramadol (Lannacher, Vitabalans)	(film-coated) tablet	CRUSH [6]	can mix with apple puree/yoghurt [4,6]	NO [6]	no info, risk of FT blockage [6]			
		Tramadol (KRKA)	capsule (powder)	OPEN CAPSULE [4]		DISPERSE CAPSULE CONTENTS [3,4]	FT ≥ CH8 [3]			
		Mabron retard, Olteron, Tramadol (KRKA, Lannacher)	prolonged-release tablet	NO [1,4,5]	some tablets are scored [1]	NO [1,3,4]	-			
N02BE01	**Paracetamol**	Efferalgan	effervescent tablet	DISPERSE (50ml) [1,2,4]	-	DISPERSE<br>(in cup, 50ml) [2,3,4]	FT ≥ CH8 [3]	a little the stomach, mostly the small intestine [5]<br>also suitable to the duodenum and jejunum [4]	regardless of food [3,4]	-
			oral solution	YES [1,2,4]	can dilute with water/ milk/juice [1,4,6]<br>not suitable if cannot swallow fluids [4]	DILUTE [3,4]	dilution necessary to ↓ viscosity and osmolarity [2,3]			
		Panadol	oral suspension							
		Paramax (Forte, Rapid)	(film-coated) tablet	DISPERSE<br>(may take time) [6]	can crush and mix with apple puree/ yoghurt [4]	DISPERSE<br>(may take time) [6]	-			
		Panadol Optizorb, Paracetamol (Actavis, Grindeks, Sopharma)		CRUSH [6]		CRUSH [6]				
N02CC01	**Sumatriptan**	Imigran FTD	film-coated tablet	DISPERSE [1,4]	bitter [1]<br>can disperse in 2.5ml of water and mix with apple puree/yoghurt [4]	DISPERSE [4]	-	-	regardless of food [3,4]	-
		Cinie, Sumatriptan (Actavis, STADA)	(coated) tablet	CRUSH [1,4]	-	CRUSH [4]				
**ANTIEPILEPTICS**										
N03AA02	**Phenobarbital**	Phenaemal	tablet	CRUSH [2,4]	can mix with juice/ apple puree/yoghurt [4]	CRUSH [2,4]	-	also suitable to the jejunum[4]	regardless of food [3,4]	-
N03AA03	**Primidone**	Liskantin	tablet	DISPERSE<br>(may take time) [2,4]	bitter [4]<br>can crush and mix with apple puree/ yoghurt [4]	DISPERSE<br>(may take time) [2,3,4]	FT ≥ CH8 [3]	-	regardless of food [3,4,5]	-
N03AB02	**Phenytoin**	Phenhydan	tablet	CRUSH [4]	when mixed with food, absorption can ↓ [4]	NO [2,3]	absorption is unpredictable [2,4]<br>permitted only in cases of emergency [4]	the small intestine [3]<br>not suitable to the jejunum [3,4]	stop ETF 2h before and after [3,4]	**HAZARDOUS TO PREGNANT!** [7]<br>**TDM needed** [2]<br>**ALTERNATIVE:** change the API [6]
N03AE01	**Clonazepam**	Rivotril	tablet	DISPERSE [2,4]	can crush and mix with apple puree/ yoghurt [4]	DISPERSE [2,3,4]	shake before administration [4]<br>rinse!!! [4]<br>FT ≥ CH8 [3]	-	regardless of food [3,4]	**-**

ATC code	Active substance	Medicinal product	Dosage form	Oral administration		Administration by FT		Site of absorption/ administration	Administration with food	Remarks
				Guide	Comment	Guide	Comment			
N03AF01	**Carbamazepine**	Carbalex	tablet	DISPERSE [4]	can crush and mix with apple puree/ yoghurt [4]	DISPERSE [3,4]	shake before administration [4]<br>risk of FT blockage [3,4]	-	with food GIT side effects ↓ [1,3,4]<br>ETF delays absorption, stop ETF 2h before and after [4]	**HAZARDOUS TO PREGNANT!** [7]<br>if the prolong. tablet is crushed, the API is released faster, risk of toxicity [6]<br>**TDM needed** [2]<br>may cause dry mouth [4]
		Carbalex retard, Timonil retard	prolonged-release tablet	DISPERSE [1]	can mix with water/ tea/juice/milk [1]	NO [3,4]	risk of FT blockage [6]			
		Tegretol CR		NO [4,5]	-		-			
N03AF02	**Oxcarbazepine**	Apydan, Oxcarbazepine Teva, Trileptal	(film-coated) tablet	DISPERSE (takes time) [3]	can crush and mix with apple puree/ yoghurt [4,6]	DISPERSE (takes time) [3]	FT ≥ CH8 [3]	also suitable to the duodenum and jejunum [4]	regardless of food [1,3,4]	-
N03AG01	**Sodium valproate**	Convulex	syrup	YES [1,2,4]	not suitable if cannot swallow fluids [4]	DILUTE [3,4]	at high doses, the volume of the solution may be to large [4]	not suitable to the duodenum and jejunum [4]	can delay absorption [3]<br>with food GIT side effects ↓ [4,5]<br>ETF may ↓ absorption, stop ETF 2h before and after [4]	**HAZARDOUS TO PREGNANT!** [1,7]<br>opening the prolong. capsule does not alter the effect of the drug [6]; if the prolonged or gastro-resistant tablet's/ granule's/capsule's contents are crushed, the API release is altered; the effect of the drug is unpredictable [6]<br>the oral solution can be administered rectally [4]<br>**TDM needed** [2]
		Orfiril Saft	oral solution							
		Orfiril long	prolonged-release granules	DISPERSE DON'T CRUSH! [1]	the granules can be administered directly into the mouth [1]<br>can mix with yoghurt [1]	DISPERSE DON'T CRUSH! [1,3]	FT ≥ CH9 [3]			
		Orfiril long	prolonged-release capsule (modified granules)	OPEN CAPSULE DON'T CRUSH! [1]		NO [1,2,3,4]	-			
		Orfiril	gastro-resistant tablet	NO [1,2,3,4,5]	-					
	**Sodium valproate + valproic acid**	Depakine Chrono, Convulex retard, Valproate sodium (Sandoz)	prolonged-release tablet		some tablets are scored [1]					
	**Valproic acid**	Convulex	gastro-resistant soft capsule		-					
N03AX09	**Lamotrigine**	Lamictal	chewable/ dispersible tablet	CHEW or DISPERSE [1,2,4,5]	can disperse in juice [4,5] OR 2.5ml of water and mix with apple puree/yoghurt [4]	DISPERSE [2,3,4]	FT ≥ CH8 [3]	probably the whole GIT [6]	regardless of food [1,3,4]	the resulting suspension can be administered rectally [4]<br>**TDM needed** [2]
N03AX11	**Topiramate**	Topamax, Topiramate (Elvim, Orion)	film-coated tablet	DISPERSE (takes time) [2,4]	bitter [2,4,5]<br>can crush and mix with apple puree/ yoghurt [4]	DISPERSE (takes time) [2,3,4]	min 2.5ml [4]<br>FT ≥ CH8 [3]	also suitable to the duodenum and jejunum [4]	regardless of food [1,3,4,5]	**HAZARDOUS TO PREGNANT!** [7]<br>the resulting suspension can be administered rectally [4]
N03AX12	**Gabapentin**	Gabagamma, Gabapentin (Accord), Neurontin	capsule (powder)	OPEN CAPSULE [2,4,5]	bitter [2,4]<br>can mix with juice/ apple puree/yoghurt [4,5]	DISPERSE CAPSULE CONTENTS [2,3,4]	FT ≥ CH8 [3]	the upper small intestine [3,5]<br>also suitable to the duodenum, but not to the jejunum [4]	regardless of food [1,3,4,5]	hydrolyses, administer immediately [2]<br>may cause dry mouth [4]
N03AX14	**Levetiracetam**	Keppra, Levetiracetam (Accord, Teva), Normeg, Matever	film-coated tablet	CRUSH [2,4]	bitter [1,2,5]<br>can mix with apple puree/yoghurt [4,5]	CRUSH [2,4,5]	FT ≥ CH8 [3]	also suitable to the duodenum and jejunum [4]	regardless of food [1,3,4,5]	-
N03AX15	**Zonisamide**	Zonilamide (Sandoz)	capsule	OPEN CAPSULE [2,4]	can mix with apple puree/yoghurt [4]	DISPERSE CAPSULE CONTENTS [2,3,4]	-	-	regardless of food [1,3,4,5]	**-**
N03AX16	**Pregabalin**	Brieka, Lyrica, Pragiola, Pregabalin (Accord, Zentiva k.s.), Siranalen	capsule (powder)	OPEN CAPSULE [2,4]	bad taste [2]<br>mix with 120ml of water [4]<br>can mix with apple puree/yoghurt [4]	DISPERSE CAPSULE CONTENTS [2,3,4]	FT ≥ CH8 [3]	also suitable to the duodenum and jejunum [4]	regardless of food [1,3,4,5]	-

ATC code	Active substance	Medicinal product	Dosage form	Oral administration		Administration by FT		Site of absorption/ administration	Administration with food	Remarks
				Guide	Comment	Guide	Comment			
N03AX17	**Stiripentol**	Diacomit	capsule	NO [1]	-	NO [1]	-	-	-	**ALTERNATIVE:** change the API [6]
N03AX22	**Perampanel**	Fycompa	film-coated tablet	DISPERSE [4]	can crush and mix with apple puree/ yoghurt [4]	DISPERSE [4]	-	-	regardless of food [1,4,5]	-
N03AX23	**Brivaracetam**	Briviact	film-coated tablet	CRUSH [4]	can mix with apple puree/yoghurt [4]	CRUSH [4]	-	-	regardless of food [1,4,5]	-
**ANTI-PARKINSON DRUGS**										
N04AA01	**Trihexyphenidyl**	Cyclodol	tablet	DISPERSE [2,4]	can mix with yoghurt but not with juice/acidic foods [4]	DISPERSE [2,3,4]	-	-	regardless of food [1,3,4]<br>with food GIT side effects ↓[5]	may cause dry mouth [4,5]
N04BA03	**Levodopa + carbidopa + entacapone**	Levodopa/Carbidopa/ Entacapone (Teva)	film-coated tablet	DISPERSE (40ml; 15min) [6]	bitter [2]	DISPERSE (40ml; 15min) [6]	may stain the FT [6]<br>FT ≥ CH10 [6]	to the upper small intestine [4]<br>to the jejunum absorption rate may ↑ [4]	food ↓ levodopa absorption [4]<br>with food GIT side effects ↓[5]<br>administer at the same time regard to food [4]<br>in case of Parkinson's, administer 30min before or 1h after a meal [1,4]	opening the prolong. capsule does not alter the effect of the drug [6]; if the capsule's contents are crushed, the API is released faster; risk of toxicity [6]<br>may cause dry mouth [4]
		Stalevo		CRUSH [2]	bitter [2]<br>hard to crush [2,4]	CRUSH [2]	hard to crush [2,4]			
N04BA80	**Levodopa + benserazide**	Madopar	dispersible tablet	DISPERSE (25-50ml) [1,4]	-	DISPERSE (25ml) [4]	FT ≥ CH8 [3]			
			capsule	OPEN CAPSULE [4]	can mix with apple puree [4]	DISPERSE CAPSULE CONTENTS [4]	-			
			tablet	DISPERSE (takes time) [4]	can crush and mix with apple puree [4]	DISPERSE (takes time) [4]				
		Madopar HBS	prolonged-release capsule	NO [1,2,4,5]	-	NO [1,2,3,4]				
N04BA81	**Levodopa + carbidopa**	Sinemet	tablet	CRUSH [4]	-	CRUSH [4]	FT ≥ CH8 [3]			
		DuoDopa	intestinal gel	NO [1,4]	intended for intestinal administration [6]	YES (look at SmPC) [1,3,4]	-			
N04BB01	**Amantadine**	Amantadin-Ratiopharm	film-coated tablet	NO [6]	-	NO [6]	-	-	-	may cause dry mouth [4]
N04BC04	**Ropinirole**	Ropinirole Orion	film-coated tablet	DISPERSE [4]	disperse with 2.5ml of water and mix with apple puree/yoghurt [2,4]	DISPERSE [3,4]	min 2.5ml [4]<br>FT ≥ CH8 [3]	-	with food GIT side effects ↓ [1,3]	if the prolong. tablet is crushed, the API is released faster; risk of toxicity [6]<br>may cause dry mouth [4]
		Requip-Modutap, Rolpryna	prolonged-release tablet	NO [1,5]	-	NO [1,3]	-			
N04BC05	**Pramipexole**	Ezaprev, Mirapexin	tablet	DISPERSE (takes time) [4]	can crush and mix with apple puree/ yoghurt [4]	DISPERSE (takes time) [4]	-	-	regardless of food [1,4]<br>with food GIT side effects ↓[5]	if the prolong. tablet is crushed, the API is released faster; risk of toxicity [6]<br>may cause dry mouth [4]
		Mirapexin, Oprymea	prolonged-release tablet	NO [1,4,5]	-	NO [1,4]				
N04BD02	**Rasagiline**	Azilect, Ralago, Sagilia	tablet	DISPERSE [4]	can crush and mix with apple puree/ yoghurt [4]	DISPERSE [4]	-	-	regardless of food [1,4,5]	may cause dry mouth [4]

ATC code	Active substance	Medicinal product	Dosage form	Oral administration		Administration by FT		Site of absorption/ administration	Administration with food	Remarks
				Guide	Comment	Guide	Comment			
**PSYHOLEPTICS**										
**ANTIPSYCHOTICS**										
N05AA01	**Chlorpromazine**	Aminazin Zdorovje, Chlorpromazine Sterop, Klorproman	tablet	DISPERSE (30min) [6]	the dispersed drug can irritate the oral mucosa [4]	NO [2,4]	risk of FT blockage [6]	-	food may ↓ absorption [4]<br>with food GIT side effects ↓[5]	risk of contact dermatitis if crushed [2,3,4]<br>may cause dry mouth [4]
N05AA02	**Levome-promazine**	Levomepromazin Orion	tablet	DISPERSE [2]	-	DISPERSE [2,3]	FT ≥ CH8 [3]	-	regardless of food [1,3]	very light-sensitive, administer immediately!; do not use if the solution is pink or yellow [4]
N05AD01	**Haloperidol**	Haloperidol Richter	oral drops	YES [1]	can mix with acidic drinks [4]	DILUTE [2,3,4]	-	-	regardless of food [3,4]	may cause dry mouth or increase of saliva, swallowing difficulties and risk of aspiration [4]
			tablet	DISPERSE (takes time) [4]	can mix with apple puree/yoghurt [4]	DISPERSE (takes time) [4]				
N05AD03	**Melperone**	Buronil	film-coated tablet	NO [6]	no info [6]	NO [6]	no info [6]	-	-	**ALTERNATIVE:** change the API [6]
N05AE03	**Sertindole**	Serdolect	film-coated tablet	NO [6]	no info [6]	NO [6]	no info [6]	-	-	**ALTERNATIVE:** change the API [6]
N05AF01	**Flupentixol**	Fluanxol	film-coated tablet	CRUSH [2]	mix with fruit juice [2]	CRUSH [2]	FT ≥ CH8 [3]	-	regardless of food [3,5]	-
N05AF03	**Chlorprothixene**	Truxal	film-coated tablet	NO [6]	no info [6]	NO [6]	no info [6]	-	-	**ALTERNATIVE:** change the API [6]
N05AF05	**Zuclopenthixol**	Cisordinol	film-coated tablet	CRUSH [2,4]	can mix with apple puree/yoghurt [4]	CRUSH [2,4]	FT ≥ CH6 [3]	-	regardless of food [1,3,4,5]	may cause dry mouth or increase of saliva [4]
N05AH02	**Clozapine**	Leponex	tablet	CRUSH [2,4]	can mix with apple puree/yoghurt [4]	CRUSH [2,4]	-	-	regardless of food [1,3,4,5]	not recommended [2,3,4]<br>may cause dry mouth, swallowing difficulties and risk of aspiration [4]
N05AH03	**Olanzapine**	Farpenta, Olanzapine (Actavis, Teva), Zalasta	orodispersible tablet	DISPERSE IN MOUTH [1,2]	can disperse in water/ juice/milk [1,2,3]	DISPERSE [2,3,4]	-	-	regardless of food [1,3,4,5]	may cause dry mouth, swallowing difficulties and risk of aspiration [4]
		Olansapin (Actavis), Olansapine (Accord), Zalasta	(film-coated) tablet	DISPERSE (takes time) [4]	can mix with apple puree/yoghurt [4]	DISPERSE (takes time) [4]				
N05AH04	**Quetiapine**	Ketipinor, Kventiax, Quetiapine (Teva), Seroquel	film-coated tablet	CRUSH [2,4]	bitter [2]<br>can mix with apple puree/yoghurt [4]	CRUSH [2,4,5]	-	-	regardless of food [4,5]	if the prolong. tablet is crushed, the API is released faster; risk of toxicity [6]<br>may cause dry mouth, swallowing difficulties and risk of aspiration [4]
		Kventiax SR, Quetiapin (Accord, Teva)	prolonged-release tablet	NO [4,5]	-	NO [4]				
N05AL01	**Sulpiride**	Betamaks	(film-coated) tablet	DISPERSE (may take time) [2]	-	DISPERSE (may take time) [2,3]	FT ≥ CH8 [3]	-	regardless of food [3]	-

ATC code	Active substance	Medicinal product	Dosage form	Oral administration		Administration by FT		Site of absorption/ administration	Administration with food	Remarks
				Guide	Comment	Guide	Comment			
N05AL05	**Amisulpiride**	Amisan, Solian	(film-coated) tablet	CRUSH [4]	can mix with apple puree/yoghurt [4]	CRUSH [4]	-	-	regardless of food [1,3,4]	may cause dry mouth [4]
N05AN01	**Lithium**	Lithium Slovakopharma, Lito	tablet	CRUSH [4]	can mix with apple puree/yoghurt [4]	CRUSH [4]	-	the jejunum and ileum [3,4] also suitable to the duodenum and jejunum [2]	with food GIT side effects ↓ [4,5]	**TDM needed** [2] may reduce appetite [2], and cause dry mouth and taste disturbances [4]
N05AX08	**Risperidone**	Rispolept	oral solution	YES [1,2,4,5]	can dilute with orange juice [5] not suitable if cannot swallow fluids [4]	DILUTE [2,3,4]	-	absorption may ↓ if administered by FT[4]	regardless of food [1,3,4,5]	may cause dry mouth or increase of saliva, swallowing difficulties and risk of aspiration [4]
		Medorisper, Rispolept, Risperidon (Actavis)	film-coated tablet	DISPERSE [4]	can disperse with 2.5ml of water and mix with apple puree/ yoghurt [4]	DISPERSE [4]	min 2.5ml [4]			
N05AX12	**Aripiprazole**	Abilify, Aripiprazole (Accord, Teva, Zentiva), Arisppa, Zycalor	tablet	DISPERSE (10min) [4,6]	can crush and mix with apple puree/ yoghurt [4]	DISPERSE (10min) [4]	-	-	regardless of food [4,5]	may cause dry mouth, swallowing difficulties and risk of aspiration [4]
N05AX15	**Clariprazine**	Reagila	capsule (powder)	OPEN CAPSULE [6]	-	NO [6]	no info [6]	-	regardless of food [1,5]	administer every day at the same time [1] **ALTERNATIVE:** change the API [6]
**ANXIOLYTICS**										
N05BA01	**Diazepam**	Diazepam-Valocordin	oral drops	YES [1,5]	can mix with water/ juice/apple puree [5]	DILUTE [2,3,4]	-	-	regardless of food [3,4]	the oral solution can be administered p/r [4] may cause dry mouth or increase of saliva [4] when the solution is mixed with water, a white precipitate forms (the solution becomes milky), which does not affect the oral effect [6]
		Diazepeks	tablet	DISPERSE (takes time) [4]	can crush and mix with apple puree/ yoghurt [4]	DISPERSE (takes time) [3,4]	prefer when administering to the jejunum [3,4] FT ≥ CH8 [3]			
N05BA06	**Lorazepam**	Lorans	tablet	DISPERSE [2]	can crush and mix with apple puree/ yoghurt [4]	DISPERSE [2,3,4]	-	also suitable to the duodenum and jejunum [4]	regardless of food [1,3,4]	Can be administered sublingually [2,3,4,5] the solution for injection may be administered rectally [5] may cause dry mouth or increase of saliva [4]
		-	injection solution	SUBLINGUALLY [3]	-	NO [6]	can be administered under the tongue [5] risk of aspiration [6]			
N05BA08	**Bromazepam**	Bromazepam Lannacher, Lexotanil	(film-coated) tablet	DISPERSE [2,4]	can crush and mix with apple puree/ yoghurt [4]	DISPERSE [2,4]	-	absorbed by gastric FT [4]	regardless of food [1,4,5]	may cause dry mouth [4]
N05BA12	**Alprazolam**	Alprazolam (Grindex), Xanax	tablet	DISPERSE [2,4]	can crush and mix with apple puree/ yoghurt [4]	DISPERSE [2,4]	-	-	regardless of food [4]	the tablet can be administered sublingually [4,5] if the prolong. tablet is crushed, the API is released faster; risk of toxicity [6]
		Xanax XR	prolonged-release tablet	NO [1,5]	-	NO [1]				
**HYPNOTICS AND SEDATIVES**										
N05CD02	**Nitrazepam**	Nitrazepam (Accord, GSK), Nitrazepams	tablet	DISPERSE [4,5]	can crush and mix with apple puree/ yoghurt [4,5]	DISPERSE [4]	-	-	regardless of food [3,4]	may cause increase of saliva [4]

ATC code	Active substance	Medicinal product	Dosage form	Oral administration		Administration by FT		Site of absorption/ administration	Administration with food	Remarks
				Guide	Comment	Guide	Comment			
N05BA12	**Midazolam**	Dormicum	film-coated tablet	CRUSH [6]	-	NO [6]	no info [6]	-	regardless of food [3]	the solution for injection and infusion can be administered rectally [1,3,5]
		Midazolam (Accord)	injection/infusion solution	DILUTE [3,5,6]	can mix with juice [3] OR glucose solution [6]	DILUTE [3,6]	-			
N05CF01	**Zopiclone**	Imovane, Somnols, Zopitin	film-coated tablet	DISPERSE (may take time) [4]	bitter [3,4] can mix with apple puree/yoghurt [4]	DISPERSE (may take time) [4]	-	-	regardless of food [1,3,4]	may cause dry mouth [4]
N05CF02	**Zolpidem**	Hypnogen, Stilnox, Zolpidem Sandoz, Zolsana	film-coated tablet	DISPERSE [4]	can mix with apple puree/yoghurt [4]	DISPERSE [3,4]	FT ≥ CH8 [3]	-	may delay effect [3,4,5]	may cause dry mouth [4]
N05CH01	**Melatonin**	Melatonin (Pharma Nord, Vitabalans)	film-coated tablet	CRUSH [6]	-	NO [6]	no info, risk of FT blockage [6]	-	-	if the prolong. tablet is crushed, the prolonging effect is lost and the API is released faster [4] may cause dry mouth and mouth ulcers[4]
		Apotheka Melatoniin	capsule	OPEN CAPSULE [2]		DISPERSE CAPSULE CONTENTS [2]	-			
		Circadin, Slenyto	prolonged-release tablet	CRUSH [4]	-	NO [1,6]	risk of FT blockage [6]			
**PSYCHOANALEPTICS**										
**ANTIDEPRESSANTS**										
N06AA04	**Clomipramine**	Anafranil	coated tablet	CRUSH [4]	numbing [4] can mix with apple puree/yoghurt [4]	CRUSH [4]	check for large particles [4]	to the jejunum absorption may ↑; risk of toxicity ↑ [4]	regardless of food [1,3,4]	if the prolong. tablet is crushed, the API is released faster; risk of toxicity [6] may cause dry mouth and taste disturbances [4]
		Anafranil retard	prolonged-release tablet	NO [1,2]	some tablets are scored [1]	NO [1,2,3]	-			
N06AA09	**Amitriptyline**	Amitriptylin (Grindex, Nycomed)	film-coated tablet	CRUSH [4]	bitter [2,4] and numbing [4] can mix with apple puree/yoghurt [4]	CRUSH [4]	-	to the jejunum absorption may ↑; risk of toxicity ↑ [4]	regardless of food [3,4]	can be administered buccally, but the taste may be unacceptable [3,4] may cause dry mouth and taste disturbances [4]
N06AA10	**Nortriptyline**	Noritren	film-coated tablet	DISPERSE [2,4]	can crush and mix with apple puree/ yoghurt [4]	DISPERSE [2,4]	-	to the jejunum absorption may ↑; risk of toxicity ↑ [4]	regardless of food [4,5]	may cause dry mouth [4]
N06AB03	**Fluoxetine**	Fluoxetine Vitabalans	film-coated tablet	DISPERSE [6]	can crush and mix with apple puree/ yoghurt [4,6]	DISPERSE [6]	FT ≥ CH10 [6]	-	regardless of food [1,3,4,5]	may reduce appetite [2], cause dry mouth, taste disturbances and swallowing difficulties [4]
		Flux	capsule	OPEN CAPSULE [2,4]	can mix with apple puree/yoghurt [4]	DISPERSE CAPSULE CONTENTS [2,3,4]	-			
N06AB04	**Citalopram**	Cipramil, Ciral, Citalec, Citalopram (Actavis, Teva)	film-coated tablet	CRUSH [2,4]	bad taste [2] can mix with apple puree/yoghurt [4]	CRUSH [2,4]	FT ≥ CH8 [3]	the small intestine [2]	regardless of food [3,4]	may cause dry mouth [4]
N06AB05	**Paroxetine**	Arketis, Paroxetin (Actavis, Hexal), Seroxat	(film-coated) tablet	CRUSH [2,4]	cannot be chewed [1] bitter[1,2] and numbing[1,2] can mix with apple puree/yoghurt [4]	CRUSH [2,3,4]	-	also suitable to the duodenum and jejunum [4]	regardless of food [3,4] with food GIT side effects ↓ [1,4]	may cause dry mouth [4]
N06AB06	**Sertraline**	Asentra, Erset, Sertralin (Accord), Sertraline (Lannacher, Teva), Zoloft	film-coated tablet	DISPERSE [4]	bitter [2] and numbing [2] can crush and mix with apple puree/ yoghurt [2,4]	DISPERSE [2,3,4]	shake before administration [4] FT ≥ CH8 [3]	-	regardless of food [3,4]	may cause dry mouth [4]

ATC code	Active substance	Medicinal product	Dosage form	Oral administration		Administration by FT		Site of absorption/ administration	Administration with food	Remarks
				Guide	Comment	Guide	Comment			
N06AB08	**Fluvoxamine**	Fevarin	film-coated tablet	CRUSH [2,4]	can mix with apple puree/yoghurt [4]	CRUSH [2,3,4]	check for large particles [4]	-	regardless of food [1,3,4,5]	may cause dry mouth and taste disturbances [4]
N06AB10	**Escitalopram**	Elicea Q-Tab	orodispersible tablet	DISPERSE IN MOUTH[1]	-	DISPERSE [6]	check for large particles [6]	-	regardless of food [1,3,4,5]	may cause dry mouth and taste disturbances [4]
		Cipralex, Ciraset, Elicea, Escitalopram (Teva, Actavis, Accord), Eslorex, Estan	film-coated tablet	DISPERSE (may take time) [2,4]	bitter [2,4] can crush and mix with apple puree/ yoghurt [4]	DISPERSE (may take time) [2,4]	FT ≥ CH10 [6]			
N05AX05	**Trazodone**	Trarett	prolonged-release tablet	NO [1,5]	can be divided into three smaller parts [1]	NO [1]	-	-	-	if the prolong. tablet is crushed, the API is released faster; risk of toxicity [6] **ALTERNATIVE:** change the API [6]
N06AX11	**Mirtazapine**	Mirzaten Q-Tab, Mirtazapin (Actavis, Orion)	orodispersible tablet	DISPERSE IN MOUTH [1]	prefer [2]	DISPERSE [2,4]	-	mostly: the duodenum [2,4] to the jejunum absorption may ↓ [2,4]	regardless of food [3,4,5]	may cause dry mouth [4]
		Esprital, Mirtastad	film-coated tablet	CRUSH [4]	bitter [2] and numbing [4] can mix with apple puree/yoghurt [4]	CRUSH [2,3,4]	shake before administration [2]			
N06AX12	**Bupropione**	Elontril	modified-release tablet	NO [1,5]	-	NO [1]	-	-	-	if the modified tablet is crushed, the API is released faster; risk of toxicity [6] **ALTERNATIVE:** change the API [6]
N06AX14	**Tianeptine**	Coaxil, Salymbra	film-coated/coated tablet	CRUSH [6]	-	CRUSH [6]	FT ≥ CH8 [6]	-	with food GIT side effects ↓[1]	-
N06AX16	**Venlafaxine**	Alventa	prolonged-release capsule (modified granules)	OPEN CAPSULE DON'T CRUSH! [2,4,5]	can mix with apple puree/yoghurt [2,4,5]	DISPERSE CAPSULE CONTENTS DON'T CRUSH! [2,4]	shake the syringe constantly when administering; rinse 1-2 times with a total of 50ml of water [6] FT ≥ CH12 [6]	probably also suitable to the jejunum [3]	regardless of food [3] with food GIT side effects ↓ [1,4]	opening the prolong. capsule does not alter the effect of the drug [6]; if the prolong. tablet's/capsule's contents are crushed, the API is released faster; risk of toxicity [6] may cause dry mouth and taste disturbances [4]
		Efexor XR, Venlafaxin (Medochemie)	prolonged-release capsule (modified spheroids)			NO [1,2,3,4]	-			
		Venlafaxine Elvim, Venlagamma	prolonged-release capsule (modified minitablets)		if the patient can swallow mini-tablets [6]					
		Argofan SR	prolonged-release tablet	NO [1,2,4,5]	-					
N06AX21	**Duloxetine**	Cymbalta, Duloxetin (Zentiva), Duloxgamma, Dulsevia, Onelar	gastro-resistant capsule (modified pellets)	OPEN CAPSULE DON'T CRUSH! [2,4,5]	do not mix with water; the gastro-resistant coating dissolves [3,4] can mix with apple juice/puree/yoghurt [4,5]	NO [1,3,4]	when dispersed with water, the gastro-resistant coating dissolves [3,4] risk of FT blockage [3]	gastro-resistant granules release the API in the distal duodenum (pH≥5.5) [1]	regardless of food [3,4,5]	If the gastro-resistant coating is crushed or dissolved, the API degrades in stomach acid [6] may cause dry mouth, taste disturbances and swallowing difficulties[4] **ALTERNATIVE:** change the API [6]
N06AX22	**Agomelatine**	Agomelatin (Mylan, Teva), Lamegom, Zilbea, Valdoxan	film-coated tablet	CRUSH [4]	can mix with apple puree/yoghurt [4]	CRUSH [4]	-	-	regardless of food [1,4]	-

ATC code	Active substance	Medicinal product	Dosage form	Oral administration		Administration by FT		Site of absorption/ administration	Administration with food	Remarks
				Guide	Comment	Guide	Comment			
N06AX26	**Vortioxetine**	Brintellix	film-coated tablet	CRUSH [4]	can mix with apple puree/yoghurt [4]	CRUSH [4]	-	-	regardless of food [1,4,5]	-
**PSYCHOSTIMULANTS, AGENTS USED FOR ADHD AND NOOTROPICS**										
N06BA04	**Metylphenidate**	Medikinet	tablet	DISPERSE (takes time) [4]	can crush and mix with apple puree/ yoghurt [4]	DISPERSE (takes time) [4]	-	-	administer at the same time as food [4]<br>can modify the dosage form regardless of food [4,5]	opening the modified capsule does not alter the effect of the drug [6]; if the prolong. tablet's/modified capsule's contents are crushed, the API is released faster; risk of toxicity [6]<br>may cause dry mouth [4]
		Medikinet XL	modified-release capsule (pellets + modified pellets)	OPEN CAPSULE; DON'T CRUSH! [1,5]	can mix with apple puree/yoghurt [1,5]	NO [1,2,4]	risk of FT blockage [2,4]			
		Concerta	prolonged-release tablet	NO [1,2,4,5]	-		-			
N06BA09	**Atomoxetine**	Atominex	capsule (powder)	OPEN CAPSULE [4]	bitter [4]<br>can mix with apple puree/yoghurt [4]	DISPERSE CAPSULE CONTENTS [4]	-	-	regardless of food [1,4,5]	irritation if contact with the eyes [5]<br>may cause dry mouth [4]
N06BX03	**Piracetam**	Nootorpil	film-coated tablet	NO [6]	-	NO [6]	-	-	-	**ALTERNATIVE:** change the API [6]
**ANTI-DEMENTIA DRUGS**										
N06DA02	**Donepezil**	Donepezil (Actavis)	film-coated tablet	DISPERSE (may take time) [4]	bitter [4]<br>can crush and mix with apple puree/ yoghurt [4]	DISPERSE (may take time) [4]	FT ≥ CH8 [3]	the small intestine [3]	regardless of food [1,3,4,5]	-
N06DX01	**Memantine**	Axura, Mantomed, Marixino, Memantine (Accord, Grindeks, Ratiopharm)	film-coated tablet	DISPERSE (may take time) [4]	bitter [4]<br>can crush and mix with apple puree/ yoghurt [4]	DISPERSE (may take time) [4]	shake before administration [2,4]	-	regardless of food [1,3,4,5]	administer every day at the same time [1]
**OTHER NERVOUS SYSTEM DRUGS**										
N07AA02	**Pyridostigmine**	Kalymin, Mestinon	(coated) tablet	CRUSH [2,4]	can mix with apple puree/yoghurt [4]	CRUSH [2,3,4]	-	mostly: the duodenum [4]<br>to the jejunum absorption may ↓ [4]	regardless of food [3,4]	risk of contact dermatitis if crushed [4]<br>difficulties with chewing and swallowing may indicate a toxic effect of the drug [4]
N07BA01	**Nicotine**	Nicorette (Coolberry, Coolmint	oromucosal spray	SUBLINGUALLY or BUCCALLY [1,4]	-	NO [6]	can be administered under the tongue/in the cheek [4,6]	the oral mucosa [1,4]	no food during administration [1,4]<br>it is not recommended to take acidic drinks and food 15min before, absorption may ↓ [1,4]	**ALTERNATIVE:** transdermal patch [4,6]
		Niquitin mini	compressed lozenge	DISSOLVE IN MOUTH [1,4]	cannot be chewed [1,4]<br>cannot be swallowed [4,6]	NO [6]	-			
		Nicorette (Freshfruit), Niquitin mint	chewing-gum	CHEW (look at SmPC) [1,4]	cannot be swallowed [4,6]					
N07BB05	**Nalmefene**	Selincro	film-coated tablet	NO [1]	-	NO [1]	-	-	-	should not be halved or crushed as direct skin contact may cause skin sensitisation [1]<br>**ALTERNATIVE:** change the API [6]

ATC code	Active substance	Medicinal product	Dosage form	Oral administration		Administration by FT		Site of absorption/ administration	Administration with food	Remarks
				Guide	Comment	Guide	Comment			
N07BC02	**Methadone**	Metadon DAK	oral solution	YES [1,4]	not suitable if cannot swallow fluids [6]	DILUTE [3,4]	-	-	regardless of food [3,4]	may cause dry mouth [4]
			tablet	DISPERSE (may take time) [4]	can crush and mix with apple puree/ yoghurt [4]	DISPERSE (may take time) [4]				
N07CA01	**Betahistine**	Betaserc	orodispersible tablet	DISPERSE IN MOUTH [1]	-	DISPERSE [6]	check for large particles [6]	-	with food GIT side effects ↓ [3,4,5]	-
		Betaserc, Betahistine (Accord, Actavis), Vertimed	tablet	CRUSH [2,4]	can mix with apple puree/yoghurt [4]	CRUSH [2,3,4]	FT ≥ CH8 [3]			
N07CA02	**Cinnarizine**	Cinnaron	capsule	OPEN CAPSULE [2]	-	DISPERSE CAPSULE CONTENTS [2]	-	-	preferably after a meal [1,3,4]	-
		Stugeron	tablet	DISPERSE (may take time) [2]		DISPERSE (may take time) [2,3]	FT ≥ CH8 [3]			
N07XX02	**Riluzole**	Rilutek	film-coated tablet	CRUSH [2,4]	numbing [2]<br>can mix with apple puree/yoghurt [2,4]	CRUSH [2,3,4]	rinse [2]	-	high-fat food ↓ absorption [1,2,3,4,5]<br>stop ETF 1h before and 2h after [5]	may cause dry mouth and numbness in the mouth [4]
**ANTIPARASITIC PRODUCTS, INSECTICIDES AND REPELLENTS**										
P01AB02	**Tinidazole**	Tinidazol Polpharma	tablet	CRUSH [2]	bitter [2]<br>can mix with apple puree/yoghurt [2]	CRUSH [2]	-	-	with food GIT side effects ↓ [5]	-
P01BA02	**Hydroxy-chloroquine**	Plaquenil	film-coated tablet	CRUSH [2,4,5]	bitter [4,5]<br>can mix with apple puree/yoghurt [4,5]	CRUSH [2,4]	-	-	with food GIT side effects ↓ [4,5]	-
P01BC02	**Mefloquine**	Lariam	tablet	CRUSH [2,4,5]	bitter [4,5]<br>can mix with water/ milk/apple puree/ yoghurt [4,5]	CRUSH [4]	-	-	regardless of food [4]	the absorption of the suspension from the crushed tablet may be higher than from the whole tablet [5]
P02CA01	**Mebendazole**	Vermox, Mebendazole Grindex	tablet	CHEW or CRUSH [1,4,5]	can mix with apple puree/yoghurt [4,5]	DISPERSE (may take time) [4]	-	low absorption [1,5]	food ↑ bioavailability; for intestinal infections on an empty stomach, for systemic infections on an empty stomach [4]	-
P02CA03	**Albendazole**	Albendazole	chewable tablet	CHEW or CRUSH [1,2,4]	mix with water [4]	CRUSH [4]	-	low absorption [1,5]	food ↑ bioavailability; for intestinal infections on an empty stomach, for systemic infections on an empty stomach [4]	-
		Albendazole	tablet	CHEW or CRUSH [2,5,6]		DISPERSE (takes time) [6]	FT ≥ CH8 [6]			
		Albenza, Eskazole	(coated) tablet			NO [6]	no info, risk of FT blockage [6]			

ATC code	Active substance	Medicinal product	Dosage form	Oral administration		Administration by FT		Site of absorption/ administration	Administration with food	Remarks
				Guide	Comment	Guide	Comment			
**RESPIRATORY SYSTEM**										
**NASAL PREPARATIONS**										
R01BA02	**Pseudo-ephedrine**	Sudafed	syrup	YES [1,2]	not suitable if cannot swallow fluids [6]	DILUTE [2]	-			
			film-coated tablet	CRUSH [4]	bad taste [2]<br>can mix with apple puree/yoghurt [4]	CRUSH [4]				
R01BA81	**Pseudo-ephedrine + triprolidine**	Actifed	syrup	YES [1,4,6]	not suitable if cannot swallow fluids [6]	DILUTE [6]	-			
			film-coated tablet	NO [6]	-	NO [1,6]				
R01BA84	**Pseudo-ephedrine + guaifenesin**	Sudafed Expectorant	syrup	YES [1,4,6]	not suitable if cannot swallow fluids [6]	DILUTE [6]	-	-	regardless of food [4,5]<br>stop ETF 1h before and1h after [2]	if the prolong. tablet is crushed, the API is released faster, risk of toxicity [6]<br>prefer topical drugs [4]
R01BA89	**Pseudo-ephedrine + loratadine**	Clarinase	prolonged-release tablet	NO [1,4,5]	-	NO [1,4]	-			
R01BA93	**Pseudo-ephedrine + cetirizine**	Cirrus	prolonged-release tablet	NO [1,4,5]	-	NO [1,4]	-			
**DRUGS FOR OBSTRUCTIVE AIRWAY DISEASES**										
R03CC02	**Salbutamol**	Ventolin syrup	syrup	YES [1,2]	not suitable if cannot swallow fluids [6]	DILUTE [2]	-	-	-	-
R03DA04	**Theophylline**	Retafyllin retard	prolonged-release tablet	NO [1,2,4,5]	-	NO [1,4]	-	probably also suitable to the jejunum [4]	-	if the prolonged-release tablet is crushed, the API is released faster, risk of toxicity [6]<br>**ALTERNATIVE:** p.o aminophylline solution for injection (NB! check doses and dosing interval)
R03DA05	**Aminophylline**	-	injection solution	DILUTE [2,3]	-	DILUTE [2,3]	-	probably also suitable to the jejunum [4]	administer at the same time as food [4]<br>food ↓ absorption [4]<br>with food GIT side effects ↓[4]	exchanging oral theophylline with oral aminophylline, multiply the daily dose of theophylline by 1.25 and divide it into four doses, as aminophylline is short-acting [6]
R03DC03	**Montelukast**	Monkasta, Montelukast (Accord, Teva)	chewable tablet	CHEW or DISPERSE [2]	can crush and mix with apple puree/ yoghurt [4]	DISPERSE [2]	-	-	regardless of food [3,4,5]	chewable tablets and coated tablets are not bioequivalent [4]
		Monkasta, Montelukast (Teva)	film-coated tablet	DISPERSE (takes time) [4]		DISPERSE (takes time) [3,4]	FT ≥ CH8 [3]			
R03DX07	**Roflumilast**	Doxas	(film-coated) tablet	NO [1]	-	NO [1]	-	-	regardless of food [1]	**ALTERNATIVE:** change the API [6]

ATC code	Active substance	Medicinal product	Dosage form	Oral administration		Administration by FT		Site of absorption/ administration	Administration with food	Remarks
				Guide	Comment	Guide	Comment			
**COUGH AND COLD PREPARATIONS**										
R05CB01	**Acetylcysteine**	ACC	oral solution	YES (look at SmPC) [1,5]	not suitable if cannot swallow fluids [6]	YES (look at SmPC) [2,5]	-	-	with food GIT side effects ↓ [4]	-
		ACC	powder for oral solution							
		ACC, Mucovit, Bronchostad	effervescent tablet				dissolve in a cup [2]			
		-	injection solution (pH=6-7.5)	DILUTE (1:4) [2,3]	bitter [2,3]<br>mix with orange juice to hide the taste [2]	DILUTE (1:4) [2,3]	-			
R05CB02	**Bromhexine**	Bromehexine-Grindeks	syrup	YES [1,4]	not suitable if cannot swallow fluids [4]	DILUTE [4]	-	-	regardless of food [1]	-
			tablet	DISPERSE [4]	can crush and mix with apple puree/ yoghurt [4]	DISPERSE [4]				
R05CB03	**Carbocisteine**	Fludidec	syrup	YES [1,2]	not suitable if cannot swallow fluids [6]	DILUTE [2,3]	-	-	regardless of food [3]	-
R05CB06	**Ambroxol**	Brontex lahus, Flavamed (vaarikas, Forte)	oral solution	YES [1]	dilute [1]<br>not suitable if cannot swallow fluids [6]	DILUTE [6]	-	-	after a meal [1]	the solution can be inhaled (look at SmPC) [1]
		Brontex siirup	syrup		not suitable if cannot swallow fluids [6]					
		Flavamed	effervescent tablet	DIPERGEERI [1]		NO [6]	no info, risk of FT blockage [6]			
		Ambrolan, Brontex Flavamed, Pro-Ambrosan	tablet	NO [1,6]	-					
R05DB09	**Oxeladin**	Paxeladine	syrup	YES [1]	not suitable if cannot swallow fluids [6]	DILUTE [6]	-	-	after a meal [1]	-
**ANTIHISTAMINES FOR SYSTEMIC USE**										
R06AA04	**Clemastine**	Tavegyl	tablet	DISPERSE (takes time) [6]	-	DISPERSE (takes time) [6]	shake while dispersing [6]<br>FT ≥ CH10 [6]	-	before a meal [1]	-
R06AE07	**Cetirizine**	Zyrtec	oral drops	YES [1,2]	can dilute with water [1]<br>not suitable if cannot swallow fluids [6]	DILUTE [2,3,4]	FT ≥ CH8 [3]	-	regardless of food [1,3,4,5]	may cause dry mouth and taste disturbances [4]
			oral solution		not suitable if cannot swallow fluids [6]					
		Aceterin (Express)), Alyr, Cetirizin (Actavis), Cetrix, Zyrtec	(film-coated) tablet	DISPERSE (may take time) [4]	can crush and mix with apple puree/ yoghurt [4]	DISPERSE (may take time) [3,4]				
R05AE09	**Levocetirizine**	Xyzal	oral solution	YES [1]	can dilute with water [1]<br>not suitable if cannot swallow fluids [6]	DILUTE [3]	FT ≥ CH8 [3]	-	regardless of food [1,3,5]	-
		Cezera, Xyzal	film-coated tablet	NO [1]	-	NO [1]	-			

ATC code	Active substance	Medicinal product	Dosage form	Oral administration		Administration by FT		Site of absorption/ administration	Administration with food	Remarks
				Guide	Comment	Guide	Comment			
R06AX13	**Loratadine**	Loratadine Actavis, Loratin, Claritin (Express)	tablet	DISPERSE (may take time) [4,6]	can crush and mix with apple puree/ yoghurt [4]	DISPERSE (may take time) [4,6]	FT ≥ CH10 [6]	-	regardless of food [1,3,4,5]	may cause dry mouth [4]
R06AX13	**Ebastine**	Kestine	film-coated tablet	NO [6]	-	NO [6]	-	-	-	**ALTERNATIVE:** change the API [6]
R06AX27	**Desloratadine**	Dasselta, Yosqiero	film-coated tablet	CRUSH [4]	can mix with apple puree/yoghurt [4]	CRUSH [4]	-	-	regardless of food [3,4,5]	may cause dry mouth [4]
R06AX28	**Rupatadine**	Rupafin	oral solution	YES [1]	not suitable if cannot swallow fluids [6]	DILUTE [6]	-	-	regardless of food [1,5]	-
			tablet	NO [6]	-	NO [6]				
R06AX29	**Bilastine**	Opexa	orodispersible tablet	DISPERSE IN MOUTH [1]	can disperse in water [1]	NO [6]	-	-	stop ETF 1h before and 2h after [5]	**ALTERNATIVE:** change the API [6]
			film-coated tablet	NO [6]	-					
**SENSORY ORGANS**										
S01EC01	**Acetazolamide**	Diacarb, Diuramid	tablet	DISPERSE (takes time) [2,5]	bitter [5] can mix with apple puree/yoghurt [4]	DISPERSE (takes time) [2,3]	FT ≥ CH8 [3]	-	with food GIT side effects ↓ [4,5]	may cause taste disturbances [5]
		-	injection solution	DILUTE [2,3]	-	DILUTE [2,3,4]	-			
**VARIOUS**										
V03AE01	**Polystyrene sulfonate**	Sorbisterit	powder for oral/ rectal suspension	YES (look at SmPC) [1,4,5]	can mix with jam/ honey [4] do not mix with fruit juices that are high in potassium [4,5]	DISPERSE [4,5]	20g (one measuring spoon) of powder to 60-150ml [1,4]	not absorbed, local effect in the GIT [1,3,4,5]	regardless of food [3] administer during the main meal [1]	separately from other drugs [1] may be administered rectally [1,4,5]

[1] Estonia State Agency of Medicines, The Register of Medicinal Products, Summaries of Product Characteristics (SmPC); https://www.ravimiregister.ee/; (visited: 06/2021-12/2021)

[2] The NEWT Guidelines for administration of medication to patients with enteral feeding tubes or swallowing difficulties (2nd Edition; http://www.newtguidelines.com/index.html (visited: 10/2021-12/2021))

[3.] White R, Bradnam, V, Handbook of Drug Administration via Enteral Feeding Tubes. 3rd ed. London: Pharmaceutical Press; 2015

[4] Australian AusDi; Don't Rush to Crush (2nd Edition; https://ausdi.hcn.com.au/ (visited: 03/2021-07/2021))

[5] UpToDate®. Drug monographs. UpToDate, Inc.; www.uptodate.com (visited: 07/2021-12/2021)

[6] ESHP working group assessment

[7] Draft NIOSH List of Hazardous Drugs in Healthcare Settings, 2020 (https://www.cdc.gov/niosh/docket/review/docket233c/pdfs/DRAFT-NIOSH-Hazardous-Drugs-List-2020.pdf)

The list of APIs by page number

Active pharmaceutical ingredient	Page number
**Abacavir**	**26**
Aceclofenac	29
Acetazolamide	43
Acetylcysteine	42
Acetylsalicylic acid (+ magnesium oxide)	12
Aciclovir	25
Agomelatine	38
Albendazole	40
Alendronic acid (+ colecalciferol)	30
Alfuzosin	20
Allopurinol	30
Alprazolam	36
Aluminium hydroxide + magnesium hydroxide	7
Amantadine	34
Ambrisentan	15
Ambroxol	42
Aminophylline	41
Amiodarone	14
Amisulpiride	36
Amitriptyline	37
Amlodipine	17
Amoxicillin (+ clavulanic acid)	22
Apixaban	13
Aprepitant	8
Aripiprazole	36
Atenolol	16
Atomoxetine	39
Atorvastatin	18
Azathioprine	28
Azithromycin	23
**Baclofen**	**30**
Betahistine	40
Bilastine	43
Bisacodyl	8
Bisoprolol	16
Bosentan	15
Brivaracetam	34
Bromazepam	36
Bromhexine	42
Bromocriptine	19
Budesonide	9
Bupropione	38
Butylscopolamine	8
**Cabergoline**	**19**
Calcitriol (Vitamin D)	11
Calcium carbonate (+ colecalciferol)	11
Candesartan	18
Captopril	17
Carbamazepine	33
Carbocisteine	42
Carvedilol	16
Cefadroxil	22
Cefprozil	22
Cefuroxime	22
Celecoxib	30
Cetirizine	42
Chlorpromazine	35
Chlorprothixene	35

Active pharmaceutical ingredient	Page number
Ciclosporin	28
Cinnarizine	40
Ciprofloxacin	23
Citalopram	37
Cladribine	27
Clariprazine	36
Clarithromycin	23
Clemastine	42
Clindamycin	23
Clofazimine	25
Clomipramine	37
Clonazepam	32
Clopidogrel	12
Clozapine	35
Codeine + paracetamol	31
Colchicine	30
Colecalciferol (Vitamin D3)	11
Colestyramine	19
**Dabigatran**	**13**
Danazol	19
Dapagliflozin	11
Desloratadine	43
Desmopressin	20
Dexamethasone	20
Dexketoprofen	30
Dexketoprofen + tramadol	32
Diazepam	36
Diclofenac	29
Digoxin	14
Dihydrocodeine	31
Diltiazem	17
Dimethyl fumarate	28
Dolutegravir	26
Domperidon	8
Donepezil	39
Doxazosin	15
Doxycycline	21
Dronedarone	14
Drotaverine	7
Duloxetine	38
Dutasteride	20
Dydrogesterone	19
**Ebastine**	**43**
Edoxaban	13
Empagliflozin	11
Enalapril	17
Entecavir	26
Ergocalciferol (Vitamin D2)	11
Ertugliflozin	11
Escitalopram	38
Esomeprazole	7
Ethambutol	25
Etoricocib	30
Ezetimibe	19
**Febuxostat**	**30**
Felodipine	17
Fenofibrate	19
Fentanyl	31

Active pharmaceutical ingredient	Page number
Ferrous sulphate	13
Fidaxomicin	9
Finasteride	20
Fingolimod	27
Flecainide	14
Fluconazole	24
Fludrocortisone	20
Fluoxetine	37
Flupentixol	35
Fluvastatin	18
Fluvoxamine	38
Folic acid	13
Fosfomycin	24
Fosinopril	18
Furosemide	15
**Gabapentin**	**33**
Gliclazide	10
Glimepiride	10
Glipiside	10
Glyceryl trinitrate	14
Granisetron	8
**Haloperidol**	**35**
Hydrochlorothiazide	15
Hydrocortisone	21
Hydroxychloroquine	40
**Ibandronic acid**	**31**
Ibuprofen	29
Indapamide	15
Indometacin	28
Iron(III)-hydroxide polymaltose complex	13
Isavuconazole	25
Isoniazid	25
Isosorbide dinitrate	14
Isosorbide mononitrate	14
Itraconazole	24
Ivabradine	15
**Ketoconazole**	**24**
Ketoprofen	29
**Labetalol**	**16**
Lacidipine	17
Lactulose	8
Lamivudine	26
Lamotrigine	33
Lansoprazole	7
Leflunomide	27
Lenalidomide	28
Lercanidipine	17
Levetiracetam	33
Levocetirizine	42
Levodopa + benserazide	34
Levodopa + carbidopa	34
Levodopa + carbidopa + entacapone	34
Levofloxacin	23
Levomepromazine	35
Levothyroxine sodium	21
Linagliptin	11
Linezolid	24
Lipase + amylase + protease	10
Lithium	36
Loperamide	9
Loratadine	43
Lorazepam	36
Lornoxicam	29
Losartan	18
**Macrogol**	**9**
Magnesium citrate	12
Mebendazole	40
Mebeverine	7
Mefloquine	40
Melatonin	37
Meloxicam	29
Melperone	35
Memantine	39
Mesalazine	9
Metformin	10
Methadone	40
Methotrexate	28
Methylprednisolone	20
Metoclopramine	8
Metoprolol	16
Metronidazole	24
Metylphenidate	39
Mexiletine	14
Miconazole	24
Midazolam	37
Minocycline	21
Mirtazapine	38
Misoprostol	19
Montelukast	41
Morphine	31
Moxifloxacin	24
Moxonidine	15
Mycophenolic acid	27
**Nabumetone**	**30**
Naftidrofuryl	16
Nalmefene	39
Naloxegol	9
Naproxen (+ esomeprazole)	29
Nebivolol	16
Nicotine	39
Nifedipine	17
Nimodipine	17
Nitrazepam	36
Nitrendipine	17
Nitrofurantoin	24
Norfloxacin	23
Nortriptyline	37
**Ofloxacin**	**23**
Olanzapine	35
Olmesartan	18
Omeprazole	7
Omeprazole	7
Ondansetron	8
Oseltamivir	26
Oxcarbazepine	33
Oxeladin	42
Oxybutynin	19
Oxycodone (+ naloxone)	31
**Pantoprazole**	**7**
Paracetamol	32
Paracetamol + codeine	31
Paracetamol + tramadol	32
Paroxetine	37
Pentoxifylline	16

Active pharmaceutical ingredient	Page number
Perampanel	34
Perindopril	18
Phenobarbital	32
Phenoxymethylpenicillin	21
Phenytoin	32
Phosphate	12
Phospholipids from soybeans	8
Phytomenadione	13
Pioglitazone	10
Piracetam	39
Piroxicam	29
Polystyrene sulfonate	43
Pomalidomide	28
Posaconazole	25
Potassium chloride (+potassium hydrogen carbonate)	12
Pramipexole	34
Pravastatin	18
Prednisolone	21
Pregabalin	33
Primidone	32
Propafenone	14
Propranolol	16
Propylthiouracil	21
Pseudoephedrine (and combinations)	41
Pürazinamide	25
Pyridostigmine	39
**Quetiapine**	**35**
**Racecadotril**	**9**
Raltegravir	26
Ramipril	18
Ranolazine	15
Rasagiline	34
Retinol (Vitamin A)	11
Rifampicin	25
Rifaximin	9
Rilpivirine	26
Riluzole	40
Riociguat	15
Risedronc acid	31
Risperidone	36
Ritonavir	26
Rivaroxaban	13
Roflumilast	41
Ropinirole	34
Rosuvastatin	19
Rupatadine	43
**Salbutamol**	**41**
Saxagliptin	10
Selexipag	13
Sertindole	35
Sertraline	37
Sildenafil	20
Silodosin	20
Simeticone	7
Simvastatin	18
Siponimod	27

Active pharmaceutical ingredient	Page number
Sirolimus	27
Sitagliptin	10
Sodium chloride	12
Sodium picosulfate	8
Sodium valproate (+ valproic acid)	33
Solifenacin	19
Sotalol	16
Spironolactone	16
Stiripentol	34
Strontium ranelate	31
Sulfamethoxazole + trimethoprim	22
Sulfasalazine	9
Sulpiride	35
Sultamicillin	22
Sumatriptan	32
**Tacrolimus**	**28**
Tadalafil	20
Tamsulosin	20
Telmisartan	18
Tenofovir alafenamide	26
Tenofovir disoproxil	26
Teriflunomide	27
Thalidomide	28
Theophylline	41
Thiamazole	21
Tianeptine	38
Ticagrelor	12
Tinidazole	40
Tizanidine	30
Tocopherol (Vitamin E)	11
Tofacitinib	27
Tolterodine	19
Topiramate	33
Torasemide	15
Tramadol (+ paracetamol/ + dexketoprofen)	32
Trandolapril	18
Tranexamic acid	13
Trazodone	38
Trihexyphenidyl	34
Trimetazidine	14
Trimethoprim (+ sulfamethoxazole)	22
**Upadacitinib**	**28**
Ursodeoxycholic acid	8
**Valaciclovir**	**25**
Valganciclovir	26
Valproic acid (+ sodium valproate)	33
Valsartan	18
Venlafaxine	38
Verapamil	17
Vildagliptin	10
Voriconazole	24
Vortioxetine	39
**Warfarin**	**12**
**Zidovudine**	**26**
Zolpidem	37
Zonisamide	33
Zopiclone	37
Zuclopenthixol	35
